# Patient-reported and caregiver-reported outcomes in refractory ascites secondary to cirrhosis: A systematic review and meta-analysis

**DOI:** 10.1097/HC9.0000000000001054

**Published:** 2026-10-14

**Authors:** Yazan Haddadin, James Ryley, Benjaman To, Vasso Anagnostopoulou, Stephen Bremner, Lucia Macken, Sumita Verma

**Affiliations:** 1 Brighton and Sussex Liver Research Centre, Department of Clinical and Experimental Medicine, Brighton and Sussex Medical School, University of Brighton and University of Sussex, Brighton, UK; 2 Department of Gastroenterology and Hepatology, University Hospitals Sussex NHS Foundation Trust, Sussex County Hospital, Brighton, UK; 3 Department of Primary Care and Public Health, Brighton and Sussex Medical School, University of Brighton and University of Sussex, Brighton, UK; 4 Division of Methodologies, Faculty of Nursing, Midwifery and Palliative Care, King’s College London, London, UK

**Keywords:** end-stage liver disease, health-related quality of life, patient-reported outcomes

## Abstract

**Background:**

Refractory ascites (RA) secondary to advanced chronic liver disease (ACLD) results in poor health-related quality of life (HRQL), high symptom burden, and caregiver workload. Patient/caregiver-reported outcomes (PROs) remain uncharacterized in this cohort. This review summarises the available evidence on this topic.

**Methods:**

MEDLINE/Embase/Cochrane/PsycINFO/Web of Science/CINAHL were searched for articles that included PROs in RA secondary to ACLD. A structured narrative synthesis and a meta-analysis were undertaken.

**Results:**

Sixteen articles were included, comprising 1040 patients and 47 caregivers. Fourteen PRO measures were used, most commonly the Short Form-36 (SF-36) and the Chronic Liver Disease Questionnaire (CLDQ). Domain scores were pooled from 4 SF-36 and 3 CLDQ studies. Pooled domain scores were lower than those reported in stable outpatients with cirrhosis in 3/8 SF-36 domains: Social functioning (mean difference −18.8 [95% CI: −30.2 to −7.3]), Mental health (−17.9 [−33.5 to −2.3]), and Physical functioning (−13.6 [−24.5 to −2.7]); and 4/6 CLDQ domains: Abdominal symptoms (−1.5 [−2.3 to −0.7]), Activity (−0.9 [−1.4 to −0.5]), Worry (−1.2 [−2.1 to −0.3]), and Emotional function (−0.7 [−1.3 to −0.1]). Disease-specific tools had broader domain coverage and were more sensitive at picking up impairments in HRQL compared with generic ones. One symptom tool (the Ascites Questionnaire) had been validated in RA. The Short-Form Liver Disease Quality of Life questionnaire (SFLDQoL), the only HRQL measure validated in decompensated ACLD, was used once. Only 2 studies assessed informal caregivers.

**Conclusions:**

RA in ACLD detrimentally impacts HRQL across multiple domains. However, the lack of validated tools remains a barrier to assessing PROs and conducting well-designed RCTs in this cohort. This review highlights potential domains for the future development of such tools, which could drive equitable, patient-centered care in ACLD.

## INTRODUCTION

Refractory ascites (RA) is a challenging complication of advanced chronic liver disease (ACLD), with only a minority of patients being eligible for either liver transplantation (LTx) or a transjugular intrahepatic portosystemic shunt (TIPS).^1^,^2^,^3^ Transplant-free survival is estimated to be between 6 and 12 months, with mortality being as high as 80% at 5 years.^4^

The current standard of care for RA is large-volume paracentesis (LVP). This provides symptomatic relief but is not curative.^1^ The repeated build-up of ascites is associated with increasing symptom burden.^5^ To access hospital drainage, patients often have to navigate complex secondary care pathways, at the same time as having to come to terms with a life-limiting illness and the prospect of death.^6^

Beyond patients, informal caregivers of patients with ACLD are also affected by the burden of the disease.^7^,^8^ Studies demonstrate higher caregiver burden, lower health-related quality of life (HRQL), and higher incidence of psychological distress among caregivers compared with the general population, with ascites being a significant driver of all 3.^7^,^8^

Despite RA being recognized as a prompt to shift toward symptom-based management, timely linkage to palliative care (PC) remains suboptimal in ACLD.^2^,^3^ This denies patients and caregivers access to holistic care, which could alleviate symptoms and manage uncertainty associated with an incurable condition.^9^ This gap in clinical care is mirrored in research with a disproportionate focus on procedural outcomes and clinical endpoints. In a recent review of therapeutic interventions for RA, only 3 out of 77 studies looked at the impact on HRQL.^10^

Patient-reported outcome (PRO) measures are tools that measure symptoms, function, or well-being in relation to a health condition, from the perspective of patients, without interpretation by health-care professionals.^11^ These are arguably more relevant for a cohort approaching the end of life.^12^ The lack of validated PRO measures in ACLD, including RA, has been identified as an important barrier for the design and implementation of palliative clinical trials.^13^

Reviewing the current evidence on PROs in RA is a necessary first step toward understanding the lived experience of such patients and could provide the basis for developing the robust outcome measures needed to inform palliative interventions in this marginalized group.^12^

The aims of this systematic review and meta-analysis were therefore to:

- summarize the quality of evidence around PROs in RA;
- examine the most commonly used PRO measures and the domains assessed in each; and
- explore the PRO domains most affected in RA and the impact of non-transplant interventions.

## METHODS

This systematic review followed the Preferred Reporting Items for Systematic Reviews and Meta-Analyses (PRISMA) guidelines (Supplemental Appendix S1, https://links.lww.com/HC9/C490). Since it analysed publicly available secondary data, the review is exempt from formal Insitutional Review Board (IRB) approval. A detailed protocol was prospectively registered and published on PROSPERO (CRD42024432741).^14^

### Search strategy and selection criteria

Six databases (MEDLINE/Embase/Cochrane/PsycINFO/Web of Science/CINAHL) were searched from their inception until May 20, 2026. A peer-reviewed search strategy framework was designed for MEDLINE and adapted for each database (Supplemental Appendix S2, https://links.lww.com/HC9/C490). The search terms followed the patient/intervention/comparator/outcome (PICO) framework. Due to the expected heterogeneity in study design, the “intervention” and “comparator” components of the search were left undefined to preserve the inclusivity of the search results.

Original articles (in English), irrespective of study design, that included adult patients (aged ≥18 y) with RA secondary to ACLD, providing data on patient/caregiver-reported outcomes were included. Studies that did not specifically mention RA were excluded. Authors of studies published after 2010 were contacted to provide further data if necessary. Reference articles of identified reviews were screened, and major journal websites were also hand-searched. Studies were classified as interventional versus observational and further grouped by the type of intervention.

### Data extraction

Search results were combined and uploaded onto a systematic review platform, Rayyan.^15^ Duplicates were identified and manually removed. Two independent reviewers (Yazan Haddadin and James Ryley) screened titles, abstracts, and full texts against pre-defined inclusion and exclusion criteria. Disagreements were resolved by consensus. Data extraction forms were agreed a priori by 2 reviewers (Yazan Haddadin and Sumita Verma) and extracted by 1 (Yazan Haddadin). Extracted data were subsequently verified by 2 independent reviewers (Lucia Macken and Sumita Verma).

### Study outcomes

The primary outcome was any reported PRO score at baseline and follow-up timepoints. Other variables assessed included study design, patient demographics, RA definition, cirrhosis etiology, prevalence of decompensation events, inclusion or exclusion criteria, primary study outcomes, presence of control groups, identified determinants of PROs, and treatment effect measures influencing PROs where available (eg, odds ratios, hazard ratios with 95% confidence intervals, and/or *p*-values). If PRO scores were only provided in figures, authors were contacted to provide numerical scores, or a web tool was used to extract values of interest from high-quality graphs if applicable.^16^

### Risk of bias assessments

Eligible studies were evaluated for risk of bias using the relevant Joanna Briggs Institute (JBI) checklist according to study type.^17^ All quality assessments were conducted independently by 2 reviewers (James Ryley and Yazan Haddadin). Disagreements were resolved by consensus and arbitrated by a third reviewer (Sumita Verma) if necessary. In anticipation of the paucity of data, no study was excluded based on the risk of bias assessment.

### Data analysis

To provide a framework for the narrative synthesis, a list of PRO domains was adapted from the Patient-Reported Outcomes Measurement Information System (PROMIS) a priori.^18^ Identified PRO measures from the literature were deconstructed into their constituent items and mapped onto these domains. This process was carried out by 2 independent reviewers (Yazan Haddadin and Benjaman To), and disagreements were resolved through discussion. This methodology was adapted, with permission, from Singh et al.^19^

Where 3 or more studies reported the same PRO scores at baseline or used the same PRO in the assessment of the same intervention, a meta-analysis was carried out to provide an estimate of: (i) HRQL domain scores or (ii) treatment effect measures. Sample means and standard deviations were estimated from reported median/interquartile range (IQR) using established formulas.^20^,^21^ A random-effects meta-analysis was conducted in R statistical software (v4.5.3) using the metafor package (v4.8.0), and mean values were pooled using the restricted maximum likelihood (REML) meta-analysis model.^22^,^23^ The *I* ^2^ statistic was used to quantify heterogeneity across studies, with values >75% suggesting high heterogeneity.^24^ No subgroup analysis was planned in the meta-analysis. Where published data for the same PRO instrument were available, pooled estimates for RA were compared with scores reported from cohorts with stable cirrhosis. Statistical comparisons were interpreted cautiously because of clinical and methodological differences between cohorts.

## RESULTS

### Search results

Out of 2708 articles identified, 196 were eligible for full-text review, and 16 studies (including one conference abstract) met our eligibility criteria:^25^,^26^,^27^,^28^,^29^,^30^,^31^,^32^,^33^,^34^,^35^,^36^,^37^,^38^,^39^,^40^ 6 randomized controlled trials (RCTs),^26^,^27^,^32^,^33^,^34^,^35^ 3 quasi-experimental studies,^28^,^29^,^31^ 5 prospective cohort studies,^25^,^30^,^36^,^38^,^39^ and 2 cross-sectional studies.^37^,^40^ Figure 1 shows the PRISMA flowchart, and Supplemental Appendix S3, https://links.lww.com/HC9/C490, details the excluded full texts. Out of studies involving interventions, 2 investigated TIPS,^3^,^25^,^26^ 3 looked at the automated low-flow ascites pump (ALFA pump),^27^,^28^,^29^ 2 at cell-free and concentrated ascites reinfusion therapy (CART),^30^,^31^ 2 at continuous terlipressin infusion (CTI),^32^,^33^ 2 at long-term abdominal drains (LTAD),^34^,^35^ and 1 at the impact of LVP.^36^ Primary outcomes were PROs in 5 studies,^25^,^36^,^37^,^38^,^40^ but in none of the RCTs.

**FIGURE 1:**
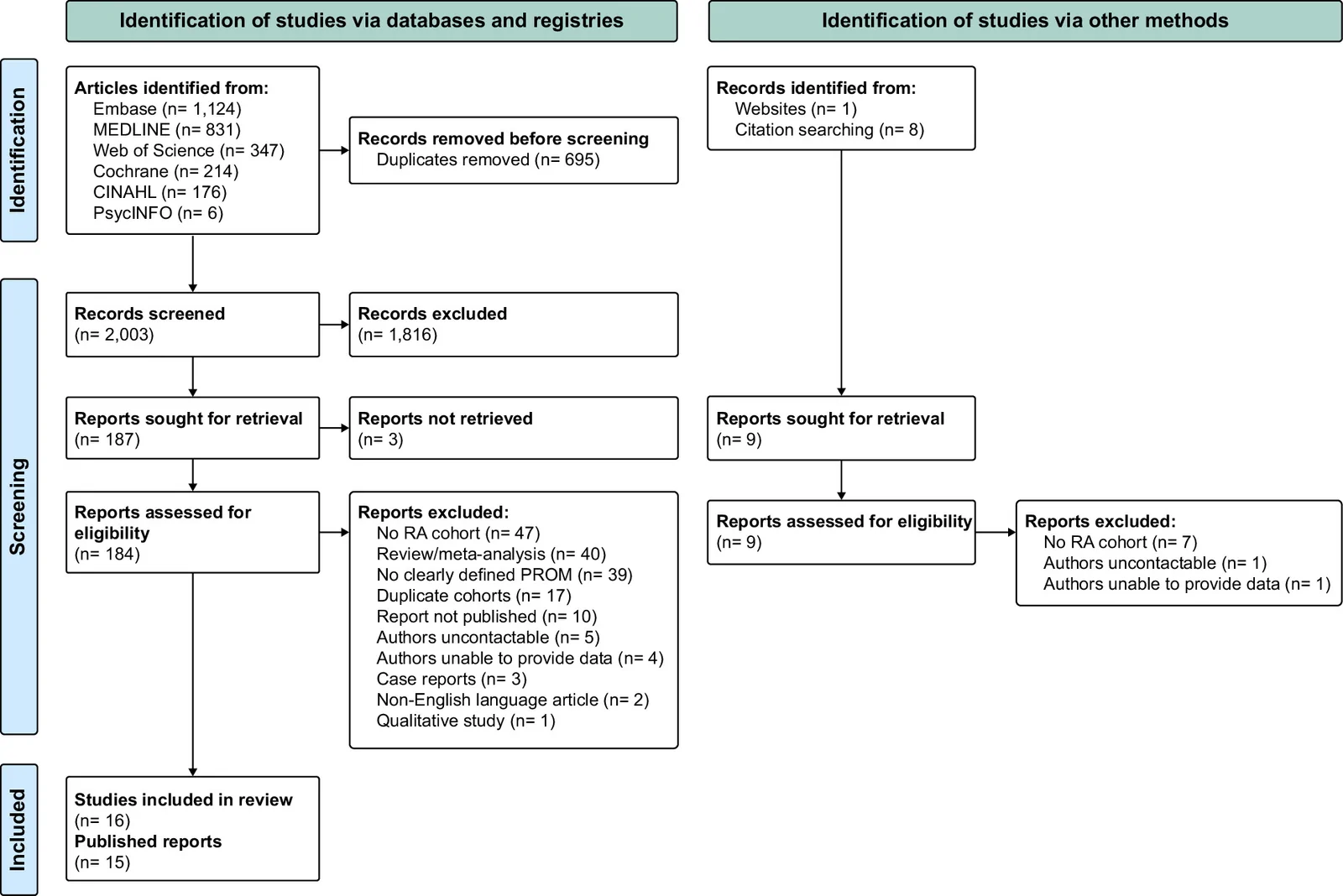
PRISMA flow diagram. This illustrates the study selection process for the systematic review, with the number of results at each stage and the reasons for exclusion. One study included in the review (Higuera-de la Tijera 2019)^40^ was only published as a conference abstract. Abbreviations: PROM, patient-reported outcome measure; RA, refractory ascites.

In total, the studies reported on 1040 patients with RA (and 47 caregivers), although the definition and eligibility thresholds for LVP frequency and volume varied (Table 1). The mean/median ages of the study populations ranged from 48 to 72. Cohorts were majority male (range 52%–86%) except in one, where 54% were female.^34^ The most common etiology for liver disease was alcohol (11 studies^25^,^26^,^27^,^29^,^31^,^32^,^33^,^34^,^35^,^36^,^38^). MELD was the most consistently reported prognostic score, the mean/median ranging between 11 and 16, except in one study where it was 21.^37^ Two studies had a high prevalence of Child–Pugh C disease.^30^,^39^ Study protocols often excluded patients based on a variety of ACLD complications (Table 1). Only one study focused on patients who were ineligible for a liver transplant/TIPS.^35^

**TABLE 1:** Summary of eligible studies included in the systematic review

	First author (y)	Study design/country	Primary outcome	PRO tool and timing	Total RA cohort (N); %Male age (mean/median)	Relevant inclusion criteria	Key exclusion criteria
TIPS	Gülberg (2002)^25^	Prospective cohort study; Single center—Germany	HRQL	*Non-validated PRO* (0–100 VAS for specific domains) BL/3/6 mo	21 86% Mean: 56	LVP requirements for at least 12 mo	Moderate HE HCC; CHF; Ongoing alcohol use
	Campbell (2005)^26^	RCT (TIPS vs. SOC); Multicenter— USA/Canada	Recurrence of ascites/ mortality	*SF-36* BL/6/12 mo	106 66% Mean: 54	Protocol for diuretic therapy prior to enrollment; Drainage volumes (≥10 L in 2 mo)	Life-limiting diagnoses; HE ≥ grade 2; HCC; CHF; Portal vein thrombosis; Renal disease; Concurrent GI bleeding
ALFA pump	Stepanova (2018)^27^	RCT (ALFA pump vs. SOC); Multicenter— USA, France, UK, Austria, Spain, Italy	LVP-free survival	*SF-36* *CLDQ* BL/1/6 mo	58 79% Mean: 62	No specific LVP criteria for inclusion	Recent GI bleed; Recurrent SBP; Loculated ascites, HCC exceeding Milan criteria
	Wong (2020)^28^	Prospective single-arm interventional trial; Multicenter—USA/Canada	Safety profile of the ALFA pump	*CLDQ* *Ascites-Q* BL/1/3/6/9/12 mo	30 57% Mean: 60	≥1 LVP/month for 2 of the preceding 3 mo despite diuretics	Loculated ascites; Recurrent infections; CKD; Moderate HE; SBP; Prognosis <3 mo; MELD ≥21; TIPS eligibility
	Wong (2025)^29^	Prospective single-arm crossover; ​​​​​Multicenter—USA/Canada	Reduction in the number of LVPs	*SF-36* *Ascites-Q* BL/6 mo	40 65% Mean: 63	3-month observation period prior to enrollment; RA defined as ≥2 LVPs in the preceding 30 d	Loculated ascites; Recent infection; HCC exceeding Milan criteria; MELD >20; CKD; Prognosis <6 mo; TIPS eligibility
CART	Yorioka (2023)^30^	Prospective cohort study; Single center—Japan	Safety/efficacy of CART	*ASI-7* Pre/post-CART	23 70% Mean: 72	NR—unclear definition of RA	Acute LVP-related complications; SBP
	Hanai (2022)^31^	Quasi-experimental (CART vs. SOC); Multicenter—Japan	Safety/efficacy of CART	*CLDQ* *ASI-7* BL/8 wk	25 52% Median: 63	Protocol for diuretic/tolvaptan therapy prior to recruitment	CHF; Active infection; SBP; HCC tumor thrombosis; Dialysis; HE; Recent GI bleed prognosis <3 mo
CTI	Terbah (2024)^32^	Randomized crossover study (CTI vs. SOC); Single center—Australia	Change in handgrip strength/ change in LVP volume	*CLDQ* BL/3/6 mo	30 73% Median: 62	No LVP requirements for inclusion	Significant cardiovascular comorbidities; Current or recent alcohol use; Low handgrip strength
	Bajaj (2025)^33^	RCT (CTI vs. SOC); Multicenter—USA	Safety and efficacy of CTI	*CLDQ* *EQ-5D-5L* BL/14/28 d	15 73% Mean: 61	3–9 LVPs in preceding 60 d, and stable doses of diuretics for ≥10 d prior to consent	Severe renal disease; Urinary Na excretion >100 mmol/d
LTAD	Kimer (2020)^34^	RCT (LTAD vs. SOC); Single center—Denmark	LVP-free survival	*CAS* scale BL/monthly	13 46% Median: 68	Reaccumulation of ascites within 2 wk of LVP	TIPS eligibility; HE; Recent VB; Ongoing infection; Prognosis of <3 mo
LTAD	Macken (2020)^35^	RCT (LTAD vs. SOC); Multicenter—UK	RCT feasibility	*IPOS* BL/2 weekly for 12 wk *SFLDQoL* *ZBI-12* *EQ-5D-5L* BL/4/8/12 wk	36 75% Mean: 68	≥1 LVP per month and at least ≥2 LVPs prior to recruitment	Loculated/chylous ascites; Moderate HE; SBP; LTx/TIPS eligibility; CPS<9
LVP	Neijenhuis (2018)^36^	Prospective cohort; Single center—USA	Symptom burden	*Ascites-Q* *ASI-7* *FACIT-AI* *BL/1 wk*	90 61% Mean: 59	Tense ascites sufficient for LVP	Moderate HE; HCC exceeding Milan criteria; Non-English speakers
Observational	Hansen (2022)^37^	Cross-sectional study (patients- caregiver dyads); Single center—USA	HRQL	*SF-36* *MUIS-A* *MUIS-FM* *MS* *BL*	30 83% Mean: 60	NR- RA subset of study cohort	HCC; Severe hearing impairment; Severe psychiatric illness; MELD<15
	Higuera-de la Tijera (2019)^40^	Cross-sectional; Single center—Mexico	HRQL	*SF-36* BL	100 53% Mean: 58	NR	NR
	Macdonald (2018)^38^	Prospective cohort; Multicenter— 8 countries	HRQL	*SF-36* BL	405 73% Median: 58	LVP of ≥4 L and at least one other LVP in the previous 3 mo; Despite diuretics and salt restriction	Recent VB; SBP; GI bleeding; Moderate HE; CKD; HCC exceeding Milan criteria
	Premkumar (2018)^39^	Prospective cohort; Single center—India	Survival and mortality	*MLHFQ* BL	18 59% Mean: 48	NR-RA subset of study cohort	Age>65; CKD; Malignancy; Severe psychiatric illness; Overt HE

*Notes:* Studies are grouped by intervention category. NR refers to aspects that have not been reported by authors.

Abbreviations: ALFA pump, automated low-flow ascites pump; Ascites-Q, Ascites Questionnaire; ASI-7, Ascites Symptom Inventory-7; BL, baseline; CART, cell-free and concentrated ascites reinfusion therapy; CAS, Cirrhosis-associated Ascites Scale; CHF, congestive heart failure; CKD, chronic kidney disease; CLDQ, Chronic Liver Disease Questionnaire; CPS, Child–Pugh score; CTI, continuous terlipressin infusion; EQ-5D-5L, EuroQol questionnaire; FACIT-AI, Functional Assessment of Chronic Illness Therapy—Ascites Index; GI, gastrointestinal; HE, hepatic encephalopathy; HRQL, health-related quality of life; IPOS, Integrated Palliative care Outcome Scale; LTAD, long-term abdominal drain; LTx, liver transplantation; LVP, large-volume paracentesis; MELD, Model for End-stage Liver Disease; MLHFQ, Minnesota Living with Heart Failure Questionnaire; MS, Mutuality Scale; MUIS-A/FM, Mishel Uncertainty in Illness Scale—Adult/Family Member; PRO, patient-reported outcome; RA, refractory ascites; RCT, randomized controlled trial; SBP, spontaneous bacterial peritonitis; SF-36, Short Form-36; SFLDQoL, Short Form Liver Disease Quality of Life questionnaire; SOC, standard of care; TIPS, transjugular intrahepatic portosystemic shunt; VAS, visual analog scale; VB, variceal bleeding; ZBI-12, Zarit Burden Interview (12-item).

### Risk of bias

The open-label design in all the RCTs and the lack of reporting of the minimal clinically important difference (MCID) of PRO measures used were common sources of bias. The MCID was clearly reported in only one study.^36^ MCID is a key concept in PRO research and represents the smallest change in a score that could be perceived by patients as beneficial or harmful.^41^ Conclusions about the impact of interventions were frequently drawn despite the absence of MCID data. Other issues included small sample sizes and high rates of missingness and attrition. Detailed risk of bias assessments are provided in Figure 2. There was <10% disagreement between the 2 assessors (Yazan Haddadin and James Ryley), which was all resolved by consensus.

**FIGURE 2:**
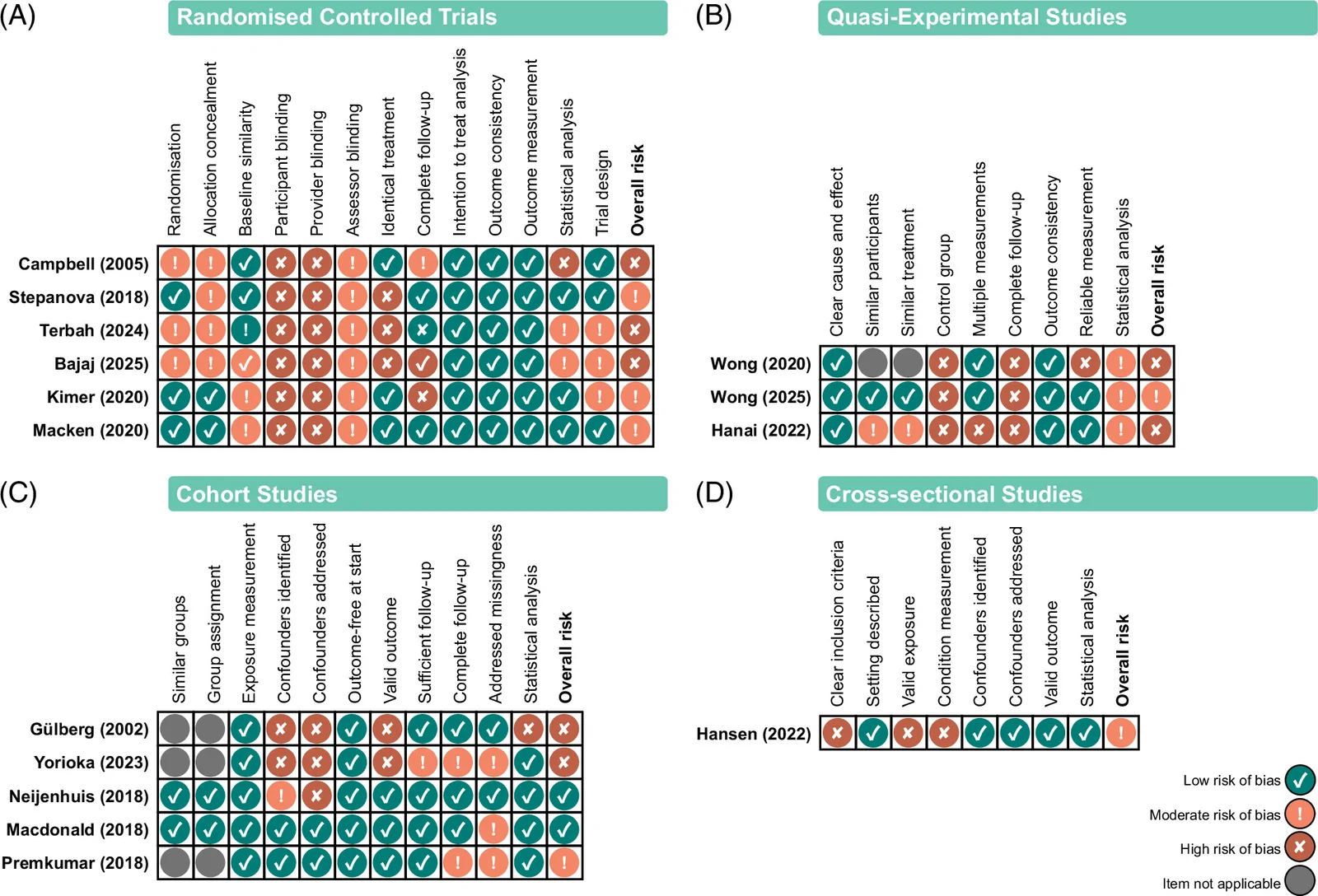
Risk of bias assessment of included studies. Panels (A–D) show the risk of bias assessment in studies grouped by the type of study. The assessment was carried out with the relevant JBI checklist for that study design. The risk of bias was in the context of the patient-reported outcomes of each study. Abbreviation: JBI, Joanna Briggs Institute.

### PRO tools

Across the 16 studies, 14 PRO measures were reported (Supplemental Appendix S4, https://links.lww.com/HC9/C490). The most frequently reported disease-specific HRQL tool was the Chronic Liver Disease Questionnaire (CLDQ)^42^ (n=5); the most frequently reported generic HRQL tool was the Short Form-36 (SF-36) questionnaire (n=6).^43^ Only one study used the Short Form Liver Disease Quality of Life (SFLDQoL) questionnaire,^44^ the only HRQL tool validated in decompensated ACLD (dACLD). The most frequently reported symptom-burden tools were the Ascites Questionnaire (Ascites-Q)^36^ (n=3) and the Ascites Symptom Inventory-7 (ASI-7)^45^ (n=3). The Ascites-Q was the only tool validated specifically in RA. Only 2 studies assessed informal caregivers, one recruiting caregiver-patient dyads in ACLD,^37^ the second focusing on patients with RA.^35^

### Domain coverage and prevalence

The identified PRO tools yielded 122 unique items that mapped onto 15 domains across 4 broad categories. The coverage of each PRO tool against these domains is shown in Figure 3, whereas the prevalence of each domain in the literature is shown in Figure 4. Details of the mapping process are provided in Supplemental Appendix S5, https://links.lww.com/HC9/C490. The SFLDQoL^44^ had the greatest breadth, covering 12 domains. For tools assessing symptom burden, the Integrated Palliative care Outcome Scale (IPOS)^46^ had the broadest coverage (9 domains), followed by the Ascites-Q (7 domains).^36^ Domains, such as physical function and fatigue, were consistently assessed in the literature (domain prevalence 100%), whereas sleep, social isolation/companionship, sexual function, and cognitive function were under-reported (domain prevalence 13%–31%).

**FIGURE 3:**
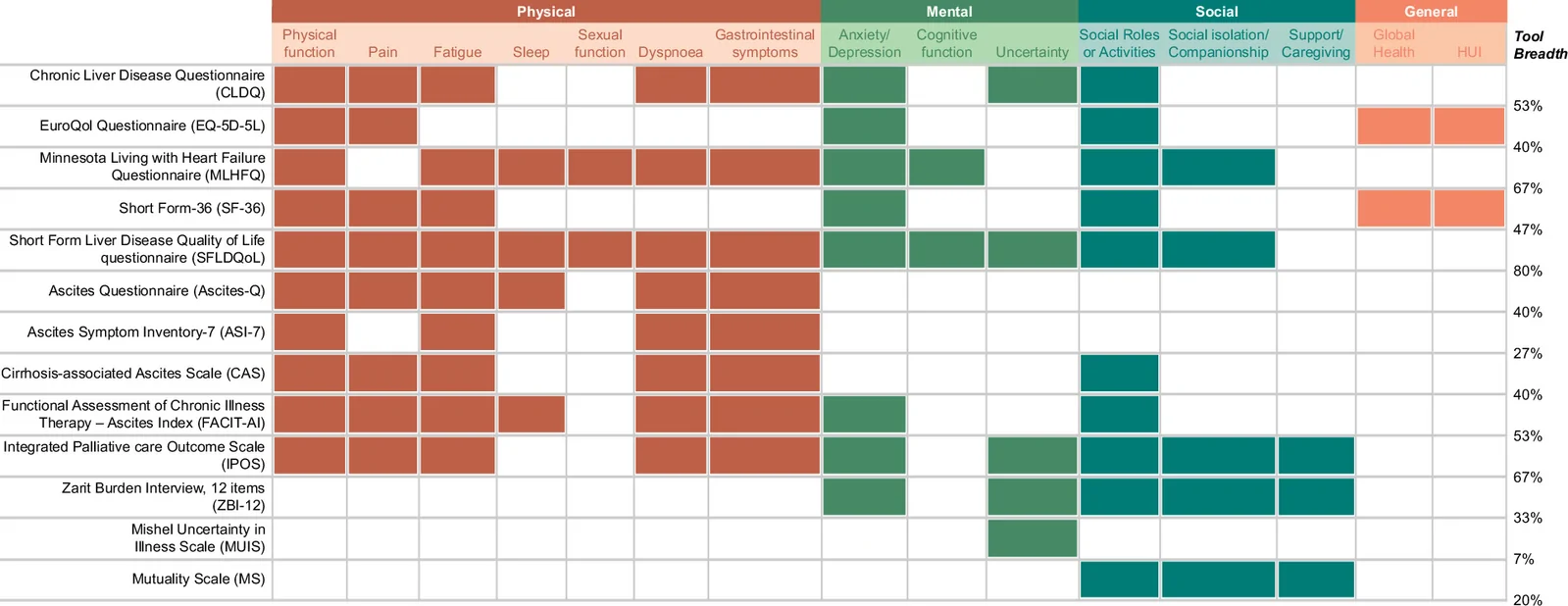
Binary heatmap assessing tool breadth. Each row represents a validated patient (or caregiver)-reported outcome measure used in the included studies. Columns represent relevant domains, extracted from the Patient-Reported Outcome Measurement Information System (PROMIS) framework and grouped into 4 overarching themes (Physical, Mental, Social, and General). A filled cell indicates that a domain is covered by that specific tool. The right-hand column gives the percentage of domains covered by each tool (breadth).

**FIGURE 4:**
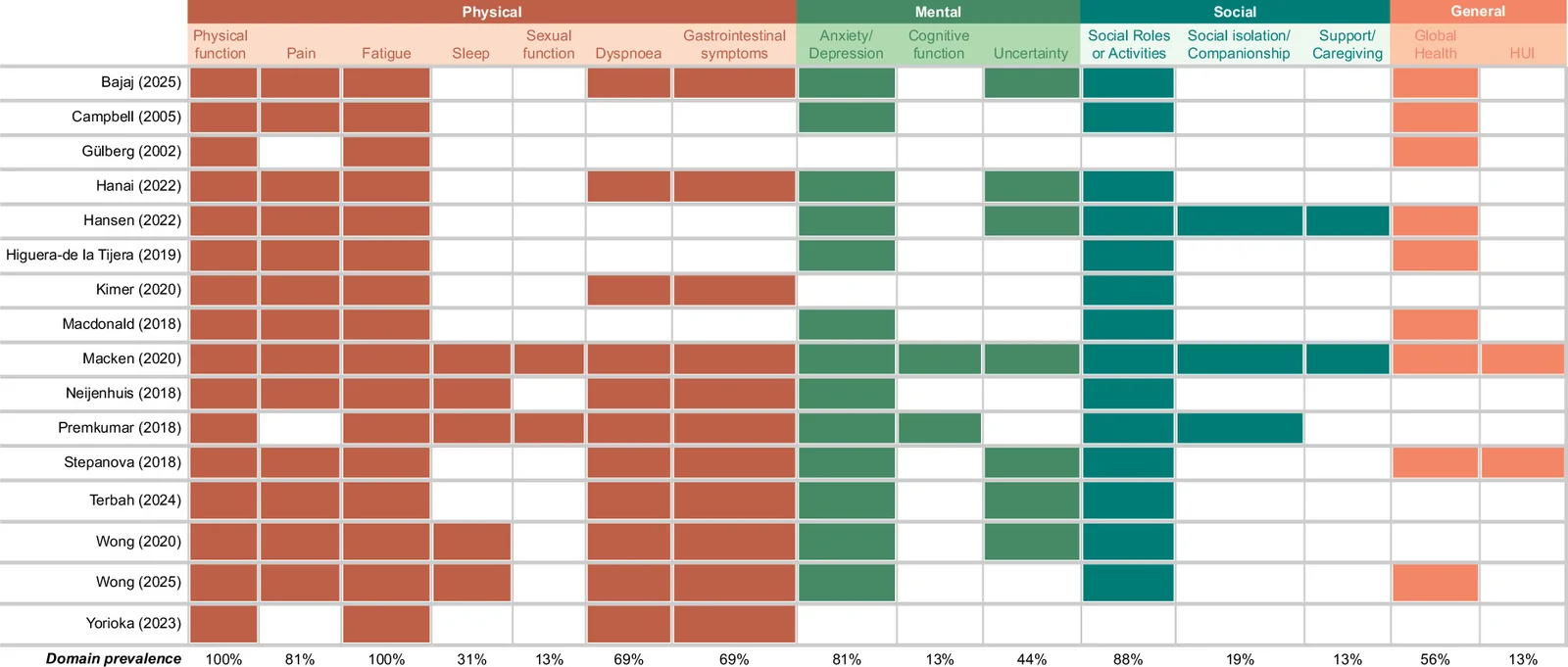
Binary heatmap assessing domain prevalence in the literature. Each row represents a study included in the review. A filled cell indicates that a domain is covered by that study. The bottom row gives the percentage of studies that assessed each domain at least once (domain prevalence).

### Health utility indices

Health utility indices (HUI) are numerical anchored at 0 (representing death) and 1 (representing perfect health) that reflect the health status of an individual.^47^ Scores may be negative for health states considered worse than death. The calculation of HUI often relies on population surveys that use specific PRO tools. However, only 2 studies reported HUIs for patients with RA (mean HUI 0.56–0.58);^27^,^35^ despite studies in the literature using higher estimates (0.6–0.7) when assessing the cost-effectiveness of interventions in RA secondary to ACLD.^48^,^49^,^50^,^51^

### Meta-analysis

Domain scores at baseline were available in 4 out of 6 studies (67%) using the SF-36 tool^27^,^37^,^38^,^40^ and 3 out of 5 studies (60%) using the CLDQ.^27^,^31^,^32^ For both SF-36 and CLDQ, higher scores indicate better HRQL. Pooled SF-36 domain scores were, on average, lower than those reported in a stable outpatient cohort with cirrhosis^52^ in 3 out of 8 domains (Figure 5A): Social functioning (46.1 [95% CI 35.5–56.7] vs. 64.9 [95% CI 60.5–69.2]; mean difference (MD) −18.8 [95% CI −30.2 to −7.3]), Mental health (50.6 [95% CI 35.6–65.6] vs. 68.5 [95% CI 64.3–72.7]; MD −17.9 [95% CI −33.5 to −2.3]), and Physical functioning (37.0 [95% CI 27.3–46.8] vs. 50.6 [95% CI 45.8–55.5]; MD −13.6 [95% CI −24.5 to −2.7]). For all 3 domains, the point estimates of these mean differences exceeded the MCID: Social functioning (18.8 vs. 10.8 points), Mental health (17.9 vs. 7.3 points), and Physical functioning (13.6 vs. 7.3 points). Pooled CLDQ scores were on average lower than the same comparator cohort in 4 out of 6 domains (Figure 5B): Abdominal symptoms (3.5 [95% CI 2.7–4.2] vs. 5.0 [95% CI 4.7–5.2]; MD −1.5 [95% CI −2.3 to −0.7]), Activity (3.8 [95% CI 3.4–4.1] vs. 4.7 [95% CI 4.4–4.9]; MD −0.9 [95% CI −1.4 to −0.5]), Worry (3.5 [95% CI 2.6–4.3] vs. 4.7 [95% CI 4.4–5.0]; MD −1.2 [95% CI −2.1 to −0.3]), and Emotional function (4.1 [95% CI 3.5–4.6] vs. 4.7 [95% CI 4.5–5.0]; MD −0.7 [95% CI −1.3 to −0.1]). The point estimates of these MDs exceeded the MCID for all 4 domains: Abdominal symptoms (1.5 vs. 0.7 points), Activity (0.9 vs. 0.8 points), Worry (1.2 vs. 0.5 points), and Emotional function (0.7 vs. 0.5 points). Detailed methodology for the meta-analysis, and individual forest plots are provided in Supplemental Appendices S6 and S7, https://links.lww.com/HC9/C490.

**FIGURE 5:**
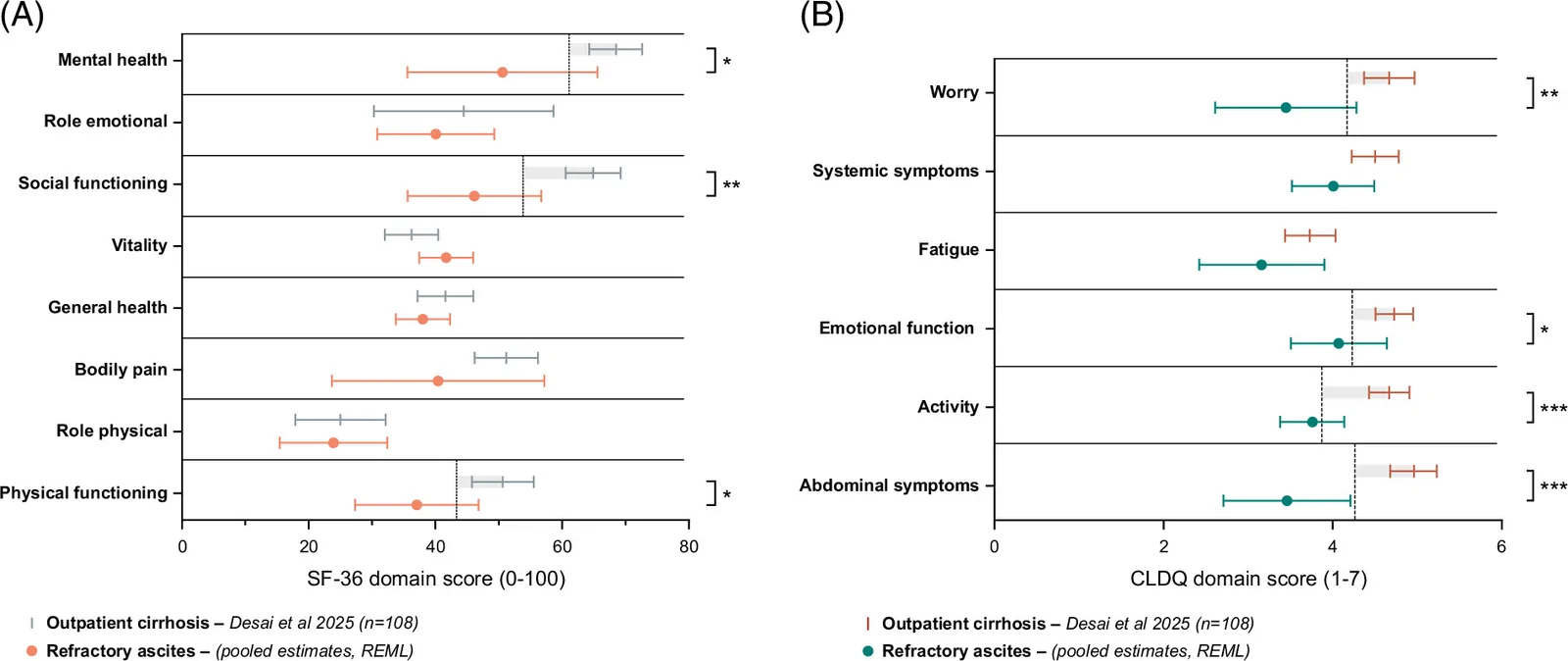
Comparison of health-related quality of life in patients with refractory ascites to those with cirrhosis. Each row shows the pooled point estimate (and 95% confidence intervals) from the meta-analysis of scores in each domain. Lower scores correlate with worse health-related quality of life. Values are compared against reported scores from a cohort of outpatients with cirrhosis. Panel on the left (A) demonstrates disease-specific scores in 8 generic SF-36 domains, and panel on the right (B) shows scores in 6 disease-specific CLDQ domains. The *Z*-statistic was used to test the significance of differences between disease states; ***p<0.001, **p<0.01, and *p<0.05. The shaded gray rectangle highlights the minimal clinically important difference (MCID) for each domain. The point estimates lying outside this zone signal a potential clinically meaningful difference between the 2 disease states. Abbreviations: CLDQ, chronic liver disease questionnaire; REML, restricted maximum-likelihood meta-analysis model; SF-36, short form-36.

### Impact of interventions

The impact of different interventions on PROs is summarized in Table 2. A meta-analysis of treatment effects for each intervention was not appropriate; studies differed substantially in their design, PRO measures, follow-up timepoints, and comparator groups.

**TABLE 2:** Summary of PRO findings by intervention

	Author (y)	BL PRO scores	Reported impact of intervention	PRO determinants	Notes
TIPS	Gülberg (2002)^25^	VAS: 35±25: QoL 25±7: Fatigue	Significant improvement in mean “QoL” VAS rating at 6 mo (66±24 vs. 35±25), *p*=0.02	Therapeutic response (ascites control) associated with improved HRQL	The third PRO planned in the protocol (Physical function) was not reported in the results
			No impact on fatigue scores		
	Campbell (2005)^26^	PCS: 27.6±8.9 MCS: 42.8±11.6	No between-group difference in PCS scores at 12 mo	LVP frequency ≥3, confusion, and hospitalizations were predictors of worse PCS. Confusion was a predictor of worse MCS	51% attrition (26 deaths/27 LTx and 1 withdrawal) out of 106. Inconsistent methodology for multivariate analysis
			Improvement in MCS scores when adjusting for BL rates of confusion (*p*=0.02)		
ALFA pump	Stepanova (2018)^27^	PCS: 35.40±8.99 MCS: 43.25±11.19	Significant improvement in HRQL in certain domains compared with SOC: - Vitality and bodily pain on SF-36 - Abdominal symptoms, activity, and fatigue on CLDQ	Male /alcohol etiology had a detrimental impact on HRQL RA associated with lower HRQL in all domains compared with the general population and in physical domains compared with compensated cirrhosis controls	52% attrition and 28 completed HRQL data at 6 mo sample size. Disease-specific tool (CLDQ) is better than the generic tool (SF-36) at capturing consistent improvements in HRQL
		CLDQ: 4.15±1.21			
		HUI: 0.56±0.11	Unable to assess significance at 6 months due to sample size and attrition		
	Wong (2020)^28^	CLDQ: 3.9±1.2	Significant improvement in total CLDQ score at 3 mo (4.9±1.1, *p*<0.001), but no significant difference beyond that	Control of ascites correlated with improved HRQL and symptom burden	43% attrition with HRQL data available for only 17/30 patients at 1 y
		Ascites-Q: 51.7±21.9	Significant improvement in Ascites-Q score at 3 mo (32.1±18.5, *p*<0.001), maintained up to a year (38.4±18.5, *p*=0.006)		
	Wong (2025)^29^	PCS: 36.6±9.3 MCS: 46.1±11.8	Significant improvement in PCS scores at 6 mo—increase to 42.8± 8.5 (*p*<0.001)	Greatest improvement in HRQL associated with reduction in abdominal distention, loss of abdominal fullness, back pain, and improvement in mobility	Proxy scores by caregivers allowed 26/40 completed 6-month follow-up (2 underwent LTx, 7 explanted devices, and 5 deaths)
			No impact on MCS scores 51.1±8.9 at 6 mo (*p*>0.05)		
		Ascites-Q: 51.0±19.3	Significant improvement in symptom burden, mean Ascites-Q scores 32.2±21.9 at 6 mo (*p*<0.001)		
CART	Yorioka (2023)^30^	ASI-7: 19.8±2.1	Improvement in total ASI-7 score and all domains except for breathing difficulty—total score down to 4.2± 2.2 (*p*=0.002)	NR	Outcomes one day after CART, without long-term data
CART	Hanai (2022)^31^	CLDQ: 3.34 (2.84–4.53)	No significant difference between groups in changes in HRQL	Improved ascites symptom burden correlates with improved HRQL	Treatment allocation determined by a hepatologist at each site. Data available for only 9 patients
		ASI-7: 22 (16–27)			
CTI	Terbah (2024)^32^	CLDQ: 3.28 (2.47–4.16)	HRQL significantly improved across all domains vs. SOC by 0.413 (CI 0.234–0.592)	NR	Participants in the CTI group were visited daily by nurses, vs. once a week in the control group. 19 patients completed 24 wk
	Bajaj (2025)^33^	CLDQ/EQ-5D-5L: NR	Early study termination due to rates of hyponatremia in the CTI group. Inconsistent impact of intervention on HRQL	NR	Under-recruited study that had to be terminated early (15 vs. the target of 30)
LTAD	Kimer (2020)^34^	CAS: 20	Poor scores at baseline. No significant differences between the 2 groups at follow-up	NR	Study terminated early due to slow recruitment (38% attrition) and low rates of completion—(4 in each group)
	Macken (2020)^35^	IPOS: 22.0±9.6	IPOS scores remained consistent in both groups	NR	42% attrition rate. Study not powered to detect differences in PROMs
		SFLDQoL: 56.4±28.0	Liver-specific SFLDQoL scores worsened, especially in the domain of loneliness		
		ZBI-12: 16.3±8.8	Care-giving scores remained stable in the LTAD group vs. worsening burden in the LVP group		
		EQ-5D-5L (HUI): 0.58±0.29	HUI values worsened in the LTAD group, but the VAS showed a trend towards improvement		
LVP	Neijenhuis (2018)^36^	Ascites-Q: 56 (43–69)	LVP was associated with improved Ascites-Q score by >20 points and a significant impact on all domains (except for fatigue) within 7 d LVP-associated with improvement in all 7 domains of the ASI-7 tool and 8/13 domains of the FACIT-AI tool	RA associated with worse symptoms vs. diuretic-controlled ascites on all 3 questionnaires, *p*<0.001. Symptom burden correlated with HRQL but not LVP volume, change in weight, abdominal girth, or MELD	Validation study of the Ascites-Q tool
		ASI-7: 61 (43–75)			
		FACIT-AI: 46 (37–58)			
Observational	Hansen (2022)^37^	PCS: 29.4±8.5 MCS: 40.5±10.3	NA	RA is significantly associated with worse physical QoL compared with cirrhosis without ascites and a worse impact on carers' PCS. HE negatively impacts PCS scores. Younger age is associated with worse MCS scores. Uncertainty around care negatively impacts PCS/MCS scores for both patients/caregivers	RA data provided through author correspondence
		MUIS-A/MUIS-FM: NR			
	Higuera-de la Tijera (2019)^40^	PCS/MCS: NR	NA	Repeated drainage of volumes >5L is associated with worse HRQL in five SF-36 domains compared with those with ascites not requiring drainage	Only published as a conference abstract
	Macdonald (2018)^38^	PCS: 39 (28–52) MCS: 51 (35–71)	NA	A 10-point increase in PCS scores predicted lower mortality (aHR: 0.83 [95% CI: 0.72–0.97]). Leg edema had a negative impact on HRQL (both PCS and MCS)	Patients recruited from the Salvaptan consortium
	Premkumar (2018)^39^	MLHFQ Emotional: 22.6±2.3 Physical: 22.7±2.3	NA	MELD ≥16 (OR: 2.48, *p*=0.029), RA (OR 1.9, *p*=0.05) and worse left ventricular function (OR:1.9, *p*=0.036) were independent predictors of worse HRQL	RA data provided through author correspondence

*Note:* Studies are grouped by intervention. Baseline (BL) scores are presented as mean± standard deviation, or as median (interquartile range). See Supplemental Appendix S4, https://links.lww.com/HC9/C490, for scoring of each tool.

NA: denotes a field that is not applicable, for example, the “impact of intervention” column for observational trials.

Abbreviations: aHR, adjusted hazard ratio; ALFA pump, automated low-flow ascites pump; Ascites-Q, Ascites Questionnaire; ASI-7, Ascites Symptom Inventory-7; CART, cell-free and concentrated ascites reinfusion therapy; CAS, Cirrhosis-associated Ascites Scale; CLDQ, Chronic Liver Disease Questionnaire; CTI, continuous terlipressin infusion; EQ-5D-5L, EuroQol questionnaire; FACIT-AI, Functional Assessment of Chronic Illness Therapy—Ascites Index; HE, hepatic encephalopathy; HRQL, health-related quality of life; HUI, Health Utility Index; IPOS, Integrated Palliative care Outcome Scale; LTAD, long-term abdominal drain; LTx, liver transplantation; LVP, large-volume paracentesis; MCS, mental component summary; MELD, Model for End-stage Liver Disease; MLHFQ, Minnesota Living with Heart Failure Questionnaire; MUIS-A/FM, Mishel Uncertainty in Illness Scale—Adult/Family Member; NR, not reported; OR, odds ratio; PCS, physical component summary; PRO, patient-reported outcome; PROMs, patient-reported outcome measures; QoL, quality of life; RA, refractory ascites; SF-36, Short Form-36; SFLDQoL, Short Form Liver Disease Quality of Life questionnaire; SOC, standard of care; TIPS, transjugular intrahepatic portosystemic shunt; VAS, visual analog scale; ZBI-12, Zarit Burden Interview (12-item).

## DISCUSSION

This is the first systematic review and meta-analysis to focus on PROs in patients with RA secondary to ACLD. Sixteen studies were identified, reporting 14 distinct PRO measures in 1040 patients with RA. Our key findings demonstrate that HRQL in RA is substantially worse than in those with stable cirrhosis, with clinically important differences across multiple SF-36 and CLDQ domains. Most studies used tools that were not validated in RA; consequently, key domains relevant to the lived experience of patients, including sleep, social isolation, and cognition, were inconsistently captured across the literature. In addition, the primary outcomes were PROs in only 5 studies, and in none of the 6 RCTs identified. Together, these findings highlight a discrepancy between the patient-reported burden of disease and the limited extent to which trials were focused on addressing it.

Despite PROs having been recognized as important outcomes for almost 50 years, the published literature in RA due to ACLD remains limited. This is in stark contrast to other areas in hepatology where the evidence base is more established.^53^ Historically, there has been a disproportionate focus on clinical outcomes in this cohort. A recent review identified 47 studies reporting such outcomes following TIPS in RA,^10^ whereas our review identified only 2 reporting PROs for the same intervention (both published over 20 y ago).^25^,^26^ Our results also demonstrate the continued exclusion of RA patients with other ACLD complications, such as hepatic encephalopathy, HCC, and AKI, from clinical trials. This likely overestimates the HRQL of patients with RA, excludes those most likely to benefit from evidence-based palliative interventions, and limits the generalizability of the results. Recent reviews and society guidance have highlighted restrictive inclusion criteria as a barrier to palliative care research in ACLD.^13^,^54^

Our meta-analysis confirms a clinically meaningful, detrimental impact of RA on HRQL. Compared with a stable outpatient cirrhosis cohort, pooled estimates for patients with RA were lower in social functioning, mental health, and physical functioning on the SF-36, and in abdominal symptoms, activity, worry, and emotional function on the CLDQ; point estimates of these differences exceeded published MCIDs, indicating a signal towards clinically meaningful impairment.^52^ High LVP frequency and drainage volumes worsened symptom burden and HRQL scores, whereas relief of ascites, reduced abdominal distention, fullness, and back pain correlated with improved HRQL scores. The capacity to detect these effects, however, depended on the tools used. Disease-specific instruments were more sensitive at picking up impairments in HRQL compared with generic ones. This was evident in our meta-analysis, where RA impacted a higher proportion of domains on the disease-specific CLDQ compared with the generic SF-36 (67% vs. 38%). This was also seen in the ALFA pump RCT, which used both tools and found that CLDQ was more sensitive to intervention-related change than the SF-36.^27^

Although generic tools have some strengths in allowing for cross-disease comparisons, they are susceptible to “floor effects” in severely ill patients, where scores cluster at the lower boundary of the scales, thus reducing the tool’s ability to detect meaningful differences.^55^,^56^ For example, in a large international cohort of over 400 patients with RA, over half scored 0 (the lowest possible score out of 100) on the SF-36 “role physical” domain.^38^ Disease-specific tools, in contrast, are better designed to capture HRQL domains most relevant to patients, and are typically more responsive to disease progression, deterioration, or treatment-led effects.^55^ However, our results demonstrate that domain coverage varies between different disease-specific measures. The CLDQ, for example, which was the most commonly reported disease-specific tool in our review, has not been validated in decompensated cirrhosis and misses important domains relevant to RA, including sleep, cognitive function, social isolation, and companionship. The SFLDQoL, which is the only HRQL tool validated in dACLD, had the greatest breadth in our review and covered these important domains. Despite this, it was reported only in one study and was notably absent from a recent published review of available PROs in liver disease.^35^,^53^ The SFLDQoL is not without its limitations though; it is significantly longer than the CLDQ (72 vs. 29 items) and its validation cohort consisted of patients on the transplant waiting list, whereas the majority of patients with RA are ineligible for this potentially curative pathway.^1^ Taken together, our findings highlight the need for a robust instrument that is narrow enough to limit completion burden, yet broad enough to detect meaningful change from palliative interventions. Developing such a tool can take years,^53^ though our study has highlighted some domains of relevance to patients with ACLD and RA to include Physical and Social functioning, Mental health, Abdominal symptoms, Activity, Worry, and Emotional function.

Synthesizing the evidence around caregiver-reported outcomes in RA was not feasible, as only 2 studies formally assessed them.^35^,^37^ Nonetheless, both highlighted the negative impact of ACLD on the well-being of caregivers. Routine inclusion of caregivers in ACLD trials can improve our understanding of the societal impact of disease, inform study design of community-based interventions, and facilitate study recruitment and participant retention.^6^,^13^ A recently published landmark RCT, looking at the impact of a palliative care intervention on PROs in ACLD, was successful in the recruitment of >900 patients with 60% co-enrollment of caregivers. Retention at 3-months was high, at over 73%.^57^ Despite the significance of this study, unfortunately, we were unable to include it in our systematic review as data on RA were not collected.

Based on our review, no conclusion can be drawn about the impact of any specific RA intervention on PROs, as in most studies, these were secondary endpoints. The interpretation of findings was limited by small sample sizes, high attrition or early study termination, and the lack of comparison with MCIDs to establish whether results were clinically meaningful. Short-term improvements in HRQL domains and symptom burden were observed with the ALFA pump;^27^,^28^,^29^ however, durability and clinical significance were difficult to interpret due to the above-mentioned limitations. The TIPS data was undermined by the use of a non-validated PRO measure,^25^ and by inconsistent statistical methodology.^26^ The CTI trials were limited by early termination and differences in home visit scheduling^32^,^33^ which could have influenced symptoms and HRQL.^58^ The LTAD feasibility trial reported contradictory results across different PRO measures, though it was not powered to detect differences in PRO.^35^ This was likely due to small sample variation and lack of precision. Ongoing trials are looking at the role of interventions, including palliative LTAD and high-dose albumin infusion PROs in RA.^59^,^60^

Our results also have important implications for health economic analyses of interventions in RA. Only 2 studies reported a HUI in patients with RA, with mean values of 0.56 and 0.58.^27^,^35^ Previously published cost-effectiveness analyses of interventions, including TIPS, in this population have used estimates borrowed from other diseases (ranging between 0.6 and 0.7).^48^,^49^,^50^,^51^ Overinflated baseline HRQL for patients with RA would systematically underestimate the incremental benefit of any intervention that has the potential to improve HRQL, and increase the cost of any quality-adjusted life-year gained. Empirically derived HUIs from RA patients, generated alongside interventional trials, would improve the sensitivity analyses of cost-effectiveness studies.

A limitation of this review is that the random-effects meta-analysis model was conducted on a small number of studies (SF-36, n=4; CLDQ, n=3), which limits the reliability of the between-study variance estimate. Furthermore, a high *I* ^2^ statistic suggests PRO domain scores are context-specific and therefore indirect comparison in our meta-analysis should be interpreted cautiously. However, the results of these comparisons were corroborated by findings from the individual studies in the review. Another limitation was the process of mapping PRO tools across PROMIS-based domains, which involved an element of subjectivity, but the effect was mitigated by the use of 2 independent reviewers.

In conclusion, RA in ACLD is associated with significantly impaired PROs across multiple physical, social, and mental domains, with HRQL being substantially worse than in patients with stable cirrhosis. However, the absence of validated, disease-specific HRQL tools remains a barrier to assessing PROs in this cohort and to conducting well-designed RCTs. Our findings reveal a lack of consensus in this area of research and underscore the need for a shared framework and consistent approach to PROs in this cohort. Future research should prioritize the development of such tools in a collaborative effort between the hepatology community and patients/caregivers. This will help drive equitable, patient-centered care for patients and caregivers with RA due to ACLD.

## Supplementary Material

## References

[1] Orman E Fortune B John B . Clinical practice update on the management of ascites, volume overload, and hyponatremia in cirrhosis: Expert review. Gastroenterology. 2025;169:1547–1557. 10.1053/j.gastro.2025.08.029 41114681

[2] English MR Ellis J Verma S Haddadin Y . Outcomes in cirrhosis-related refractory ascites with emphasis on palliative care: Single-centre experience and literature review. Curr Hepatol Rep. 2024;23:316–324. 10.1007/s11901-024-00669-0 PMC7618328 41194902

[3] Macken L Hashim A Potts J Verma S . PWE-069 Care of patients with end stage liver disease and refractory ascites remains suboptimal: Need for earlier input from palliative care. Gut. 2017;66(suppl 2):A161.

[4] Tonon M Piano S Gambino CG Romano A Pilutti C Incicco S . Outcomes and mortality of grade 1 ascites and recurrent ascites in patients with cirrhosis. Clin Gastroenterol Hepatol. 2021;19:358–366.e8. 10.1016/j.cgh.2020.03.065 32272250

[5] Mazumder NR Jezek F Ansari S Tapper EB Lok AS . The physiological determinants of symptom burden in cirrhosis with ascites. United European Gastroenterol J. 2024;12:1222–1229. 10.1002/ueg2.12675 PMC11578835 39377420

[6] Cooper M Pollard A Pandey A Bremner S Macken L Evans CJ . Palliative long-term abdominal drains versus large volume paracentesis in refractory ascites due to cirrhosis (REDUCe study): Qualitative outcomes. J Pain Symptom Manage. 2021;62:312–325.e2. 10.1016/j.jpainsymman.2020.12.007 33348031

[7] Hareendran A Devadas K Sreesh S Tom Oommen T Varghese J Lubina S . Quality of life, caregiver burden and mental health disorders in primary caregivers of patients with cirrhosis. Liver Int. 2020;40:2939–2949. 10.1111/liv.14614 32683772

[8] Nagel M Weidner V Schulz S Marquardt JU Galle PR Schattenberg JM . Continued alcohol consumption and hepatic encephalopathy determine quality of life and psychosocial burden of caregivers in patients with liver cirrhosis. Health Qual Life Outcomes. 2022;20:23. 10.1186/s12955-022-01923-z PMC8822635 35135582

[9] Teno JM Gruneir A Schwartz Z Nanda A Wetle T . Association between advance directives and quality of end-of-life care: A national study. J Am Geriatr Soc. 2007;55:189–194. 10.1111/j.1532-5415.2007.01045.x 17302654

[10] Will V Rodrigues SG Berzigotti A . Current treatment options of refractory ascites in liver cirrhosis—A systematic review and meta-analysis. Dig Liver Dis. 2022;54:1007–1014. 10.1016/j.dld.2021.12.007 35016859

[11] Patrick DL Burke LB Powers JH Scott JA Rock EP Dawisha S . Patient-reported outcomes to support medical product labeling claims: FDA perspective. Value Health. 2007;10(suppl 2):S125–S137. 10.1111/j.1524-4733.2007.00275.x 17995471

[12] Evans CJ Benalia H Preston NJ Grande G Gysels M Short V . The selection and use of outcome measures in palliative and end-of-life care research: The MORECare international consensus workshop. J Pain Symptom Manage. 2013;46:925–937. 10.1016/j.jpainsymman.2013.01.010 PMC3858887 23628515

[13] Verma S Hingwala J Low JTS Patel AA Verma M Bremner S . Palliative clinical trials in advanced chronic liver disease: Challenges and opportunities. J Hepatol. 2023;79:1236–1253. 10.1016/j.jhep.2023.06.018 37419393

[14] PROSPERO . Accessed April 30, 2026. https://www.crd.york.ac.uk/PROSPERO/view/CRD42024432741

[15] Ouzzani M Hammady H Fedorowicz Z Elmagarmid A . Rayyan—A web and mobile app for systematic reviews. Syst Rev. 2016;5:210. 10.1186/s13643-016-0384-4 PMC5139140 27919275

[16] PlotDigitizer . Accessed May 5, 2026. https://plotdigitizer.com/how-to-cite-plotdigitizer

[17] JBI manual for evidence synthesis . Accessed May 5, 2026. https://jbi-global-wiki.refined.site/space/jbi-global-wiki.refined.site

[18] Intro to PROMIS . Accessed May 5, 2026. https://www.healthmeasures.net/explore-measurement-systems/promis/intro-to-promis

[19] Singh J Mehta G Tavabie O . P77 Patient reported outcomes utilised in decompensated cirrhosis: A scoping review. Gut. 2025;74:A65–A66.

[20] Wan X Wang W Liu J Tong T . Estimating the sample mean and standard deviation from the sample size, median, range and/or interquartile range. BMC Med Res Methodol. 2014;14:135. 10.1186/1471-2288-14-135 PMC4383202 25524443

[21] Luo D Wan X Liu J Tong T . Optimally estimating the sample mean from the sample size, median, mid-range, and/or mid-quartile range. Stat Methods Med Res. 2018;27:1785–1805. 10.1177/0962280216669183 27683581

[22] Viechtbauer W . Conducting meta-analyses in R with the metafor package. J Stat Softw. 2010;36:1–48.

[23] R Core Team . R: A Language and Environment for Statistical Computing. Vienna: R Foundation for Statistical Computing; 2021. Accessed May 5, 2026. https://www.scirp.org/reference/referencespapers?referenceid=3131254

[24] Higgins JPT Thompson SG . Quantifying heterogeneity in a meta-analysis. Stat Med. 2002;21:1539–1558. 10.1002/sim.1186 12111919

[25] Gülberg V Liss I Bilzer M Waggershauser T Reiser M Gerbes AL . Improved quality of life in patients with refractory or recidivant ascites after insertion of transjugular intrahepatic portosystemic shunts. Digestion. 2002;66:127–130. 10.1159/000065593 12428073

[26] Campbell MS Brensinger CM Sanyal AJ Gennings C Wong F Kowdley KV . Quality of life in refractory ascites: Transjugular intrahepatic portal-systemic shunting versus medical therapy. Hepatology. 2005;42:635–640. 10.1002/hep.20840 16108073

[27] Stepanova M Nader F Bureau C Adebayo D Elkrief L Valla D . Patients with refractory ascites treated with Alfapump® system have better health-related quality of life as compared to those treated with large volume paracentesis: The results of a multicenter randomized controlled study. Qual Life Res. 2018;27:1513–1520. 10.1007/s11136-018-1813-8 29460201

[28] Wong F Bendel E Sniderman K Frederick T Haskal ZJ Sanyal A . Improvement in quality of life and decrease in large-volume paracentesis requirements with the automated low-flow ascites pump. Liver Transpl. 2020;26:651–661. 10.1002/lt.25724 PMC7216956 31999044

[29] Wong F Vagas HE Reddy KR Pagadala MR Pocha C Sundaram V . The effects of Alfapump on ascites control and quality of life in patients with cirrhosis and recurrent or refractory ascites. Am J Gastroenterol. 2025;120:2291–2301. 10.14309/ajg.0000000000003300 PMC12487658 39760647

[30] Yorioka N Namisaki T Shibamoto A Suzuki J Kubo T Iwai S . Changes in coagulation and fibrinolytic factors in patients with cirrhotic refractory ascites undergoing cell-free and concentrated ascites reinfusion therapy: A retrospective observational study in Japan. In Vivo. 2023;37:1226–1235. 10.21873/invivo.13199 PMC10188040 37103093

[31] Hanai T Kawaratani H Nagano J Suii H Sakamaki A Arase Y . Cell-free and concentrated ascites reinfusion therapy versus large-volume paracentesis for the treatment of cirrhotic patients with refractory ascites: A multicenter prospective observational study. Hepatol Res. 2023;53:238–246. 10.1111/hepr.13860 36433862

[32] Terbah R Testro AG Hoermann R Majumdar A Chapman B Gow PJ . Continuous home terlipressin infusion increases handgrip strength and reduces ascites—A prospective randomized crossover study. Hepatology. 2024;80:605–620. 10.1097/HEP.0000000000000820 38442012

[33] Bajaj JS Weinberg EM Reddy KR Keaveny AP Porayko MK Koch D . Safety and efficacy of continuous infusion terlipressin (BIV201): A phase 2 trial in patients with decompensated cirrhosis and refractory ascites. Liver Transpl. 2025;31:1202–1214. 10.1097/LVT.0000000000000623 PMC12445179 40272365

[34] Kimer N Riedel AN Hobolth L Mortensen C Madsen LG Andersen ML . Tunneled peritoneal catheter for refractory ascites in cirrhosis: A randomized case-series. Medicina (Kaunas). 2020;56:565. 10.3390/medicina56110565 PMC7692861 33121063

[35] Macken L Bremner S Gage H Touray M Williams P Crook D . Randomised clinical trial: Palliative long-term abdominal drains vs large-volume paracentesis in refractory ascites due to cirrhosis. Aliment Pharmacol Ther. 2020;52:107–122. 10.1111/apt.15802 32478917

[36] Neijenhuis M Gevers TJG Atwell TD Gunneson TJ Schimek AC Kievit W . Development and validation of a patient-reported outcome measurement for symptom assessment in cirrhotic ascites. Am J Gastroenterol. 2018;113:567–575. 10.1038/ajg.2018.18 29557942

[37] Hansen L Chang MF Lee CS Hiatt S Firsick EJ Dieckmann NF . Physical and mental quality of life in patients with end-stage liver disease and their informal caregivers. Clin Gastroenterol Hepatol. 2021;19:155–161.e1. 10.1016/j.cgh.2020.04.014 PMC7554075 32289544

[38] Macdonald S Jepsen P Alrubaiy L Watson H Vilstrup H Jalan R . Quality of life measures predict mortality in patients with cirrhosis and severe ascites. Aliment Pharmacol Ther. 2019;49:321–330. 10.1111/apt.15084 30585338

[39] Premkumar M Devurgowda D Vyas T Shasthry SM Khumuckham JS Goyal R . Left ventricular diastolic dysfunction is associated with renal dysfunction, poor survival and low health related quality of life in cirrhosis. J Clin Exp Hepatol. 2019;9:324–333. 10.1016/j.jceh.2018.08.008 PMC6637070 31360025

[40] Higuera-de la Tijera FH Murguía D Montes de Oca Salinas Flor de M. Gómez-Laguna L Ramirez D Oliva IR . Tu1524—Repetitive paracentesis affect quality of life by decreasing physical domains. Gastroenterology. 2019;156:S-1352

[41] Sedaghat AR . Understanding the minimal clinically important difference (MCID) of patient-reported outcome measures. Otolaryngol Head Neck Surg. 2019;161:551–560. 10.1177/0194599819852604 31159641

[42] Younossi ZM Guyatt G Kiwi M Boparai N King D . Development of a disease specific questionnaire to measure health related quality of life in patients with chronic liver disease. Gut. 1999;45:295–300. 10.1136/gut.45.2.295 PMC1727607 10403745

[43] Ware JE Sherbourne CD . The MOS 36-item short-form health survey (SF-36). I. Conceptual framework and item selection. Med Care. 1992;30:473–483. 1593914

[44] Kanwal F Spiegel BMR Hays RD Durazo F Han SB Saab S . Prospective validation of the short form liver disease quality of life instrument. Aliment Pharmacol Ther. 2008;28:1088–1101. 10.1111/j.1365-2036.2008.03817.x 18671776

[45] Onishi Y Wakita T Fukuhara S Noguchi Y Okada M Sakaida I . Development and validation of a symptom scale specific for ascites accompanied with cirrhosis: The ASI-7. Clin Transl Gastroenterol. 2014;5:e48. 10.1038/ctg.2013.20 PMC3940835 24500340

[46] Hearn J Higginson IJ . Development and validation of a core outcome measure for palliative care: The palliative care outcome scale. Palliative Care Core Audit Project Advisory Group. Qual Health Care. 1999;8:219–227. 10.1136/qshc.8.4.219 PMC2483665 10847883

[47] Horsman J Furlong W Feeny D Torrance G . The Health Utilities Index (HUI): Concepts, measurement properties and applications. Health Qual Life Outcomes. 2003;1:54. 10.1186/1477-7525-1-54 PMC293474 14613568

[48] Kwan SW Allison SK Gold LS Shin DS . Cost-effectiveness of transjugular intrahepatic portosystemic shunt versus large-volume paracentesis in refractory ascites: Results of a Markov model incorporating individual patient-level meta-analysis and nationally representative cost data. J Vasc Interv Radiol. 2018;29:1705–1712. 10.1016/j.jvir.2018.08.019 PMC6334842 30392803

[49] Wu X Rabei R Keller EJ King B Kothary N Kohi M . Tunneled peritoneal catheter vs repeated paracenteses for recurrent ascites: A cost-effectiveness analysis. Cardiovasc Intervent Radiol. 2022;45:972–982. 10.1007/s00270-022-03103-4 35292833

[50] Shen NT Schneider Y Congly SE Rosenblatt RE Namn Y Fortune BE . Cost effectiveness of early insertion of transjugular intrahepatic portosystemic shunts for recurrent ascites. Clin Gastroenterol Hepatol. 2018;16:1503–1510.e3. 10.1016/j.cgh.2018.03.027 29609068

[51] Mattock R Tripathi D O'Neill F Craig J Tanner J Patch D . Economic evaluation of covered stents for transjugular intrahepatic portosystemic stent shunt in patients with variceal bleeding and refractory ascites secondary to cirrhosis. BMJ Open Gastroenterol. 2021;8:e000641. 10.1136/bmjgast-2021-000641 PMC8386212 34429322

[52] Desai AP Orman ES Aridi TG Stump T Nephew L Ghabril MS . Meaningful differences in patient-reported outcome measurement scores in liver disease. Hepatol Commun. 2025;9:e0727. 10.1097/HC9.0000000000000727 PMC12122172 40434652

[53] Younossi ZM Stepanova M Schattenberg JM Henry L . Measuring health-related quality of life and patient-reported outcomes in chronic liver disease. J Hepatol. 2026;84:457–472. 10.1016/j.jhep.2025.09.027 41072803

[54] Verma S Pinter M Macken L Künzler-Heule P Gluud LL Takkenberg B . EASL position paper on palliative care in advanced chronic liver disease. JHEP Rep. 2026;8:101686. 10.1016/j.jhepr.2025.101686 PMC13069526 41951279

[55] Higginson IJ Carr AJ . Using quality of life measures in the clinical setting. BMJ. 2001;322:1297–1300. 10.1136/bmj.322.7297.1297 PMC1120388 11375237

[56] Hyland ME . A brief guide to the selection of quality of life instrument. Health Qual Life Outcomes. 2003;1:24. 10.1186/1477-7525-1-24 PMC194862 12848895

[57] Verma M Navarro V Kosinski A Taddei T Kalman R Barritt IV AS . Palliative care intervention for patients with end-stage liver disease: A cluster randomized clinical trial. JAMA Intern Med. 2026;186:677–686. 10.1001/jamainternmed.2026.0571 PMC13077576 41973444

[58] Li L Donlan J Nurhussien L Horick N Zeng C Indriolo T . Prognostic communication, psychological distress, and caregiving burden among caregivers of patients with decompensated cirrhosis. Liver Transpl. 2026;32:27–34. 10.1097/LVT.0000000000000683 40637541

[59] Haddadin Y Anagnostopoulou V Bremner S Harder H Starkings R Lambert D . Palliative long-term abdominal drains vs. large volume paracentesis for refractory ascites secondary to cirrhosis: Protocol for a definitive randomised controlled trial (REDUCe2 study). Trials. 2025;26:193. 10.1186/s13063-025-08873-z PMC12139341 40468365

[60] ClinicalTrials.gov . High Dose Albumin in Refractory Ascites. Identifier: NCT05867602. Accessed May 20, 2026. https://clinicaltrials.gov/study/NCT05867602

