# Supplementary material for: Patient-reported and caregiver-reported outcomes in refractory ascites secondary to cirrhosis: A systematic review and meta-analysis
Source: Hepatol Commun. 2026 Oct 14;10(11):e01054. doi: 10.1097/HC9.0000000000001054 (PMC13668286; doi:10.1097/HC9.0000000000001054)
Supplement: Supplementary file 1 [file hc9-10-e01054-s001.docx]

**Patient and caregiver reported outcomes in refractory ascites secondary to advanced chronic liver disease: a systematic review and meta-analysis**

**Authors:** Yazan Haddadin, James Ryley, Benjaman To, Vasso Anagnostopoulou, Stephen Bremner, Lucia Macken, Sumita Verma

Table of Contents

[**Appendix S1:** PRISMA 2020 MAIN checklist	2](#_Toc232422420)

[**Appendix S2**: Search strategy for each database	6](#_Toc232422421)

[MEDLINE	6](#_Toc232422422)

[Embase	7](#_Toc232422423)

[COCHRANE	8](#_Toc232422424)

[PsycINFO	10](#_Toc232422425)

[Web of Science	12](#_Toc232422426)

[CINAHL	13](#_Toc232422427)

[**Appendix S3:** Reasons for the exclusion of potentially eligible studies	17](#_Toc232422428)

[**Appendix S4:** Details on the PRO tools used in eligible studies	31](#_Toc232422429)

[**Appendix S5**: Detailed mapping process for PRO instruments	34](#_Toc232422430)

[**Appendix S6:** Domain scores in the meta-analysis and extraction methodology	38](#_Toc232422431)

[**Appendix S7:** Forest plots of individual domains from the meta-analysis	40](#_Toc232422432)

[CLDQ:	40](#_Toc232422433)

[SF-36:	45](#_Toc232422434)

[Supplementary References	53](#_Toc232422435)

### **Appendix S1:** PRISMA 2020 MAIN checklist

| **Topic** | **No.** | **Item** | **Location where item is reported** |
| --- | --- | --- | --- |
| **TITLE** |  |  |  |
| **Title** | 1 | Identify the report as a systematic review. | page 1 |
| **ABSTRACT** |  |  |  |
| **Abstract** | 2 | See the PRISMA 2020 for Abstracts checklist |  |
| **INTRODUCTION** |  |  |  |
| **Rationale** | 3 | Describe the rationale for the review in the context of existing knowledge. | page 4 |
| **Objectives** | 4 | Provide an explicit statement of the objective(s) or question(s) the review addresses. | page 4 |
| **METHODS** |  |  |  |
| **Eligibility criteria** | 5 | Specify the inclusion and exclusion criteria for the review and how studies were grouped for the syntheses. | page 6-7 |
| **Information sources** | 6 | Specify all databases, registers, websites, organisations, reference lists and other sources searched or consulted to identify studies. Specify the date when each source was last searched or consulted. | page 6 |
| **Search strategy** | 7 | Present the full search strategies for all databases, registers and websites, including any filters and limits used. | appendix S2 |
| **Selection process** | 8 | Specify the methods used to decide whether a study met the inclusion criteria of the review, including how many reviewers screened each record and each report retrieved, whether they worked independently, and if applicable, details of automation tools used in the process. | page 7 |
| **Data collection process** | 9 | Specify the methods used to collect data from reports, including how many reviewers collected data from each report, whether they worked independently, any processes for obtaining or confirming data from study investigators, and if applicable, details of automation tools used in the process. | page 7 |
| **Data items** | 10a | List and define all outcomes for which data were sought. Specify whether all results that were compatible with each outcome domain in each study were sought (e.g. for all measures, time points, analyses), and if not, the methods used to decide which results to collect. | page 7-8 |
|  | 10b | List and define all other variables for which data were sought (e.g. participant and intervention characteristics, funding sources). Describe any assumptions made about any missing or unclear information. | page 7-8 |
| **Study risk of bias assessment** | 11 | Specify the methods used to assess risk of bias in the included studies, including details of the tool(s) used, how many reviewers assessed each study and whether they worked independently, and if applicable, details of automation tools used in the process. | page 8 |
| **Effect measures** | 12 | Specify for each outcome the effect measure(s) (e.g. risk ratio, mean difference) used in the synthesis or presentation of results. | page 8-9 |
| **Synthesis methods** | 13a | Describe the processes used to decide which studies were eligible for each synthesis (e.g. tabulating the study intervention characteristics and comparing against the planned groups for each synthesis (item 5)). | page 8-9 |
|  | 13b | Describe any methods required to prepare the data for presentation or synthesis, such as handling of missing summary statistics, or data conversions. | page 8 |
|  | 13c | Describe any methods used to tabulate or visually display results of individual studies and syntheses. | page 8-9 |
|  | 13d | Describe any methods used to synthesize results and provide a rationale for the choice(s). If meta-analysis was performed, describe the model(s), method(s) to identify the presence and extent of statistical heterogeneity, and software package(s) used. | page 8-9 |
|  | 13e | Describe any methods used to explore possible causes of heterogeneity among study results (e.g. subgroup analysis, meta-regression). | page 9 |
|  | 13f | Describe any sensitivity analyses conducted to assess robustness of the synthesized results. | page 9 |
| **Reporting bias assessment** | 14 | Describe any methods used to assess risk of bias due to missing results in a synthesis (arising from reporting biases). | NA |
| **Certainty assessment** | 15 | Describe any methods used to assess certainty (or confidence) in the body of evidence for an outcome. | Page 9 |
| **RESULTS** |  |  |  |
| **Study selection** | 16a | Describe the results of the search and selection process, from the number of records identified in the search to the number of studies included in the review, ideally using a flow diagram. | Page 9 and figure 1 |
|  | 16b | Cite studies that might appear to meet the inclusion criteria, but which were excluded, and explain why they were excluded. | Appendix S3 |
| **Study characteristics** | 17 | Cite each included study and present its characteristics. | Table 1 |
| **Risk of bias in studies** | 18 | Present assessments of risk of bias for each included study. | Figure 2 |
| **Results of individual studies** | 19 | For all outcomes, present, for each study: (a) summary statistics for each group (where appropriate) and (b) an effect estimate and its precision (e.g. confidence/credible interval), ideally using structured tables or plots. | Table 2 |
| **Results of syntheses** | 20a | For each synthesis, briefly summarise the characteristics and risk of bias among contributing studies. | Page 10 |
|  | 20b | Present results of all statistical syntheses conducted. If meta-analysis was done, present for each the summary estimate and its precision (e.g. confidence/credible interval) and measures of statistical heterogeneity. If comparing groups, describe the direction of the effect. | Page 11-12, Appendix S7 and Figure 2 |
|  | 20c | Present results of all investigations of possible causes of heterogeneity among study results. | NA |
|  | 20d | Present results of all sensitivity analyses conducted to assess the robustness of the synthesized results. | NA |
| **Reporting biases** | 21 | Present assessments of risk of bias due to missing results (arising from reporting biases) for each synthesis assessed. | NA |
| **Certainty of evidence** | 22 | Present assessments of certainty (or confidence) in the body of evidence for each outcome assessed. | Page 12 |
| **DISCUSSION** |  |  |  |
| **Discussion** | 23a | Provide a general interpretation of the results in the context of other evidence. | Page 12 |
|  | 23b | Discuss any limitations of the evidence included in the review. | Page 17 |
|  | 23c | Discuss any limitations of the review processes used. | Page 17 |
|  | 23d | Discuss implications of the results for practice, policy, and future research. | Page 13-15 |
| **OTHER INFORMATION** |  |  |  |
| **Registration and protocol** | 24a | Provide registration information for the review, including register name and registration number, or state that the review was not registered. | Page 6 |
|  | 24b | Indicate where the review protocol can be accessed, or state that a protocol was not prepared. | Page 6 |
|  | 24c | Describe and explain any amendments to information provided at registration or in the protocol. | NA |
| **Support** | 25 | Describe sources of financial or non-financial support for the review, and the role of the funders or sponsors in the review. | Page 2 |
| **Competing interests** | 26 | Declare any competing interests of review authors. | Page 2 |
| **Availability of data, code and other materials** | 27 | Report which of the following are publicly available and where they can be found: template data collection forms; data extracted from included studies; data used for all analyses; analytic code; any other materials used in the review. | Page 2 |

*From:* Page MJ, McKenzie JE, Bossuyt PM, Boutron I, Hoffmann TC, Mulrow CD, et al. The PRISMA 2020 statement: an updated guideline for reporting systematic reviews. MetaArXiv. 2020, September 14. DOI: 10.31222/osf.io/v7gm2. For more information, visit: <www.prisma-statement.org>

**Appendix S2**: Search strategy for each database

### MEDLINE

1	ascites/

2	ascites.ti,ab,kf.

3	1 or 2

4	(refractory or severe or repeated or recurrent or diuretic-intolerant or "diuretic intolerant" or "diuretic resistant" or diuretic-resistant or persistent or untreatable or end-stage or "end stage").ti,ab,kf.

5	3 and 4

6	exp hypertension, portal/

7	exp hepatitis, chronic/

8	exp liver cirrhosis/

9	("portal hypertens*" or "cruveilhier baumgarten syndrome" or "cruveilhier-baumgarten syndrome").ti,ab,kf.

10	(cirrhos* or cirrhot* or liver).ti,ab,kf.

11	"biliary cholang*".ti,ab,kf.

12	("chronic hepatit*" or "persistent hepatit*" or "chronic active hepatit*").ti,ab,kf.

13	"chronic delta hepatit*".ti,ab,kf.

14	or/6-13

15	5 and 14

16	"quality of life"/

17	("quality of life" or HRQOL or qol or "life quality").ti,ab,kf.

18	("patient reported outcome*" or "patient-reported outcome*").ti,ab,kf.

19	patient reported outcome measures/

20	quality-adjusted life years/

21	(qualy* or qaly* or "adjusted life year*" or "quality-adjusted life year*" or "healthy years equivalent*").ti,ab,kf.

22	(daly* or "disability-adjusted life year*").ti,ab,kf.

23	"activities of daily living"/

24	(adl or adls or "activit* of daily living*" or "activit* daily living*" or "daily living activit*" or "chronic limitation* of activit*").ti,ab,kf.

25	("health utilit*" or "health level*" or "health status" or "level of health").ti,ab,kf.

26	health status/

27	exp "surveys and questionnaires"/

28	exp "costs and cost analysis"/

29	(cost-effectiveness or "cost effectiveness" or "cost of illness" or "cost analysis" or "cost-benefit analysis" or "health care costs").ti,ab,kf.

30	(sf-36 or "short-form 36" or "short form 36" or short-form-36 or "short form-36").ti,ab,kf.

31	("short form health survey-8" or sf-8 or "short-form health survey 8" or short-form health survey-8 or "short form health survey 8" or "sf 8").ti,ab,kf.

32	(rand-36 or "rand 36 item-health survey" or rand36).ti,ab,kf.

33	("sickness impact profile" or sip or sip-68 or sip68).ti,ab,kf.

34	(euro-qol or eq-5d-5l or eq-5d-3l or eq-5d-y or eq-vas or "visual analogue scale" or vas).ti,ab,kf.

35	("chronic liver disease questionnaire" or "cldq").ti,ab,kf.

36	("short form liver" or sf-ldqol or sfldqol).ti,ab,kf.

37	("functional assessment of cancer therapy" or "fact-hep").ti,ab,kf.

38	("functional assessment of chronic illness therapy— palliative care" or facit-pal).ti,ab,kf.

39	("palliative care outcome scale" or ipos or pos-s or pos).ti,ab,kf.

40	("ascites questionnaire" or ascites-q or ascitesq).ti,ab,kf.

41	("ascites symptoms inventory" or asi-7 or asi7).ti,ab,kf.

42	("edmonton symptom assessment scale" or esas*).ti,ab,kf.

43	exp caregivers/

44	"caregiver burden"/

45	caregiver*.ti,ab,kf.

46	("zarit burden interview" or zbi-12 or zbi12).ti,ab,kf.

47	("caregiver roles and responsibility scale" or crrs or "caregiver reaction assessment" or cra).ti,ab,kf.

48	("patient health questionnaire-9" or phq-9 or phq9 or hads-d or hads-a or hadsd or hadsa or hads or "hospital anxiety and depression scale").ti,ab,kf.

49	16 or 17 or 18 or 19 or 20 or 21 or 22 or 23 or 24 or 25 or 26 or 27 or 28 or 29 or 30 or 31 or 32 or 33 or 34 or 35 or 36 or 37 or 38 or 39 or 40 or 41 or 42 or 43 or 44 or 45 or 46 or 47 or 48

50	15 and 49

### Embase

1	exp ascites/

2	ascites.ti,ab,kf.

3	1 or 2

4	(refractory or severe or repeated or recurrent or diuretic-intolerant or "diuretic intolerant" or "diuretic resistant" or diuretic-resistant or persistent or untreatable or end-stage or "end stage").ti,ab,kf.

5	3 and 4

6	exp portal hypertension/

7	exp chronic hepatitis/

8	exp liver cirrhosis/

9	("portal hypertens*" or "cruveilhier baumgarten syndrome" or "cruveilhier-baumgarten syndrome").ti,ab,kf.

10	(cirrhos* or cirrhot* or liver).ti,ab,kf.

11	"biliary cholang*".ti,ab,kf.

12	("chronic hepatit*" or "persistent hepatit*" or "chronic active hepatit*").ti,ab,kf.

13	"chronic delta hepatit*".ti,ab,kf.

14	or/6-13

15	5 and 14

16	exp "quality of life"/

17	("quality of life" or hrqol or qol or "life quality").ti,ab,kf.

18	("patient reported outcome*" or "patient-reported outcome*").ti,ab,kf.

19	exp patient-reported outcome/

20	exp disease burden/

21	(qualy* or qaly* or "adjusted life year*" or "quality-adjusted life year*" or "healthy years equivalent*").ti,ab,kf.

22	(daly* or "disability-adjusted life year*").ti,ab,kf.

23	daily life activity/

24	(adl or adls or "activit* of daily living*" or "activit* daily living*" or "daily living activit*" or "chronic limitation* of activit*").ti,ab,kf.

25	("health utilit*" or "health level*" or "health status" or "level of health").ti,ab,kf.

26	exp health status/

27	exp questionnaire/

28	exp economic evaluation/

29	(cost-effectiveness or "cost effectiveness" or "cost of illness" or "cost analysis" or "cost-benefit analysis" or "health care costs").ti,ab,kf.

30	(sf-36 or sf36 or "short-form 36" or "short form 36" or short-form-36 or "short form-36").ti,ab,kf.

31	("short form health survey-8" or sf-8 or "short-form health survey 8" or "short-form health survey-8" or "short form health survey 8" or "sf 8").ti,ab,kf.

32	(rand-36 or "rand 36 item-health survey" or rand36).ti,ab,kf.

33	("sickness impact profile" or sip or sip-68 or sip68).ti,ab,kf.

34	(euro-qol or eq-5d-5l or eq-5d-3l or eq-5d-y or eq-vas or "visual analogue scale" or vas).ti,ab,kf.

35	("chronic liver disease questionnaire" or cldq).ti,ab,kf.

36	("short form liver" or sf-ldqol or sfldqol).ti,ab,kf.

37	("functional assessment of cancer therapy" or fact-hep).ti,ab,kf.

38	("functional assessment of chronic illness therapy— palliative care" or facit-pal).ti,ab,kf.

39	("palliative care outcome scale" or ipos or pos-s or pos).ti,ab,kf.

40	("ascites questionnaire" or ascites-q or ascitesq).ti,ab,kf.

41	("ascites symptoms inventory" or asi-7 or asi7).ti,ab,kf.

42	("edmonton symptom assessment scale" or esas*).ti,ab,kf.

43	caregiver/ or caregiver burden/ or caregiver burnout/

44	caregiver*.ti,ab,kf.

45	("zarit burden interview" or zbi-12 or zbi12).ti,ab,kf.

46	("caregiver roles and responsibility scale" or crrs or "caregiver reaction assessment" or cra).ti,ab,kf.

47	("patient health questionnaire-9" or phq-9 or phq9 or hads-d or hads-a or hadsd or hadsa or hads or "hospital anxiety and depression scale").ti,ab,kf.

48	or/16-45

49	15 and 48

### COCHRANE

#1	MeSH descriptor: [Ascites] this term only

#2	(ascites):ti,ab,kw

#3	#1 or #2

#4	(refractory or severe or repeated or recurrent or diuretic-intolerant or "diuretic intolerant" or "diuretic resistant" or diuretic-resistant or persistent or untreatable or end-stage or "end stage"):ti,ab,kw

#5	#3 and #4

#6	MeSH descriptor: [Hypertension, Portal] explode all trees

#7	MeSH descriptor: [Hepatitis, Chronic] explode all trees

#8	MeSH descriptor: [Liver Cirrhosis] explode all trees

#9	(portal NEXT hypertens* or "cruveilhier baumgarten syndrome" or "cruveilhier-baumgarten syndrome"):ti,ab,kw

#10	(cirrhos* or cirrhot* or liver):ti,ab,kw

#11	(biliary NEXT cholang*):ti,ab,kw

#12	(chronic NEXT hepatit* or persistent NEXT hepatit* or "chronic active" NEXT hepatit*):ti,ab,kw

#13	("chronic delta" NEXT hepatit*):ti,ab,kw

#14	{OR #6-#13}

#15	#5 and #14

#16	MeSH descriptor: [Quality of Life] this term only

#17	("quality of life" or hrqol or qol or "life quality"):ti,ab,kw

#18	("patient reported" NEXT outcome* or "patient-reported" NEXT outcome*):ti,ab,kw

#19	MeSH descriptor: [Patient Reported Outcome Measures] this term only

#20	MeSH descriptor: [Quality-Adjusted Life Years] this term only

#21	(qualy* or qaly* or "adjusted life" NEXT year* or "quality-adjusted life" NEXT year* or "healthy years" NEXT equivalent*):ti,ab,kw

#22	(daly* or "disability-adjusted life" NEXT year*):ti,ab,kw

#23	MeSH descriptor: [Activities of Daily Living] this term only

#24	(ADL or ADLS or activit* NEXT "of daily living" or activit* NEXT "daily living" or "daily living" NEXT activit* or "chronic limitation of" NEXT activit*):ti,ab,kw

#25	("health" NEXT utilit* or "health" NEXT level* or "health status" or "level of health"):ti,ab,kw

#26	MeSH descriptor: [Health Status] this term only

#27	MeSH descriptor: [Surveys and Questionnaires] explode all trees

#28	MeSH descriptor: [Costs and Cost Analysis] explode all trees

#29	(cost-effectiveness or "cost effectiveness" or "cost of illness" or "cost analysis" or "cost-benefit analysis" or "health care costs"):ti,ab,kw

#30	(sf-36 or "short-form 36" or "short form liver" or "short form questionnaire" or "short form 36" or "short-form-36" or "short form-36"):ti,ab,kw

#31	("short form health survey-8" or sf-8 or "short-form health survey 8" or "short-form health survey-8" or "short form health survey 8" or "sf 8"):ti,ab,kw

#32	("Rand-36" or "Rand 36 item-health survey" or rand36):ti,ab,kw

#33	("Sickness impact profile" or "SIP" or "SIP-68" or sip68):ti,ab,kw

#34	(Euro-QOL or EQ-5D-5L or EQ-5D-3L or EQ-5D-Y or EQ-VAS or "Visual Analogue Scale" or VAS):ti,ab,kw

#35	("Chronic Liver Disease Questionnaire" or CLDQ):ti,ab,kw

#36	("Short form Liver" or SF-LDQOL or sfldqol):ti,ab,kw

#37	("Functional Assessment of Cancer Therapy" or FACT-Hep):ti,ab,kw

#38	("Functional Assessment of Chronic Illness Therapy— Palliative Care" or FACIT-Pal):ti,ab,kw

#39	("Palliative Care Outcome Scale" or IPOS or POS-S or POS):ti,ab,kw

#40	("Ascites questionnaire" or "Ascites-Q" or "ascitesq"):ti,ab,kw

#41	("Ascites Symptoms Inventory" or ASI-7 or asi7):ti,ab,kw

#42	("Edmonton symptom assessment scale" or ESAS*):ti,ab,kw

#43	MeSH descriptor: [Caregivers] explode all trees

#44	MeSH descriptor: [Caregiver Burden] this term only

#45	(caregiver*):ti,ab,kw

#46	("Zarit Burden Interview" or ZBI-12 or zbi12):ti,ab,kw

#47	("Caregiver Roles and Responsibility Scale" or CRRS or "Caregiver Reaction Assessment" or CRA):ti,ab,kw

#48	("Patient Health Questionnaire-9" or PHQ-9 or PHQ9 or HADS-D or HADS-A or HADSD or HADSA or HADS or "Hospital Anxiety and Depression Scale"):ti,ab,kw

#49	{OR #16-#48}

#50	#15 and #49

### PsycINFO

(((tiab(ascites) OR subject(ascites)) AND (tiab(refractory OR severe OR repeated OR recurrent OR diuretic-intolerant OR "diuretic intolerant" OR "diuretic resistant" OR diuretic-resistant OR persistent OR untreatable OR end-stage OR "end stage") OR subject(refractory OR severe OR repeated OR recurrent OR diuretic-intolerant OR "diuretic intolerant" OR "diuretic resistant" OR diuretic-resistant OR persistent OR untreatable OR end-stage OR "end stage"))) AND (MAINSUBJECT.EXACT.EXPLODE("Hepatitis") OR MAINSUBJECT.EXACT.EXPLODE("Cirrhosis (Liver)") OR (tiab(("portal hypertension" OR "portal hypertensive") OR "cruveilhier baumgarten syndrome" OR "cruveilhier-baumgarten syndrome") OR subject(("portal hypertension" OR "portal hypertensive") OR "cruveilhier baumgarten syndrome" OR "cruveilhier-baumgarten syndrome")) OR (tiab(cirrhos* OR cirrhot* OR liver) OR subject(cirrhos* OR cirrhot* OR liver)) OR (tiab("biliary cholang*") OR subject("biliary cholang*")) OR (tiab(("chronic hepatitis") OR "persistent hepatit*" OR "chronic active hepatit*") OR subject(("chronic hepatitis") OR "persistent hepatit*" OR "chronic active hepatit*")) OR (tiab("chronic delta hepatit*") OR subject("chronic delta hepatit*")))) AND ((MAINSUBJECT.EXACT.EXPLODE("Health Related Quality of Life") OR MAINSUBJECT.EXACT.EXPLODE("Quality of Life Measures") OR MAINSUBJECT.EXACT.EXPLODE("Quality of Life")) OR (tiab("quality of life" OR hrqol OR qol OR "life quality") OR subject("quality of life" OR hrqol OR qol OR "life quality")) OR (tiab("patient reported outcome*" OR "patient-reported outcome*") OR subject("patient reported outcome*" OR "patient-reported outcome*")) OR MAINSUBJECT.EXACT.EXPLODE("Patient Reported Outcome Measures") OR (tiab(qualy* OR qaly* OR "adjusted life year*" OR "quality-adjusted life year*" OR "healthy years equivalent*") OR subject(qualy* OR qaly* OR "adjusted life year*" OR "quality-adjusted life year*" OR "healthy years equivalent*")) OR (tiab(daly* OR "disability-adjusted life year*") OR subject(daly* OR "disability-adjusted life year*")) OR MAINSUBJECT.EXACT.EXPLODE("Activities of Daily Living") OR (tiab(ADL OR ADLS OR "activit* of daily living*" OR "activit* daily living*" OR "daily living activit*" OR "chronic limitation* of activit*") OR subject(ADL OR ADLS OR "activit* of daily living*" OR "activit* daily living*" OR "daily living activit*" OR "chronic limitation* of activit*")) OR (tiab("health utilit*" OR ("health level" OR "health levels") OR "health status" OR "level of health") OR subject("health utilit*" OR ("health level" OR "health levels") OR "health status" OR "level of health")) OR MAINSUBJECT.EXACT.EXPLODE("Health Status") OR ((MAINSUBJECT.EXACT.EXPLODE("General Health Questionnaire") OR MAINSUBJECT.EXACT.EXPLODE("Questionnaires")) OR MAINSUBJECT.EXACT.EXPLODE("Surveys")) OR MAINSUBJECT.EXACT.EXPLODE("Costs and Cost Analysis") OR (tiab("Cost-Effectiveness" OR "cost effectiveness" OR "cost of illness" OR "cost analysis" OR "cost-benefit analysis" OR "health care costs") OR subject("Cost-Effectiveness" OR "cost effectiveness" OR "cost of illness" OR "cost analysis" OR "cost-benefit analysis" OR "health care costs")) OR (tiab(sf-36 OR "short-form 36" OR "short form questionnaire" OR "short form 36" OR "short-form-36" OR "short form-36") OR subject(sf-36 OR "short-form 36" OR "short form questionnaire" OR "short form 36" OR "short-form-36" OR "short form-36")) OR (tiab("short form health survey-8" OR sf-8 OR "short-form health survey 8" OR "short-form health survey-8" OR "short form health survey 8" OR "sf 8") OR subject("short form health survey-8" OR sf-8 OR "short-form health survey 8" OR "short-form health survey-8" OR "short form health survey 8" OR "sf 8")) OR (tiab("Rand-36" OR "Rand 36 item-health survey" OR "RAND36") OR subject("Rand-36" OR "Rand 36 item-health survey" OR "RAND36")) OR (tiab("Sickness impact profile" OR "SIP" OR "SIP-68" OR "SIP68") OR subject("Sickness impact profile" OR "SIP" OR "SIP-68" OR "SIP68")) OR (tiab(Euro-QOL OR EQ-5D-5L OR EQ-5D-3L OR EQ-5D-Y OR EQ-VAS OR "Visual Analogue Scale" OR VAS) OR subject(Euro-QOL OR EQ-5D-5L OR EQ-5D-3L OR EQ-5D-Y OR EQ-VAS OR "Visual Analogue Scale" OR VAS)) OR (tiab("Chronic Liver Disease Questionnaire" OR CLDQ) OR subject("Chronic Liver Disease Questionnaire" OR CLDQ)) OR (tiab("Short form Liver Disease" OR SF-LDQOL OR sfldqol) OR subject("Short form Liver Disease" OR SF-LDQOL OR sfldqol)) OR (tiab("Functional Assessment of Cancer Therapy" OR FACT-Hep) OR subject("Functional Assessment of Cancer Therapy" OR FACT-Hep)) OR (tiab("Functional Assessment of Chronic Illness Therapy Palliative Care" OR FACIT-Pal) OR subject("Functional Assessment of Chronic Illness Therapy Palliative Care" OR FACIT-Pal)) OR (tiab("Palliative Care Outcome Scale" OR IPOS OR POS-S OR POS) OR subject("Palliative Care Outcome Scale" OR IPOS OR POS-S OR POS)) OR (tiab("Ascites questionnaire" OR "Ascites-Q" OR "ASCITESQ") OR subject("Ascites questionnaire" OR "Ascites-Q" OR "ASCITESQ")) OR (tiab("Ascites Symptoms Inventory" OR ASI-7 OR ASI7) OR subject("Ascites Symptoms Inventory" OR ASI-7 OR ASI7)) OR (tiab("Edmonton symptom assessment scale" OR ESAS*) OR subject("Edmonton symptom assessment scale" OR ESAS*)) OR (MAINSUBJECT.EXACT.EXPLODE("Caregiver Attitudes") OR MAINSUBJECT.EXACT.EXPLODE("Caregiver Burden") OR MAINSUBJECT.EXACT.EXPLODE("Caregivers")) OR (tiab("caregiver*") OR subject("caregiver*")) OR (tiab("Zarit Burden Interview" OR ZBI-12 OR ZBI12) OR subject("Zarit Burden Interview" OR ZBI-12 OR ZBI12)) OR (tiab("Caregiver Roles and Responsibility Scale" OR CRRS OR "Caregiver Reaction Assessment" OR CRA) OR subject("Caregiver Roles and Responsibility Scale" OR CRRS OR "Caregiver Reaction Assessment" OR CRA)) OR (tiab("Patient Health Questionnaire-9" OR PHQ-9 OR PHQ9 OR HADS-D OR HADS-A OR HADSD OR HADSA OR HADS OR "Hospital Anxiety and Depression Scale") OR subject("Patient Health Questionnaire-9" OR PHQ-9 OR PHQ9 OR HADS-D OR HADS-A OR HADSD OR HADSA OR HADS OR "Hospital Anxiety and Depression Scale")))

### Web of Science

1: TS=(ascites)

2: TS=(refractory or severe or repeated or recurrent or diuretic-intolerant or "diuretic intolerant" or "diuretic resistant" or diuretic-resistant or persistent or untreatable or end-stage or "end stage")

3: #1 AND #2

4: TS=("portal hypertens*" or "cruveilhier baumgarten syndrome" or "cruveilhier-baumgarten syndrome")

5: TS=(cirrhos* or cirrhot* or liver)

6: TS=("biliary cholang*")

7: TS=("chronic hepatit*" or "persistent hepatit*" or "chronic active hepatit*")

8: TS=("chronic delta hepatit*")

9: #4 OR #5 OR #6 OR #7 OR #8

10: #9 AND #3

11: TS=("quality of life" or hrqol or qol or "life quality")

12: TS=("patient reported outcome*" or "patient-reported outcome*")

13: TS=(qualy* or qaly* or "adjusted life year*" or "quality-adjusted life year*" or "healthy years equivalent*")

14: TS=(daly* or "disability-adjusted life year*")

15: TS=("health utilit*" or "health level*" or "health status" or "level of health")

16: TS=(cost-effectiveness or "cost effectiveness" or "cost of illness" or "cost analysis" or "cost-benefit analysis" or "health care costs")

17: TS=(sf-36 or "short-form 36" or "short form 36" or short-form-36 or "short form-36

18: TS=("short form health survey-8" or sf-8 or "short-form health survey 8" or short-form health survey-8 or "short form health survey 8" or "sf 8")

19: TS=(rand-36 or "rand 36 item-health survey" or rand36)

20: TS=("sickness impact profile" or sip or sip-68 or sip68)

21: TS=(euro-qol or eq-5d-5l or eq-5d-3l or eq-5d-y or eq-vas or "visual analogue scale" or vas)

22: TS=("chronic liver disease questionnaire" or "cldq")

23: TS=("short form liver" or sf-ldqol or sfldqol)

24: TS=("functional assessment of cancer therapy" or "fact-hep")

25: TS=("functional assessment of chronic illness therapy— palliative care" or facit-pal)

26: TS=("palliative care outcome scale" or ipos or pos-s or pos)

27: TS=("ascites questionnaire" or ascites-q or ascitesq)

28: TS=("ascites symptoms inventory" or asi-7 or asi7)

29: TS=("edmonton symptom assessment scale" or esas*)

30: TS=(caregiver*)

31: TS=("zarit burden interview" or zbi-12 or zbi12)

32: TS=("caregiver roles and responsibility scale" or crrs or "caregiver reaction assessment" or cra)

33: TS=("patient health questionnaire-9" or phq-9 or phq9 or hads-d or hads-a or hadsd or hadsa or hads or "hospital anxiety and depression scale")

34: #33 OR #32 OR #31 OR #30 OR #29 OR #28 OR #27 OR #26 OR #25 OR #24 OR #23 OR #22 OR #21 OR #20 OR #19 OR #18 OR #17 OR #16 OR #15 OR #14 OR #13 OR #12 OR #11

35: #34 AND #10

36: #34 AND #10

### CINAHL

S1: (MH "Ascites")

S2: TI Ascites OR AB Ascites OR TX Ascites

S3: S1 OR S2

S4: TI ( "Refractory" or "Severe" or "Repeated" or "Recurrent" or "diuretic-intolerant" or "diuretic intolerant" or "diuretic resistant" or "diuretic-resistant" or "persistent" or "untreatable" or "end-state" or "end stage" ) OR AB ( "Refractory" or "Severe" or "Repeated" or "Recurrent" or "diuretic-intolerant" or "diuretic intolerant" or "diuretic resistant" or "diuretic-resistant" or "persistent" or "untreatable" or "end-stage" or "end stage" ) OR TX ( "Refractory" or "Severe" or "Repeated" or "Recurrent" or "diuretic-intolerant" or "diuretic intolerant" or "diuretic resistant" or "diuretic-resistant" or "persistent" or "untreatable" or "end-stage" or "end stage" )

S5: S3 AND S4

S6: (MH "Hypertension, Portal+")

S7: (MH "Hepatitis, Chronic+")

S8: (MH "Liver Cirrhosis+")

S9: TI ( "portal hypertens*" or "cruveilhier baumgarten syndrome" or "cruveilhier-baumgarten syndrome" ) OR AB ( "portal hypertens*" or "cruveilhier baumgarten syndrome" or "cruveilhier-baumgarten syndrome" ) OR TX ( "portal hypertens*" or "cruveilhier baumgarten syndrome" or "cruveilhier-baumgarten syndrome" )

S10: TI ( cirrhos* or cirrhot* or liver) OR AB ( cirrhos* or cirrhot* or liver) OR TX ( cirrhos* or cirrhot* or liver)

S11: TI "biliary cholang*" OR AB "biliary cholang*" OR TX "biliary cholang*"

S12: TI ( ("chronic hepatit*" or "persistent hepatit*" or "chronic active hepatit*") ) OR AB ( ("chronic hepatit*" or "persistent hepatit*" or "chronic active hepatit*") ) OR TX ( ("chronic hepatit*" or "persistent hepatit*" or "chronic active hepatit*") )

S13: TI "chronic delta hepatit*" OR AB "chronic delta hepatit*" OR TX "chronic delta hepatit*"

S14: S6 OR S7 OR S8 OR S9 OR S10 OR S11 OR S12 OR S13

S15: S5 AND S14

S16: (MH "Quality of Life+")

S17: TI ( "quality of life" or hrqol or qol or "life quality" ) OR AB ( "quality of life" or hrqol or qol or "life quality" ) OR TX ( "quality of life" or hrqol or qol or "life quality" )

S18: TI ( "patient reported outcome*" or "patient-reported outcome*" ) OR AB ( "patient reported outcome*" or "patient-reported outcome*" ) OR TX ( "patient reported outcome*" or "patient-reported outcome*" )

S19: (MH "Patient-Reported Outcomes+")

S20: (MH "Quality-Adjusted Life Years")

S21: TI ( "qualy*" or "qaly*" or "adjusted life year*" or "quality-adjusted life year*" or "healthy years equivalent*" ) OR AB ( "qualy*" or "qaly*" or "adjusted life year*" or "quality-adjusted life year*" or "healthy years equivalent*" ) OR TX ( "qualy*" or "qaly*" or "adjusted life year*" or "quality-adjusted life year*" or "healthy years equivalent*" )

S22: TI ( "daly*" or "disability-adjusted life year*" ) OR AB ( "daly*" or "disability-adjusted life year*" ) OR TX ( "daly*" or "disability-adjusted life year*" )

S23: (MH "Activities of Daily Living+")

S24: TI ( "ADL" or "ADLS" or "activit* of daily living*" or "activit* daily living*" or "daily living activit*" or "chronic limitation* of activit*" ) OR AB ( "ADL" or "ADLS" or "activit* of daily living*" or "activit* daily living*" or "daily living activit*" or "chronic limitation* of activit*" ) OR TX ( "ADL" or "ADLS" or "activit* of daily living*" or "activit* daily living*" or "daily living activit*" or "chronic limitation* of activit*" )

S25: TI ( "health utilit*" or "health level*" or "health status" or "level of health" ) OR AB ( "health utilit*" or "health level*" or "health status" or "level of health" ) OR TX ( "health utilit*" or "health level*" or "health status" or "level of health" )

S26: (MH "Health Status+")

S27: (MH "Surveys+") OR (MH "Questionnaires+")

S28: (MH "Costs and Cost Analysis+")

S29: TI ( cost-effectiveness or "cost effectiveness" or "cost of illness" or "cost analysis" or "cost-benefit analysis" or "health care costs" ) OR AB ( cost-effectiveness or "cost effectiveness" or "cost of illness" or "cost analysis" or "cost-benefit analysis" or "health care costs") OR TX ( cost-effectiveness or "cost effectiveness" or "cost of illness" or "cost analysis" or "cost-benefit analysis" or "health care costs")

S30: TI ( "sf-36" or "short-form 36" or "short form 36" or "short-form-36" or "short form-36" ) OR AB ( "sf-36" or "short-form 36" or "short form 36" or "short-form-36" or "short form-36" ) OR TX ( "sf-36" or "short-form 36" or "short form 36" or "short-form-36" or "short form-36" )

S31: TI ( "short form health survey-8" or "sf-8" or "short-form health survey 8" or "short-form health survey-8" or "short form health survey 8" or "sf 8" ) OR AB ( "short form health survey-8" or "sf-8" or "short-form health survey 8" or "short-form health survey-8" or "short form health survey 8" or "sf 8" ) OR TX ( "short form health survey-8" or "sf-8" or "short-form health survey 8" or "short-form health survey-8" or "short form health survey 8" or "sf 8" )

S32: TI ( "Rand-36" or "Rand 36 item-health survey" OR "Rand36" ) OR AB ( "Rand-36" or "Rand 36 item-health survey" OR "Rand36") OR TX ( "Rand-36" or "Rand 36 item-health survey" OR "Rand36")

S33: TI ( "Sickness impact profile" or "SIP" or "SIP-68" OR "SIP68") OR AB ( "Sickness impact profile" or "SIP" or "SIP-68" OR "SIP68" ) OR TX ( "Sickness impact profile" or "SIP" or "SIP-68" OR "SIP68")

S34: TI ( "Euro-QOL" or "EQ-5D-5L" or "EQ-5D-3L" or "EQ-5D-Y" or "EQ-VAS" or "Visual Analogue Scale" or "VAS" ) OR AB ( "Euro-QOL" or "EQ-5D-5L" or "EQ-5D-3L" or "EQ-5D-Y" or "EQ-VAS" or "Visual Analogue Scale" or "VAS" ) OR TX ( "Euro-QOL" or "EQ-5D-5L" or "EQ-5D-3L" or "EQ-5D-Y" or "EQ-VAS" or "Visual Analogue Scale" or "VAS" )

S35: TI ( "Chronic Liver Disease Questionnaire" or "CLDQ" ) OR AB ( "Chronic Liver Disease Questionnaire" or "CLDQ" ) OR TX ( "Chronic Liver Disease Questionnaire" or "CLDQ" )

S36: TI ( "Short form Liver" or "SF-LDQOL" OR "SFLDQOL" ) OR AB ( "Short form Liver" or "SF-LDQOL" OR "SFLDQOL" ) OR TX ( "Short form Liver" or "SF-LDQOL" OR "SFLDQOL" )

S37: TI ( "Functional Assessment of Cancer Therapy" or "FACT-Hep" ) OR AB ( "Functional Assessment of Cancer Therapy" or "FACT-Hep" ) OR TX ( "Functional Assessment of Cancer Therapy" or "FACT-Hep" )

S38: TI ( "Functional Assessment of Chronic Illness Therapy—Palliative Care" or "FACIT-Pal" ) OR AB ( "Functional Assessment of Chronic Illness Therapy—Palliative Care" or "FACIT-Pal" ) OR TX ( "Functional Assessment of Chronic Illness Therapy—Palliative Care" or "FACIT-Pal" )

S39: TI ( "Palliative Care Outcome Scale" or "IPOS" or "POS-S" or "POS" ) OR AB ( "Palliative Care Outcome Scale" or "IPOS" or "POS-S" or "POS" ) OR TX ( "Palliative Care Outcome Scale" or "IPOS" or "POS-S" or "POS" )

S40: TI ( "Ascites questionnaire" or "Ascites-Q" or "ASCITESQ") OR AB ( "Ascites questionnaire" or "Ascites-Q" or "ASCITESQ" ) OR TX ( "Ascites questionnaire" or "Ascites-Q" or "ASCITESQ")

S41: TI ( "Ascites Symptoms Inventory" or "ASI-7" OR "ASI7") OR AB ( "Ascites Symptoms Inventory" or "ASI-7" OR "ASI7") OR TX ( "Ascites Symptoms Inventory" or "ASI-7" OR "ASI7")

S42: TI ( "Edmonton symptom assessment scale" or "ESAS*" ) OR AB ( "Edmonton symptom assessment scale" or "ESAS*" ) OR TX ( "Edmonton symptom assessment scale" or "ESAS*" )

S43: (MH "Caregivers") OR (MH "Caregiver Burden") OR (MH "Caregiver Attitudes") OR (MH "Caregiver Support") OR (MH "Caregiver Strain Index") OR (MH "Caregiver Support (Iowa NIC)") OR (MH "Caregiver Stressors (Iowa NOC)") OR (MH "Caregiver Role Strain (NANDA)") OR (MH "Risk for Caregiver Role Strain (NANDA)") OR (MH "Caregiver Role Strain (Saba CCC)")

S44: TI (caregiver*) OR AB (caregiver*) OR TX (caregiver*)

S45: TI ( "Zarit Burden Interview" or "ZBI-12" OR "ZBI12") OR AB ( "Zarit Burden Interview" or "ZBI-12" OR "ZBI12") OR TX ( "Zarit Burden Interview" or "ZBI-12" OR "ZBI12" )

S46: TI ( "Caregiver Roles and Responsibility Scale" or "CRRS" or "Caregiver Reaction Assessment" or "CRA" ) OR AB ( "Caregiver Roles and Responsibility Scale" or "CRRS" or "Caregiver Reaction Assessment" or "CRA" ) OR TX ( "Caregiver Roles and Responsibility Scale" or "CRRS" or "Caregiver Reaction Assessment" or "CRA" )

S47: TI ( "Patient Health Questionnaire-9" or "PHQ-9" or "PHQ9" or "HADS-D" or "HADS-A" or "HADSD" or "HADSA" or "HADS" or "Hospital Anxiety and Depression Scale" ) OR AB ( "Patient Health Questionnaire-9" or "PHQ-9" or "PHQ9" or "HADS-D" or "HADS-A" or "HADSD" or "HADSA" or "HADS" or "Hospital Anxiety and Depression Scale" ) OR TX ( "Patient Health Questionnaire-9" or "PHQ-9" or "PHQ9" or "HADS-D" or "HADS-A" or "HADSD" or "HADSA" or "HADS" or "Hospital Anxiety and Depression Scale" )

S48: S16 OR S17 OR S18 OR S19 OR S20 OR S21 OR S22 OR S23 OR S24 OR S25 OR S26 OR S27 OR S28 OR S29 OR S30 OR S31 OR S32 OR S33 OR S34 OR S35 OR S36 OR S37 OR S38 OR S39 OR S40 OR S41 OR S42 OR S43 OR S44 OR S45 OR S46 OR S47

S49: S15 AND S48

### **Appendix S3:** Reasons for the exclusion of potentially eligible studies

**Table S3.1. Reasons for exclusion of potentially eligible studies**

|  | **Reference** | **Reason for exclusion** |
| --- | --- | --- |
| 1 | Abrantes, C., Soares, E., Valério, P., Furtado, T., Vaz, A., & Assunçao, J. (2021). Use of Peritoneal Catheters for Patients Receiving Palliative Care. *Journal of Palliative Medicine*, *24*(1), 152–154. https://doi.org/10.1089/jpm.2019.0699 | Case report |
| 2 | Alavinejad, P., Hajiani, E., Danyaee, B., & Morvaridi, M. (2019). The effect of nutritional education and continuous monitoring on clinical symptoms, knowledge, and quality of life in patients with cirrhosis. *Gastroenterology & Hepatology from Bed to Bench*, *12*(1), 17–24. | Cohort included ascites but refractory ascites subgroup not identifiable |
| 3 | Albadry, M., Amer, M., Mostafa, W. A. E., Bayomi, H. A., El Shafie, A., & Alboraie, M. (2017). Stem cell transplantation improves survival, quality of life and synthetic functions of the liver in patients with end stage liver disease. *United European Gastroenterology Journal*, *5*(5), A395–A396. https://doi.org/10.1177/2050640617725676 | No patient-reported outcome measure used |
| 4 | Alhalel, N., Donlan, J., Scherrer, A. B., Bloom, J. A., Gaccione, P., Chung, R. T., El-Jawahri, A., & Ufere, N. (2019). Depression and anxiety symptoms in patient with end-stage liver disease and their caregivers. *Hepatology*, *70*, 454A. | Cohort included ascites but refractory ascites subgroup not identifiable |
| 5 | Alla, M., Shasthry, S., Mukund, A., & Sarin, S. (2023). Small frequent paracentesis using an indwelling catheter is safe and more effective as compared with repeated large volume paracentesis in cirrhotic patients with refractory ascites (I-CARE). https://doi.org/10.1097/HEP.0000000000000580 | No patient-reported outcome measure used |
| 6 | Anand, A., & Sharma, S. (2021). Sarcopenia Does Not Worsen Outcomes in Patients With Refractory Ascites Under Transjugular Intrahepatic Portosystemic Shunt Placement-Preliminary Evidence? *American Journal of Gastroenterology*, *116*(5), 1095–1096. https://doi.org/10.14309/ajg.0000000000001053 | Review |
| 7 | Anghelici, G., Pisarenko, S., Gaidau, M., Zugrav, T., Lupu, G., & Lisnic, L. (2022). Surgical mini-invasive correction of refractory ascites in patients with decompensated liver cirrhosis. *Surgical Endoscopy*, *36*(2), S515. https://doi.org/10.1007/s00464-022-09337-0 | No patient-reported outcome measure used |
| 8 | Anghelici, G. (2022). Surgical treatment of cirrhotic refractory ascites through cervical decompression of the thoracic lymphatic duct. *Surgical Endoscopy*, *36*(2), S514. https://doi.org/10.1007/s00464-022-09337-0 | No patient-reported outcome measure used |
| 9 | Anghelici, G., Pisarenco, S., Crudu, O., Zugrav, T., Lupu, G., & Roman, V. (2019). Complex mini-invasive surgical correction of refractory ascites in patients with liver cirrhosis. *Surgical Endoscopy*, *33*(2), S703. https://doi.org/10.1007/s00464-019-07109-x | No patient-reported outcome measure used |
| 10 | Arano, T., Tejima, K., Ishii, R., Hanaoka, Y., Fukumoto, T., Yamazaki, T., Murakawa, A., Mouri, D., Tashiro, J., Miwa, J., & Arai, M. (2016). Denver peritoneovenous shunt selectively enriches life of cirrhotic patients with refractory ascites. *Hepatology International*, *10*(1), S379. https://doi.org/10.1007/s12072-016-9707-8 | No patient-reported outcome measure used |
| 11 | Aspinall, R. (2018). Reducing recurrent hospital admissions in patients with decompensated cirrhosis. *British Journal of Hospital Medicine*, *79*(2), 93–96. https://doi.org/10.12968/hmed.2018.79.2.93 | Review |
| 12 | Ayllon, M. D., Ciria, R., Gomez-Luque, I., Rodriguez-Peralvarez, M., Padial, A., Luque, A., Lopez-Cillero, P., de la Mata, M., & Briceno, J. (2019). Use of Peritoneovenous Shunt for the Management of Refractory Ascites. *Transplantation proceedings*, *51*(1), 41–43. https://doi.org/10.1016/j.transproceed.2018.04.089 | Quantitative outcome data missing; no author response |
| 13 | Bakshi, N., & Singh, K. (2019). Nutrition status and its impact on quality of life and performance status in end-stage liver disease. *Indian Journal of Transplantation*, *13*(1), 31–37. https://doi.org/10.4103/ijot.ijot_39_18 | Cohort stratified by ascites grade only; refractory ascites not specified |
| 14 | Bakshi, N., & Singh, K. (2018). Impact of nutrition status on performance status and quality of life in end stage liver disease patients. *Hepatology International*, *12*(2), S428–S429. https://doi.org/10.1007/s12072-018-9852-3 | Cohort included ascites but refractory ascites subgroup not identifiable |
| 15 | Balbola, M., Eldin, A. M., El-Maadawy, G., Elsayaad, I., Salem, N., Hefney, A. E. H., & Hassan, R. (2011). Synthetic sapheno-peritoneal shunts in treatment of refractory ascites in egypt. *Trends in Medical Research*, *6*(3), 169–177. https://doi.org/10.3923/tmr.2011.169.177 | No patient-reported outcome measure used |
| 16 | Batisti, J., Diseases, D., & Haven, Y. N. (2022). Psychosocial assessment and physical symptoms: Correlation between symptoms and sipat score in liver transplant evaluation. *Hepatology*, *76*, S472–S473. https://doi.org/10.1002/hep.32697 | Cohort included ascites but refractory ascites subgroup not identifiable |
| 17 | Bañares, R., Albillos, A., Nakum, M., Gea, S., Varghese, A., & Green, W. (2023). An Economic Analysis of Transjugular Intrahepatic Portosystemic Covered Stent Shunt for Variceal Bleeding and Refractory Ascites in a Spanish Setting. *Advances in Therapy*, *40*(7), 3006–3020. https://doi.org/10.1007/s12325-023-02517-x | Cost-effectiveness analysis using utility values derived from external cohorts |
| 18 | Benmassaoud, A., Freeman, S., Roccarina, D., Torres, M., Sutton, A., Cooper, N., Prat, L., Cowlin, M., Milne, E., Hawkins, N., Davidson, B., Pavlov, C., Thorburn, D., Tsochatzis, E., & Gurusamy, K. (2020). Treatment for ascites in adults with decompensated liver cirrhosis: a network meta-analysis. *Cochrane Database of Systematic Reviews*. https://doi.org/10.1002/14651858.CD013123.pub2 | Meta-analysis |
| 19 | Bhanji, R., Carey, E., & Watt, K. (2017). Review article: maximising quality of life while aspiring for quantity of life in end-stage liver disease. *Alimentary Pharmacology & Therapeutics*, *46*(1), 16–25. https://doi.org/10.1111/apt.14078 | Review |
| 20 | Biro, B., Zakar, G., Foldine, Z., & Staudt, S. (2014). Longtermexperiences with peritoneal dialysis treatment of recractory edemas and ascites. *Nephrology Dialysis Transplantation*, *29*, iii474–iii475. https://doi.org/10.1093/ndt/gfu174 | Malignant ascites cohort |
| 21 | Brown, C., Kamal, A., & Muir, A. (2015). Delivering palliative care to patients with advanced liver diease: Creating collaborative models between GI and palliative medicine. *Journal of Pain and Symptom Management*, *49*(2), 378. | Review |
| 22 | Bureau, C., Adebayo, D., de Rieu, M., Elkrief, L., Valla, D., Peck-Radosavljevic, M., McCune, A., Vargas, V., Simon-Talero, M., Cordoba, J., Angeli, P., Rosi, S., MacDonald, S., Malago, M., Stepanova, M., Younossi, Z., Trepte, C., Watson, R., Borisenko, O., ... Jalan, R. (2017). Alfapump® system vs. large volume paracentesis for refractory ascites: A multicenter randomized controlled study. *Journal of Hepatology*, *67*(5), 940–949. https://doi.org/10.1016/j.jhep.2017.06.010 | Duplicate data |
| 23 | Caddick, K. J., Dhanda, A. D., Collins, P. L., Gordon, F. H., McCune, A. C., & Portal, A. J. (2010). Development of nurse led paracentesis service. *Gut*, *59*, A150–A151. https://doi.org/10.1136/gut.2009.209064c | No patient-reported outcome measure used |
| 24 | Caraceni, P., Riggio, O., Angeli, P., Alessandria, C., Neri, S., Foschi, F., Levantesi, F., Airoldi, A., Boccia, S., & Baroni, G. (2018). Long-term albumin administration in decompensated cirrhosis (ANSWER): An open-label randomised trial. *The Lancet*, *391*(10138), 2417–2429. https://doi.org/10.1016/S0140-6736(18)30840-7 | Cohort included ascites but refractory ascites subgroup not identifiable |
| 25 | Carol, M., Perez-Guasch, M., Sola, E., Cervera, M., Martinez, S., Juanola, A., Ma, A. T., Avitabile, E., Napoleone, L., Pose, E., Graupera, I., Honrubia, M., Korenjak, M., Torres, F., Gines, P., & Fabrellas, N. (2022). Stigmatization is common in patients with non-alcoholic fatty liver disease and correlates with quality of life. *PLOS One*, *17*(4), e0265153. https://doi.org/10.1371/journal.pone.0265153 | Cohort included ascites but refractory ascites subgroup not identifiable |
| 26 | Cassar, N., & Heaton, N. (2018). Use of peritoneovenous shunts in patients with intractable ascites in the era of liver transplantation. *HPB*, *20*, S786. https://doi.org/10.1016/j.hpb.2018.06.2376 | No patient-reported outcome measure used |
| 27 | Chatrath, H., Liangpunsakul, S., Ghabril, M., Otte, J., Chalasani, N., & Vuppalanchi, R. (2012). Prevalence and morbidity associated with muscle cramps in patients with cirrhosis. *The American Journal of Medicine*, *125*(10), 1019–1025. https://doi.org/10.1016/j.amjmed.2012.03.012 | Cohort included ascites but refractory ascites subgroup not identifiable |
| 28 | Chen, J., Liu, H., Yu, J., Chen, C., Shih, M., Liu, Y., & Hsieh, C. (2005). Modified saphenous-peritoneal shunt in refractory ascites: New technique. *Anz Journal of Surgery*, *75*(3), 128–131. https://doi.org/10.1111/j.1445-2197.2005.03315.x | No patient-reported outcome measure used |
| 29 | Chishina, H., Hagiwara, S., Nishida, N., Ueshima, K., Sakurai, T., Ida, H., Minami, Y., Takita, M., Kono, M., Minami, T., Iwanishi, M., Umehara, Y., Watanabe, T., Komeda, Y., Arizumi, T., & Kudo, M. (2016). Clinical Factors Predicting the Effect of Tolvaptan for Refractory Ascites in Patients with Decompensated Liver Cirrhosis. *Digestive Diseases*, *34*(6), 659–664. https://doi.org/10.1159/000448828 | No patient-reported outcome measure used |
| 30 | Christensen, L., Wildgaard, L., & Wildgaard, K. (2016). Permanent catheters for recurrent ascites-a critical and systematic review of study methodology. *Supportive Care in Cancer*, *24*(6), 2767–2779. https://doi.org/10.1007/s00520-016-3145-y | Review |
| 31 | Coimbra, E. (2015). TIPS for refractory ascites and variceal bleeding. *CardioVascular and Interventional Radiology*, *38*(3), S147–S148. https://doi.org/10.1007/s00270-015-1173-5 | Review |
| 32 | Congly, S. E., Shen, N. T., Lee, S. S., Abraldes, J. G., & Swain, M. G. (2019). Palliative management of refractory ascites-a cost utility analysis. *Hepatology*, *70*, 251A. | Cost-effectiveness analysis using utility values derived from external cohorts |
| 33 | CTRI/2020/11/028986. (2020). *Effect of albumin with Standard Treatment in Liver Cirrhosis (Liver Disease)* [Clinical trial registration]. Clinical Trials Registry-India. https://ctri.nic.in/Clinicaltrials/showallp.php?modid=1&compid=19&EncHid=CTRI/2020/11/028986 | Not published in full |
| 34 | Cumming-Browne Hefner, A. M. (2013). Nutritional Status of Patients with End Stage Liver Disease: An Outpatient Assessment. | Cohort included ascites but refractory ascites subgroup not identifiable |
| 35 | De, A., Kumari, S., Singh, A., Kaur, A., Sharma, R., Bhalla, A., Sharma, N., Kalra, N., & Singh, V. (2021). Multiple Cycles of Granulocyte Colony-Stimulating Factor Increase Survival Times of Patients With Decompensated Cirrhosis in a Randomized Trial. *Clinical Gastroenterology and Hepatology*, *19*(2), 375. https://doi.org/10.1016/j.cgh.2020.02.022 | Cohort stratified by ascites grade only; refractory ascites not specified |
| 36 | de Goede, B., van Rooijen, M., van Kempen, B., Polak, W., de Man, R., Taimr, P., Lange, J., Metselaar, H., & Kazemier, G. (2021). Conservative treatment versus elective repair of umbilical hernia in patients with liver cirrhosis and ascites: results of a randomized controlled trial (CRUCIAL trial). *Langenbecks Archives of Surgery*, *406*(1), 219–225. https://doi.org/10.1007/s00423-020-02033-4 | Cohort included ascites but refractory ascites subgroup not identifiable |
| 37 | De Waele, C., Nguyen-Khac, E., Di Martino, V., Paquette, B., Balssa, L., Thevenot, T., & Weil-Verhoeven, D. (2022). Place of the implantable alfapump device in the management of refractory ascites. *Hepato-Gastro et Oncologie Digestive*, *29*(1), 45–55. https://doi.org/10.1684/hpg.2021.2295 | Review |
| 38 | de Wit, K., Schaapman, J., Nevens, F., Verbeek, J., Coenen, S., Cuperus, F., Kramer, M., Tjwa, E., Mostafavi, N., Dijkgraaf, M., van Delden, O., Beuers, U., Coenraad, M., & Takkenberg, R. (2020). Prevention of hepatic encephalopathy by administration of rifaximin and lactulose in patients with liver cirrhosis undergoing placement of a transjugular intrahepatic portosystemic shunt (TIPS): a multicentre randomised, double blind, placebo controlled trial (PEARL trial). *BMJ Open Gastroenterology*, *7*(1). https://doi.org/10.1136/bmjgast-2020-000531 | Not published in full |
| 39 | Deltenre, P., Mathurin, P., Dharancy, S., Moreau, R., Bulois, P., Henrion, J., Pruvot, F., Ernst, O., Paris, J., & Lebrec, D. (2005). Transjugular intrahepatic portosystemic shunt in refractory ascites: a meta-analysis. *Liver International*, *25*(2), 349–356. https://doi.org/10.1111/j.1478-3231.2005.01095.x | Meta-analysis |
| 40 | Deng, L. X., Bischoff, K. E., Kent, D. S., O'Riordan, D. L., Pantilat, S. Z., & Lai, J. C. (2021). Frailty is strongly associated with self-reported symptom burden among patients with cirrhosis. *European Journal of Gastroenterology & Hepatology*, *34*, e395–e400. https://doi.org/10.1097/MEG.0000000000002113 | Cohort included ascites but refractory ascites subgroup not identifiable |
| 41 | Desai, A. P., Madathanapalli, A., Tang, Q., Orman, E. S., Lammert, C., Patidar, K. R., Nephew, L. D., Ghabril, M., Monahan, P. O., & Chalasani N. AO - Desai, A. P. O. H. O. O. A. (2023). PROMIS Profile-29 is a valid instrument with distinct advantages over legacy instruments for measuring the quality of life in chronic liver disease. *Hepatology*, *78*(6), 1788–1799. https://doi.org/10.1097/HEP.0000000000000480 | Cohort included ascites but refractory ascites subgroup not identifiable |
| 42 | Dong, L., & Chen, J. (2017). Study on sleep quality of cirrhotic patients and its influencing factors. *Chinese Nursing Research*, *31*(36), 4663–4666. https://doi.org/10.3969/j.issn.1009-6493.2017.36.020 | Full text not available |
| 43 | Donlan, J., Kaplan, A., Noll, A., Pintro, K., Horick, N., Zeng, C., Edelen, M., Soetan, Z., Comrie, C., Indriolo, T., Li, L., Zhu, E., Armstrong, M. E., Thompson, L. L., Zhou, J., Diop, M. S., Mason, N., Engel, K. G., Rowland, M., … Ufere, N. N. (2025). Prognostic communication, symptom burden, psychological distress, and quality of life among patients with decompensated cirrhosis. *Clinical Gastroenterology and Hepatology*, *23*(10), 1746–1754.e4. https://doi.org/10.1016/j.cgh.2024.10.017 | Refractory ascites subgroup data unavailable from authors |
| 44 | Elsabaawy, M., Assem, M., Badran, H., Mahmoud, A., Elsabaawy, D., & Ragab, A. (2024). Impacts of rifaximin and midodrine on morbidity, mortality, and quality of life in patients with decompensated liver cirrhosis. *European Journal of Gastroenterology & Hepatology*, *36*(8), 1022–1028. https://doi.org/10.1097/MEG.0000000000002797 | Cohort stratified by ascites grade only; refractory ascites not specified |
| 45 | Engelmann, C., Brosteanu, O., Tenckhoff, H., Babatz, J., Lammert, F., Manns, M., Schiefke, I., Bruns, T., Trautwein, C., & Zeuzem, S. (2015). Alfapump system versus transjugular intrahepatic portosystemic shunt and paracentesis in the treatment of ascites. A multicentre randomised controlled study (agua-trial). | Not published in full |
| 46 | Foster, C., Baki, J., Nikirk, S., Williams, S., Parikh, N., & Tapper, E. (2020). Comprehensive Health-State Utilities in Contemporary Patients With Cirrhosis. *Hepatology Communications*, *4*(6), 852–858. https://doi.org/10.1002/hep4.1512 | Cohort stratified by ascites grade only; refractory ascites not specified |
| 47 | Foster, M. C., Nikirk, S., Baki, J., Parikh, N. D., & Tapper, E. B. (2019). A prospective study to define the contemporary health state utilities for persons with cirrhosis. *Hepatology*, *70*, 42A–43A. | Cohort included ascites but refractory ascites subgroup not identifiable |
| 48 | Freeman, E., Majeed, A., Kemp, W., & Roberts, S. (2020). Long-term albumin infusions in the home are cost-effective for patients with decompensated cirrhosis. *Journal of Gastroenterology and Hepatology (Australia)*, *35*, 35–36. https://doi.org/10.1111/jgh.15269 | Review |
| 49 | Fukui, H., Kawaratani, H., Kaji, K., Takaya, H., & Yoshiji, H. (2018). Management of refractory cirrhotic ascites: challenges and solutions. *Hepatic Medicine-evidence and Research*, *10*, 55–71. https://doi.org/10.2147/HMER.S136578 | Review |
| 50 | Gantzel, R., Aagaard, N., Vilstrup, H., Watson, H., Gronbæk, H., & Jepsen, P. (2022). Development and validation of the Cirrhotic Ascites Severity model-A patient-reported outcome-based model to predict 1-year mortality. *Hepatology Communications*, *6*(11), 3175–3185. https://doi.org/10.1002/hep4.2065 | PROM reported only as a component of a composite score |
| 51 | Garbuzenko, D., & Arefyev, N. (2019). Current approaches to the management of patients with cirrhotic ascites. *World Journal of Gastroenterology*, *25*(28), 3738–3752. https://doi.org/10.3748/wjg.v25.i28.3738 | Review |
| 52 | Garcia, A., Moreira, R., Carol, M., Perez, M., Cervera, M., Nadal, R., Gratacos, J., Arslanow, A., Juanola, A., Soria, A., Pose, E., Graupera, I., Ginès, P., & Fabrellas, N. (2023). Refractory ascites in cirrhosis is associated with a major psychological burden in patients with cirrhosis and caregivers. *Journal of Hepatology*, *78*, S15–S16. | Refractory ascites subgroup data unavailable from authors |
| 53 | Gennings, C., Heuman, D., Fulton, O., & Sanyal, A. (2010). Use of desirability functions to evaluate health status in patients with cirrhosis. *Journal of Hepatology*, *52*(5), 665–671. https://doi.org/10.1016/j.jhep.2009.12.026 | No patient-reported outcome measure used |
| 54 | Ghabril, M., Jackson, M., Gotur, R., Weber, R., Orman, E., Vuppalanchi, R., & Chalasani, N. (2017). Most individuals with advanced cirrhosis have sleep disturbances, which are associated with poor quality of life. *Clinical Gastroenterology and Hepatology*, *15*(8), 1271–1278. https://doi.org/10.1016/j.cgh.2017.01.027 | Cohort included ascites but refractory ascites subgroup not identifiable |
| 55 | Goel, A., Tarlow, B. D., Kwong, A. J., Hu, K., Cheng, X., Kim, S. H., Charu, V., Dronamraju, D., Kim, W. R., & Kwo, P. Y. (2023). Empagliflozin in diuretic refractory ascites (DRAIN-EM): Results of a single-center feasibility study. *Hepatology*, *78*, S1337–S1338. https://doi.org/10.1097/HEP.0000000000000580 | No patient-reported outcome measure used |
| 56 | Gross, C., Malinchoc, M., Kim, W., Evans, R., Wiesner, R., Petz, J., Crippin, J., Klintmalm, G., Levy, M., Ricci, P., Therneau, T., & Dickson, E. (1999). Quality of life before and after liver transplantation for cholestatic liver disease. *Hepatology*, *29*(2), 356–364. https://doi.org/10.1002/hep.510290229 | Cohort included ascites but refractory ascites subgroup not identifiable |
| 57 | Grover, I., Singh, N., Gunjan, D., Sati, H. C., & Saraya, A. (2018). Assessment of quality of life in patients with cirrhosis. *Journal of Clinical and Experimental Hepatology*, *8*, S129. https://doi.org/10.1016/j.jceh.2018.06.511 | Cohort included ascites but refractory ascites subgroup not identifiable |
| 58 | Grunwald, D., Tapper, E. B., Jiang, Z. G., Ahmed, M., & Malik, R. (2016). A Standardized Assessment of Functional Disability Predicts 1-year Mortality in Patients Undergoing Transjugular Intrahepatic Portosystemic Shunt for Refractory Ascites. *Journal of Clinical Gastroenterology*, *50*(1), 75–79. https://doi.org/10.1097/MCG.0000000000000339 | No patient-reported outcome measure used |
| 59 | Haddad, L., Conte, T., Andraus, W., Soarez, P., & Carneiro, L. A. (2016). Cost-effectiveness analysis comparing tips with large volume paracentesis in cirrhotic patients with refractory ascites. *HPB*, *18*, e30. | Cost-effectiveness analysis using utility values derived from external cohorts |
| 60 | Hansen, L., Chang, M. F., Hiatt, S., Dieckmann, N. F., & Lee, C. S. (2024). Distinct Longitudinal Trajectories of Symptom Burden Predict Clinical Outcomes in End-Stage Liver Disease. *Clinical and Translational Gastroenterology*. https://doi.org/10.14309/ctg.0000000000000728 | Cohort included ascites but refractory ascites subgroup not identifiable |
| 61 | Hansen, L., Chang, M. F., Lee, C. S., Hiatt, S. O., Dieckmann, N. F., & Lyons, K. S. (2019). Mental and physical quality of life in end stage liver disease patients and caregivers-a dyadic analysis. *Hepatology*, *70*, 284A–285A. | Duplicate data |
| 62 | Huang, H., Chen, M., Hsieh, L., Chiu, H., Miao, N., Wu, C., & Lin, K. (2018). Validation of a Symptoms Distress Scale in a Cirrhotic Population Using Item Response Theory. *Nursing Research*, *67*(5), 359–368. https://doi.org/10.1097/NNR.0000000000000296 | Cohort included ascites but refractory ascites subgroup not identifiable |
| 63 | Hudson, B., McCune, A., Forbes, K., & Verne, J. (2017). The effect of planned care on resource use and end of life outcomes in patients with diuretic resistant ascites: A retrospective cohort study of deaths in England. *Journal of Hepatology*, *66*(1), S134. | No patient-reported outcome measure used |
| 64 | Iacob, S., Mina, V., Mandea, M., Iacob, R., Vadan, R., Boar, V., Ionescu, G., Buzescu, D., Gheorghe, C., & Gheorghe, L. (2022). Assessment of Sarcopenia Related Quality of Life Using SarQoL® Questionnaire in Patients With Liver Cirrhosis. *Frontiers in Nutrition*, *9*. https://doi.org/10.3389/fnut.2022.774044 | Refractory ascites subgroup data unavailable from authors |
| 65 | IRCT20230514058189N1. (2023). *Effects of flaxseed consumption in liver cirrhosis* [Clinical trial registration]. Iranian Registry of Clinical Trials. https://en.irct.ir/trial/IRCT20230514058189N1 | Not published in full |
| 66 | Itou, M., Kawaguchi, T., Taniguchi, E., Oku, Y., Fukushima, N., Ando, E., Oriishi, T., Uchida, Y., Otsuka, M., Tanaka, S., Iwasaki, S., Torii, M., Yoshida, K., Adachi, Y., Suga, M., Yoshiyama, M., Ibi, R., Akiyama, Y., Takakura, M., ... Sata, M. (2009). Branched-chain amino acid supplements reduced ascites and increased the quality of life in a patient with liver cirrhosis: A case report. *Molecular Medicine Reports*, *2*(6), 977–981. https://doi.org/10.3892/mmr_00000201 | Case report |
| 67 | Jalan, R. (2015). Management of severe ascites: Intraperitoneal alfapump versus TIPS. *Journal of Viral Hepatitis*, *22*, 13. https://doi.org/10.1111/jvh.12440 | Review |
| 68 | Janani, K., Varghese, J., Jain, M., Harika, K., Srinivasan, V., Michael, T., & Jayanthi, V. (2017). HRQOL using SF36 (generic specific) in liver cirrhosis. *Indian Journal of Gastroenterology*, *36*(4), 313–317. https://doi.org/10.1007/s12664-017-0773-1 | Author contact details unavailable |
| 69 | Janani, K., Jain, M., Vargese, J., Srinivasan, V., Harika, K., Michael, T., & Venkataraman, J. (2018). Health-related quality of life in liver cirrhosis patients using SF-36 and CLDQ questionnaires. *Clinical and Experimental Hepatology*, *4*(4), 232–239. https://doi.org/10.5114/ceh.2018.80124 | No response from authors to provide RA data |
| 70 | Jara, M., Bednarsch, J., Malinowski, M., Lüttgert, K., Orr, J., Puhl, G., Seehofer, D., Neuhaus, P., & Stockmann, M. (2014). Predictors of quality of life in patients evaluated for liver transplantation. *Clinical Transplantation*, *28*(12), 1331–1338. https://doi.org/10.1111/ctr.12426 | Cohort stratified by ascites grade only; refractory ascites not specified |
| 71 | Jensen, E. E., Baltzer, R., Bjerg, S. H., & Bager, P. (2018). Supportive care in end-stage liver disease: a clinical review. *Gastrointestinal Nursing*, *16*(6), 38–43. https://doi.org/10.12968/gasn.2018.16.6.38 | Review |
| 72 | Jeong, S. W. (2018). [Ascites]. *The Korean journal of gastroenterology = Taehan Sohwagi Hakhoe chi*, *72*(2), 49–55. https://doi.org/10.4166/kjg.2018.72.2.49 | Non-English language publication |
| 73 | Jepsen, P., Watson, H., Macdonald, S., Vilstrup, H., & Jalan, R. (2019). Development of a novel prognostic model for cirrhotic patients with difficult-to-treat ascites and low MELD scores. https://doi.org/10.1016/S0618-8278(19)31298-8 | Duplicate data |
| 74 | Kalaitzakis, E., Josefsson, A., Castedal, M., Henfridsson, P., Bengtsson, M., Andersson, B., & Björnsson, E. (2013). Gastrointestinal symptoms in patients with cirrhosis: A longitudinal study before and after liver transplantation. *Scandinavian Journal of Gastroenterology*, *48*(11), 1308–1316. https://doi.org/10.3109/00365521.2013.836755 | Cohort included ascites but refractory ascites subgroup not identifiable |
| 75 | Kamath, P. S., Neijenhuis, M., Gevers, T. J., Atwell, T., Drenth, J., & Gunneson, T. (2015). Validation of ascites-specific patient reported outcome questionnaires for patients with cirrhosis. *Hepatology*, *62*, 361A. https://doi.org/10.1002/hep.28209 | Duplicate data |
| 76 | Kanwal, F., Hays, R. D., Kilbourne, A. M., Dulai, G. S., & Gralnek, I. M. (2004). Are physician-derived disease severity indices associated with health-related quality of life in patients with end-stage liver disease? *American Journal of Gastroenterology*, *99*(9), 1726–1732. https://doi.org/10.1111/j.1572-0241.2004.30300.x | Cohort included ascites but refractory ascites subgroup not identifiable |
| 77 | Kawaratani, H., Moriya, K., Namisaki, T., Shimozato, N., Kaji, K., Takaya, H., Fujinaga, Y., Sawada, Y., Sato, S., Saikawa, S., Kubo, T., Akahane, T., Fukui, H., & Yoshiji, H. (2021). Ascites symptom inventory-7 is a valuable tool for evaluating the effectiveness of tolvaptan in patients with cirrhotic ascites. *Experimental and Therapeutic Medicine*, *21*(1). https://doi.org/10.3892/etm.2020.9462 | Cohort included ascites but refractory ascites subgroup not identifiable |
| 78 | Keimburg, S. A., Theysohn, J., Wetter, A., Buechter, M., Willuweit, K., Rashidi, J., Wedemeyer, H., & Markova, A. A. (2021). Improvement of quality of life after tips insertion. *Hepatology*, *74*, 1192A–1193A. https://doi.org/10.1002/hep.32188 | Author contact details unavailable |
| 79 | Kok, B., Whitlock, R., Ferguson, T., Bailey, R., Burak, K., Kowalczewski, J., Tangri, N., & Tandon, P. (2020). Health-Related Quality of Life: A Rapid Predictor of Hospitalization in Patients With Cirrhosis. *American Journal of Gastroenterology*, *115*(4), 575–583. https://doi.org/10.14309/ajg.0000000000000545 | Cohort included ascites but refractory ascites subgroup not identifiable |
| 80 | Koretz, R. L., Avenell, A., & Lipman, T. O. (2012). Nutritional support for liver disease. *Cochrane Database of Systematic Reviews*. | Review |
| 81 | Kozaki, K., IInuma, M., Takagi, T., Fukuda, T., Sanpei, T., Terunuma, Y., Yatabe, Y., & Akano, K. (2016). Cell-Free and Concentrated Ascites Reinfusion Therapy for Decompensated Liver Cirrhosis. *Therapeutic Apheresis and Dialysis*, *20*(4), 376–382. https://doi.org/10.1111/1744-9987.12469 | No patient-reported outcome measure used |
| 82 | Kwan, S. W., Allison, S. K., Gold, L. S., & Shin, D. S. (2018). Cost-Effectiveness of Transjugular Intrahepatic Portosystemic Shunt versus Large-Volume Paracentesis in Refractory Ascites: Results of a Markov Model Incorporating Individual Patient-Level Meta-Analysis and Nationally Representative Cost Data. *Journal of Vascular & Interventional Radiology*, *29*(11). https://doi.org/10.1016/j.jvir.2018.08.019 | Cost-effectiveness analysis using utility values derived from external cohorts |
| 83 | La Mura, V., & Salerno, F. (2016). Therapy of the refractory ascites: Total paracentesis vs. TIPS. *Gastroenterologia y hepatologia*, *39*(7), 477–80. https://doi.org/10.1016/j.gastrohep.2015.07.011 | Review |
| 84 | Larson, A. M. (2015). Palliative care for patients with end-stage liver disease. *Current gastroenterology reports*, *17*(5), 440. https://doi.org/10.1007/s11894-015-0440-6 | Review |
| 85 | Le, M., Patel, K., Paidpally, V., & Harvill, M. (2016). Refractory ascites: Battle of two shunts. *Journal of Vascular and Interventional Radiology*, *27*(3), S277. | Review |
| 86 | Les, I., Doval, E., Flavià, M., Jacas, C., Cárdenas, G., Esteban, R., Guardia, J., & Córdoba, J. (2010). Quality of life in cirrhosis is related to potentially treatable factors. *European Journal of Gastroenterology & Hepatology*, *22*(2), 221–227. https://doi.org/10.1097/MEG.0b013e3283319975 | Cohort included ascites but refractory ascites subgroup not identifiable |
| 87 | Macdonald, S., Jepsen, P., Watson, H., Vilstrup, H., & Jalan, R. (2018). The physical component of the SF-36 Quality of Life tool is an independent predictor of mortality in patients with severe ascites. *Journal of Hepatology*, *68*, S726–S727. | Duplicate data |
| 88 | Macken, L., Hashim, A., Mason, L., & Verma, S. (2019). Permanent indwelling peritoneal catheters for palliation of refractory ascites in end-stage liver disease: A systematic review. *Liver International*, *39*(9), 1594–1607. https://doi.org/10.1111/liv.14162 | Review |
| 89 | Macken, L., Corrigan, M., Prentice, W., Finlay, F., McDonagh, J., Rajoriya, N., Salmon, C., Donnelly, M., Evans, C., Ganai, B., Bedlington, J., Steer, S., Wright, M., Hudson, B., Verma, S., & British Assoc Study Liver, B. (2022). Palliative long-term abdominal drains for the management of refractory ascites due to cirrhosis: a consensus document. *Frontline Gastroenterology*, *13*, E116–E125. https://doi.org/10.1136/flgastro-2022-102128 | Review |
| 90 | Macken, L., Mason, L., Evans, C., Steer, S., Bremner, S., Crook, D., Thomson, S., Sheridan, D., Isaacs, P., & Wright, M. (2019). Reduce study: multi-centre feasibility rct in cirrhosis-related palliative refractory ascites. participant reported outcomes. https://doi.org/10.1136/gutjnl-2019-BSGAbstracts.229 | Duplicate data |
| 91 | Macken, L., Mason, L., Gage, H., Jordan, J., Touray, M., Evans, C., Austin, M., Parnell, N., Cooper, M., & Steer, S. (2019). Long-term palliative abdominal drains vs large-volume paracentesis in cirrhosis-related refractory ascites: multi-centre feasibility RCT (reduce). https://doi.org/10.1136/gutjnl-2019-BSGAbstracts.230 | Duplicate data |
| 92 | Macken, L., Mason, L., bremner, S., Gage, H., Touray, M., Evans, C., Cooper, M., Timeyin, J., Steer, S., & Lambert, D. (2019). Long term palliative abdominal drains versus large volume paracentesis in refractory ascites due to cirrhosis: a multi-centre feasibility randomised controlled trial (the REDUCe Study). https://doi.org/10.1016/S0618-8278(19)31318-0 | Duplicate data |
| 93 | Madoff, D., Cornman-Homonoff, J., Fortune, B., Gaba, R., Lipnik, A., Yarmohammadi, H., & Ray, C. (2021). Management of Refractory Ascites Due to Portal Hypertension: Current Status. *Radiology*, *298*(3), 493–504. https://doi.org/10.1148/radiol.2021201960 | Review |
| 94 | Marchesini, G., Bianchi, G., Amodio, P., Salerno, F., Merli, M., Panella, C., Loguercio, C., Apolone, G., Niero, M., & Abbiati, R. (2001). Factors associated with poor health-related quality of life of patients with cirrhosis. *Gastroenterology*, *120*(1), 170–178. https://doi.org/10.1053/gast.2001.21193 | Cohort included ascites but refractory ascites subgroup not identifiable |
| 95 | Marimuthu, K., Suresh Kumar, A., Sabanathan, S., Gowrishankar, A., Sasi Kumar, P., & Rajkumar, J. S. (2004). Indigenous cost-effective peritoneo-venous shunt for refractory ascites. *International Surgery*, *89*(2), 85–89. | Full text not available |
| 96 | Martin, L. (2012). Percutaneous Placement and Management of the Denver Shunt for Portal Hypertensive Ascites. *American Journal of Roentgenology*, *199*(4), W449–W453. https://doi.org/10.2214/AJR.12.9203 | Review |
| 97 | Mateos, B., Aicart-Ramos, M., Martin-Mateos, R., & Rodriguez-Gandia, M. A. (2016). Management of refractory ascites. *Medicine (Spain)*, *12*(12), 707–710. https://doi.org/10.1016/j.med.2016.05.026 | Non-English language publication |
| 98 | Mattock, R., Tripathi, D., O'Neill, F., Craig, J., Tanner, J., Patch, D., & Aithal, G. (2021). Economic evaluation of covered stents for transjugular intrahepatic portosystemic stent shunt in patients with variceal bleeding and refractory ascites secondary to cirrhosis. *BMJ Open Gastroenterology*, *8*(1). https://doi.org/10.1136/bmjgast-2021-000641 | Cost-effectiveness analysis using utility values derived from external cohorts |
| 99 | Mazumder, N., Ansari, S., Tapper, E., & Lok, A. (2023). Symptoms from refractory ascites: contributions from both abdominal pressure and the patient. *Journal of Hepatology*, *78*, 308–309. https://doi.org/10.1016/S0168-8278%2823%2900435-X | See published report below |
| 100 | Mazumder, N. R., Jezek, F., Ansari, S., Tapper, E. B., & Lok, A. S. (2024). The physiological determinants of symptom burden in cirrhosis with ascites. *United European gastroenterology journal*. https://doi.org/10.1002/ueg2.12675 | Cohort included ascites but not true refractory ascites |
| 101 | Mello, C. L. D., Conceição, T. M. A. D., Dal Pont, T., Peruzzolo, C. C., Lúcio, M. N., & Paulin, E. (2018). The Benefits of Neuromuscular Electrical Stimulation in the Muscular and Functional Capacity of Patients With Liver Cirrhosis: Protocol for a Randomized Clinical Trial. *Clinical Medicine Insights: Gastroenterology*, 1–1. https://doi.org/10.1177/1179552218811834 | Not published in full |
| 102 | Miyamoto, K., Kusumoto, C., & Kawabata, Y. (2006). The effectiveness of denver peritoneovenous shunt for the treatment of refractory ascites. *Japanese Journal of Gastroenterological Surgery*, *39*(3), 422–427. https://doi.org/10.5833/jjgs.39.422 | No patient-reported outcome measure used |
| 103 | Monsky, W. L., Yoneda, K. Y., MacMillan, J., Deutsch, L. S., Dong, P., Hourigan, H., Schwartz, Y., Magee, S., Duffield, C., Boak, T., & Cernilia, J. (2009). Peritoneal and Pleural Ports for Management of Refractory Ascites and Pleural Effusions: Assessment of Impact on Patient Quality of Life and Hospice/Home Nursing Care. *Journal of Palliative Medicine*, *12*(9), 811–817. https://doi.org/10.1089/jpm.2009.0061 | Published over 15 years prior; authors not contactable for subgroup data |
| 104 | Moscucci, F., Nardelli, S., Pentassuglio, I., Pasquale, C., Ridola, L., Merli, M., & Riggio, O. (2011). Previous overt hepatic encephalopathy rather than minimal hepatic encephalopathy impairs health-related quality of life in cirrhotic patients. *Liver International*, *31*(10), 1505–1510. https://doi.org/10.1111/j.1478-3231.2011.02598.x | Cohort included ascites but refractory ascites subgroup not identifiable |
| 105 | Nagel, M., Labenz, C., Wörns, M. A., Marquardt, J. U., Galle, P. R., Schattenberg, J. M., & Nguyen-Tat, M. (2020). Impact of acute-on-chronic liver failure and decompensated liver cirrhosis on psychosocial burden and quality of life of patients and their close relatives. *Health and Quality of Life Outcomes*, *18*, 7. https://doi.org/10.1186/s12955-019-1268-9 | Cohort included ascites but refractory ascites subgroup not identifiable |
| 106 | Nakum, M., Wiersma, T., & Di Stasi, F. (2022). A cost-effectiveness evaluation of the GORE VIATORR TIPS Endoprosthesis versus large volume paracentesis in the management of portal hypertension complications in the Spanish healthcare system. *Journal of Hepatology*, *77*, S617. https://doi.org/10.1016/S0168-8278%2822%2901552-5 | Cost-effectiveness analysis using utility values derived from external cohorts |
| 107 | Narahara, Y., Kanazawa, H., Osada, Y., Yoshimoto, H., Sakamoto, C., & Kobayashi, M. (2002). Long-term efficacy of transjugular intrahepatic portosystemic shunt for the treatment of refractory ascites. *Japanese Journal of Gastroenterology*, *99*(5), 474–482. | Non-English language publication |
| 108 | Nazarian, G. K., Ferral, H., Bjarnason, H., Castaneda-Zuniga, W. R., Rank, J. M., Bernadas, C. A., & Hunter, D. W. (1996). Effect of transjugular intrahepatic portosystemic shunt on quality of life. *American Journal of Roentgenology*, *167*(4), 963–969. https://doi.org/10.2214/ajr.167.4.8819395 | No patient-reported outcome measure used |
| 109 | NCT00222014. (2005). *TIPS With Coated Stents for Refractory Ascites in Patients With Cirrhosis* [Clinical trial registration]. ClinicalTrials.gov. https://clinicaltrials.gov/study/NCT00222014 | No patient-reported outcome measure used |
| 110 | NCT01528410. (2012). *ALFApump System Versus Standard of Care in Ascites Treatment* [Clinical trial registration]. ClinicalTrials.gov. https://clinicaltrials.gov/study/NCT01528410 | Duplicate data |
| 111 | NCT02975726. (2016). *Peritoneal Dialysis Catheters for the Treatment of Refractory Ascites* [Clinical trial registration]. ClinicalTrials.gov. https://clinicaltrials.gov/study/NCT02975726 | Not published in full |
| 112 | NCT03506893. (2018). *Medical-economic Evaluation of the Care of Refractory Ascites by Implantation of Alfapump® Device in Cirrhotic Patients* [Clinical trial registration]. ClinicalTrials.gov. https://clinicaltrials.gov/study/NCT03506893 | Not published in full |
| 113 | NCT04679571. (2020). *Efficacy of Targeted And Response-Guided Albumin Therapy Versus Standard Medical Treatment In Outcomes Of Recurrent Ascites In Patients With Decompensated Cirrhosis* [Clinical trial registration]. ClinicalTrials.gov. https://clinicaltrials.gov/study/NCT04679571 | No patient-reported outcome measure used |
| 114 | NCT05867602. (2023). *High Dose Albumin in Refractory Ascites* [Clinical trial registration]. ClinicalTrials.gov. https://clinicaltrials.gov/study/NCT05867602 | Not published in full |
| 115 | Neijenhuis, M., Gevers, T., Atwell, T., Tim, G., Kievit, W., Schimek, A., Drenth, J., & Kamath, P. S. (2017). Validation of patient-reported outcome measurements for symptom assessment in cirrhotic ascites. *Gastroenterology*, *152*(5), S1149. | Duplicate data |
| 116 | Neijenhuis, M. K., Gevers, T. J., Atwell, T. D., Gunneson, T. J., Drenth, J. P., & Kamath, P. S. (2015). Validation of ascites specific patient reported outcome measures in patients with cirrhosis. *United European Gastroenterology Journal*, *3*(5), A509. https://doi.org/10.1177/2050640615601623 | Duplicate data |
| 117 | Ngu, N., Anderson, P., Hunter, J., Figredo, A., Papaluca, T., Pianko, S., Dev, A., Bell, S., & Le, S. (2021). Short-term intraperitoneal catheters: An ambulatory care intervention for refractory ascites secondary to cirrhosis during COVID-19. *JGH Open*, *5*(10), 1154–1159. https://doi.org/10.1002/jgh3.12641 | Mixed cohort including malignant ascites; subgroup data unavailable from authors |
| 118 | Ngu, N., Saxby, E., Anderson, P., Stothers, L., Figredo, A., Worland, T., Pianko, S., Sievert, W., Rogers, B., & Bell, S. (2022). LivR Well: a feasibility study for a home-based, multidisciplinary liver optimization program for the first 28 days after an admission for acute-on-chronic liver failure. https://doi.org/10.1016/S0168-8278(22)01048-0 | Cohort included ascites but refractory ascites subgroup not identifiable |
| 119 | Ngu, N. L. Y., Anderson, P., Hunter, J., Figredo, A., Bell, S., Dev, A., Pianko, S., & Le, S. (2020). Long-term intraperitoneal catheters: A novel ambulatory care intervention for the management of refractory cirrhotic ascites during COVID-19. *Journal of Gastroenterology and Hepatology (Australia)*, *35*, 94. https://doi.org/10.1111/jgh.15269 | Duplicate data |
| 120 | NL-OMON29709. (2007). *TIPS with PTFE covered stents vs paracentesis + albumin infusion for the treatment of refractory ascites in patients with cirrhosis: a randomized trial comparing survival, quality of life and nutritional status* [Clinical trial registration]. Netherlands Trial Register. | Not published in full |
| 121 | Ogawa, Y., Naganuma, A., Hoshino, T., Hatanaka, T., Tanaka, H., Inagawa, M., Tanaka, T., Ogawa, T., Sato, K., & Kakizaki, S. (2016). Nutritional management at the end of life using the cell-free and concentrated ascites reinfusion therapy for patients with the refractory ascites. *Clinical Nutrition*, *35*, S18. | No patient-reported outcome measure used |
| 122 | Ohki, T., Sato, K., Yamada, T., Yamagami, M., Ito, D., Kojima, K., Arizumi, T., Seki, M., Toda, N., & Tagawa, K. (2015). The true efficacy of tolvaptan in patients with refractory ascites an experience of 60 cases compared to historical controls. *Hepatology International*, *9*(1), S97–S98. https://doi.org/10.1007/s12072-015-9609-1 | No patient-reported outcome measure used |
| 123 | Ohki, T., Hayata, Y., Karasawa, Y., Maeshima, S., Kawamura, S., Kojima, K., Seki, M., Toda, N., & Tagawa, K. (2016). The true efficacy of tolvaptan in patients with refractory ascites. *Hepatology International*, *10*(1), S90. https://doi.org/10.1007/s12072-016-9707-8 | No patient-reported outcome measure used |
| 124 | Ongaro, M., Ramer, L., Hassoun, J., Bastid, C., Magini, G., Goossens, N., & Spahr, L. (2021). Evolution of health-related quality of life in patients surviving an episode of alcoholic hepatitis. *Swiss Medical Weekly*, *151*, 23S. | Cohort included ascites but refractory ascites subgroup not identifiable |
| 125 | Orloff, M., Orloff, M., Orloff, S., & Girard, B. (1997). Experimental, clinical, and metabolic results of side-to-side portacaval shunt for intractable cirrhotic ascites. *Journal of the American College of Surgeons*, *184*(6), 557–570. | No patient-reported outcome measure used |
| 126 | Orman, E., Ghabril, M., Desai, A., Nephew, L., Patidar, K., Gao, S., Xu, C., & Chalasani, N. (2022). Patient-Reported Outcome Measures Modestly Enhance Prediction of Readmission in Patients with Cirrhosis. *Clinical Gastroenterology and Hepatology*, *20*(6), E1426–E1437. https://doi.org/10.1016/j.cgh.2021.07.032 | Cohort included ascites but refractory ascites subgroup not identifiable |
| 127 | Orr, J., Homer, T., Ternent, L., Newton, J., McNeil, C., Hudson, M., & Jones, D. (2014). Health related quality of life in people with advanced chronic liver disease. *Journal of Hepatology*, *61*(5), 1158–1165. https://doi.org/10.1016/j.jhep.2014.06.034 | Review |
| 128 | Orsi, F., Grasso, R., Bonomo, G., Monti, C., Marinucci, I., & Bellomi, M. (2002). Percutaneous peritoneovenous shunt positioning: technique and preliminary results. *European Radiology*, *12*(5), 1188–1192. https://doi.org/10.1007/s003300101049 | Qualitative study design |
| 129 | Ota, K. S., Schultz, N., & Segaline, N. A. (2021). Palliative Paracentesis in the Home Setting: A Case Series. *American Journal of Hospice & Palliative Medicine*, *38*(8), 1042–1045. https://doi.org/10.1177/1049909120963075 | No patient-reported outcome measure used |
| 130 | Pedersen, J., Bendtsen, F., & Moller, S. (2015). Management of cirrhotic ascites. *Therapeutic Advances in Chronic Disease*, *6*(3), 124–137. https://doi.org/10.1177/2040622315580069 | Review |
| 131 | Poonja, Z., Brisebois, A., van Zanten, S. V., Tandon, P., Meeberg, G., & Karvellas, C. J. (2014). Patients with cirrhosis and denied liver transplants rarely receive adequate palliative care or appropriate management. *Clinical Gastroenterology and Hepatology*, *12*(4), 692–698. https://doi.org/10.1016/j.cgh.2013.08.027 | Cohort included ascites but refractory ascites subgroup not identifiable |
| 132 | Pradhan, R., Bhandari, B., Pathak, R., Poudyal, S., Anees, S., Sharma, S., & Khadga, P. (2020). The Assessment of Health-Related Quality of Life in Patients With Chronic Liver Disease: A Single-Center Study. *Cureus Journal of Medical Science*, *12*(9). https://doi.org/10.7759/cureus.10727 | Cohort stratified by ascites grade only; refractory ascites not specified |
| 133 | Rajpurohit, S., Musunuri, B., Mohan, P., Bhat, G., & Shetty, S. (2023). Role of granulocyte colony stimulating factor in the treatment of cirrhosis of liver: a systematic review. *Journal of International Medical Research*, *51*(11). https://doi.org/10.1177/03000605231207064 | Review |
| 134 | Riedel, A., Kimer, N., Jensen, A., Dahl, E., Israelsen, M., Aamann, L., & Gluud, L. (2018). Development and predictive validity of the cirrhosis-associated ascites symptom scale: A cohort study of 103 patients. *World Journal of Gastroenterology*, *24*(15), 1650–1657. https://doi.org/10.3748/wjg.v24.i15.1650 | Cohort stratified by ascites grade only; refractory ascites not specified |
| 135 | Rubin, J. B., Loeb, R., Koch, B., Fenton, C., Huang, C. Y., & Lai, J. C. (2022). Pain is independently associated with mortality and healthcare utilization among patients with cirrhosis. *Hepatology*, *76*, S1098–S1099. https://doi.org/10.1002/hep.32697 | Cohort included ascites but refractory ascites subgroup not identifiable |
| 136 | Rudler, M., Mallet, M., Sultanik, P., Bouzbib, C., & Thabut, D. (2020). Optimal management of ascites. *Liver International*, *40*, 128–135. https://doi.org/10.1111/liv.14361 | Review |
| 137 | Russo, M. W. (2006). Quality of life after transjugular intrahepatic portal-systemic shunting (TIPS) for refractory ascites. *Evidence-Based Gastroenterology*, *7*(2), 57–58. https://doi.org/10.1097/01.ebg.0000211600.68175.73 | Full text not available |
| 138 | Saab, S., Ibrahim, A. B., Shpaner, A., Younossi, Z. M., Lee, C., Durazo, F., Han, S., Esrason, K., Wu, V., Hiatt, J., Farmer, D. G., Ghobrial, R. M., Holt, C., Yersiz, H., Goldstein, L. I., Tong, M. J., & Busuttil, R. W. (2005). MELD fails to measure quality of life in liver transplant candidates. *Liver transplantation : official publication of the American Association for the Study of Liver Diseases and the International Liver Transplantation Society*, *11*(2), 218–23. https://doi.org/10.1002/lt.20345 | Published over 15 years prior; authors not contactable for subgroup data |
| 139 | Sagara, M. K., Gangadharan, V., Sristhi, R., Shaji, B., Kellarai, A., & Joel, J. J. (2023). Assessment of health-related quality of life and its contributing factors using the WHOQOL-BREF questionnaire in patients with chronic liver disease. *Journal of Applied Pharmaceutical Science*, *13*(6), 246–255. https://doi.org/10.7324/JAPS.2023.120282 | Cohort stratified by ascites grade only; refractory ascites not specified |
| 140 | Salama, H., Zekri, A., Ahmed, R., Medhat, I., Abdallah, E., Darwish, T., Ahmed, O., & Bahnassy, A. (2012). Assessment of health-related quality of life in patients receiving stem cell therapy for end-stage liver disease: an Egyptian study. *Stem Cell Research & Therapy*, *3*. https://doi.org/10.1186/scrt140 | Cohort stratified by ascites grade only; refractory ascites not specified |
| 141 | Salem, H. A. A., Ismail, M. A., Salem, M. F., Moharram, M. M., & Ragheb, M. M. (2013). Health Related Quality of Life of Patients with Chronic Liver Disease Attending Family Practice Clinic, Suez Canal University Hospital, Ismailia, Egypt. *World Family Medicine*, *11*(2), 10–19. | Cohort included ascites but refractory ascites subgroup not identifiable |
| 142 | Samoylova, M. L., Covinsky, K. E., Haftek, M., Kuo, S., Roberts, J. P., & Lai, J. C. (2017). Disability in patients with end-stage liver disease: Results from the functional assessment in liver transplantation study. *Liver Transplantation*, *23*(3), 292–298. https://doi.org/10.1002/lt.24684 | Cohort included ascites but refractory ascites subgroup not identifiable |
| 143 | Sanyal, A., Genning, C., Reddy, K., Wong, F., Kowdley, K., Benner, K., McCashland, T., & NASTRA, G. (2003). The North American study for the treatment of refractory ascites. *Gastroenterology*, *124*(3), 634–641. https://doi.org/10.1053/gast.2003.50088 | Duplicate data |
| 144 | Senzolo, M., Cholongitas, E., Tibballs, J., Burroughs, A., & Patch, D. (2006). Transjugular intrahepatic portosystemic shunt in the management of ascites and hepatorenal syndrome. *European Journal of Gastroenterology & Hepatology*, *18*(11), 1143–1150. https://doi.org/10.1097/01.meg.0000236872.85903.3f | Review |
| 145 | Shamsaeefar, A., Nikeghbalian, S., Kazemi, K., Gholami, S., Sayadi, M., Azadian, F., Motazedian, N., & Malekhosseini, S. (2020). Quality of life among liver transplantation recipients before and after surgery: A single-center longitudinal study. *Indian Journal of Transplantation*, *14*(1), 48–52. https://doi.org/10.4103/ijot.ijot_42_19 | No ascites cohort |
| 146 | Shen, N., Schneider, Y., Congly, S., Rosenblatt, R., Namn, Y., Fortune, B., Jesudian, A., & Brown, R. (2018). Cost Effectiveness of Early Insertion of Transjugular Intrahepatic Portosystemic Shunts for Recurrent Ascites. *Clinical Gastroenterology and Hepatology*, *16*(9), 1503. https://doi.org/10.1016/j.cgh.2018.03.027 | Cost-effectiveness analysis using utility values derived from external cohorts |
| 147 | Shen, N. T., Schneider, Y., Jesudian, A., Fortune, B. E., Namn, Y., Rosenblatt, R. E., & Brown, R. S. (2017). Cost-effectiveness of transjugular intrahepatic portosystemic shunt (TIPS) insertion compared to serial large volume paracentesis (LVP) in recurrent ascites. *Hepatology*, *66*, 953A–954A. | Cost-effectiveness analysis using utility values derived from external cohorts |
| 148 | Shigeto, K., Kawaguchi, T., Niizeki, T., Kunitake, Y., Takedatsu, H., Tonan, T., Fujimoto, K., Tanaka, M., Abe, T., Naito, H., & Torimura, T. (2016). Efficacy of peritoneovenous shunt for treating tolvaptan-resistant refractory ascites in a cirrhotic patient with portal vein thrombosis: A case report. *Oncology Letters*, *11*(5), 3205–3209. https://doi.org/10.3892/ol.2016.4357 | Case report |
| 149 | Siemers, K. M., Stjernholm, J. D., & Pocha, C. (2023). Novel Innovations in Ascites Management. *South Dakota medicine : the journal of the South Dakota State Medical Association*, *76*(3), 116–119. | Review |
| 150 | Simon-Talero, M., Roccarina, D., Majumdar, A., Tsochatzis, E. A., Martinez, J., Picon, C., Albillos, A., Lampichler, K., Toth, D., Reiberger, T., Baiges, A., Darnell, A., Hernandez-Gea, V., Low, G., Abraldes, J. G., Tandon, P., Llop, E., Lopez, M., Calleja, J. L., ... Genesca, J. (2017). Portosystemic shunts in cirrhosis are associated to more complications and deteriorated quality of life. An international cohort study. *Digestive and Liver Disease*, *49*(1), e65. https://doi.org/10.1016/j.dld.2017.01.129 | Cohort included ascites but refractory ascites subgroup not identifiable |
| 151 | Singh, V., Verma, N., Kaur, A., Sharma, R., Bhalla, A., Sharma, N., Agarwal, R., Singh, A., Kumari, S., Taneja, S., & De, A. (2016). Quality of life and outcomes after multiple courses of granulocyte-colony stimulating factor and growth hormone in patients with decompensated cirrhosis. *Hepatology*, *64*(6), 1135A. | Cohort included ascites but refractory ascites subgroup not identifiable |
| 152 | Singh, J., Ebaid, M., & Saab, S. (2024). Advances in the management of complications from cirrhosis. *Gastroenterology Report*, *12*, goae072. https://doi.org/10.1093/gastro/goae072 | Review |
| 153 | Skladany, L., Koller, T., Vnencakova, J., & Skvarkova, B. (2020). Frailty assessed by the liver frailty index (LFI) predicts poor health-related quality of life (QOL) in patients hospitalised with liver cirrhosis. *Journal of Hepatology*, *73*, S735. https://doi.org/10.1016/S0168-8278%2820%2931921-8 | No author response to request for subgroup data |
| 154 | Solà, E., Watson, H., Graupera, I., Turón, F., Barreto, R., Rodríguez, E., Pavesi, M., Arroyo, V., Guevara, M., & Ginès, P. (2012). Factors related to quality of life in patients with cirrhosis and ascites: Relevance of serum sodium concentration and leg edema. *Journal of Hepatology*, *57*(6), 1199–1206. https://doi.org/10.1016/j.jhep.2012.07.020 | Cohort stratified by ascites grade only; refractory ascites not specified |
| 155 | Stotts, M., Cheung, A., Hammami, M., Westrich, D., Anderson, E., Counts, L., Befeler, A., Di Bisceglie, A., & Prather, C. (2021). Evaluation of Serum-Derived Bovine Immunoglobulin Protein Isolate in Subjects With Decompensated Cirrhosis With Ascites. *Cureus Journal of Medical Science*, *13*(6). https://doi.org/10.7759/cureus.15403 | Cohort included ascites but refractory ascites subgroup not identifiable |
| 156 | Tapper, E., Nikirk, S., Baki, J., & Lok, A. (2020). The SALTYFOOD trial: a randomized, controlled trial of home-delivered low-sodium meals for the management of refractory ascites. https://doi.org/10.1016/S0168-8278(20)31879-1 | Cohort included ascites but refractory ascites subgroup not identifiable |
| 157 | UMIN000026426. (2017). *A Study on predictors of treatment response concerning of the efficacy of tolvaptan on refractory ascites* [Clinical trial registration]. UMIN Clinical Trials Registry. https://upload.umin.ac.jp/cgi-open-bin/ctr_e/ctr_view.cgi?recptno=UMIN000026426 | Not published in full |
| 158 | Varma, R. K., Singh, K. B., Bready, E., Singh, D., Caridi, T. M., & Shoreibah M. AO - Varma, R. K. O. H. O. O. (2022). LVP vs TIPS for Refractory Ascites: Where Do We Stand? *Digestive Disease Interventions*, *6*(2), 86–93. https://doi.org/10.1055/s-0042-1745863 | Review |
| 159 | Verma, S., Hingwala, J., Low, J., Patel, A., Verma, M., Bremner, S., Haddadin, Y., Shinall, M., Komenda, P., & Ufere, N. (2023). Palliative clinical trials in advanced chronic liver disease: Challenges and opportunities. *Journal of Hepatology*, *79*(5), 1236–1253. https://doi.org/10.1016/j.jhep.2023.06.018 | Review |
| 160 | Vizsy, L., Beznicza, H., & Batorfi, J. (2005). Our experiences with saphenoperitoneal shunts in the treatment of intractable ascites. *Hepato-gastroenterology*, *52*(63), 920–922. | No patient-reported outcome measure used |
| 161 | Vizzutti, F., Schepis, F., Arena, U., Fanelli, F., Gitto, S., Aspite, S., Turco, L., Dragoni, G., Laffi, G., & Marra, F. (2020). Transjugular intrahepatic portosystemic shunt (TIPS): current indications and strategies to improve the outcomes. *Internal & Emergency Medicine*, *15*(1), 37–48. https://doi.org/10.1007/s11739-019-02252-8 | Review |
| 162 | Wang, M., Huang, C. Y., Cullaro, G., Covinsky, K., & Lai, J. C. (2023). Women with cirrhosis have lower self-rated health than men. *Hepatology communications*, *7*(3), e0065. https://doi.org/10.1097/HC9.0000000000000065 | Refractory ascites not clearly defined |
| 163 | Wedd, J. P., Noreika, D., Hashmi, I., Taylor, S., & Sterling, R. K. (2023). Symptom burden, quality of life, and palliative care in end-stage liver disease. *Hepatology*, *78*, S1774–S1775. https://doi.org/10.1097/HEP.0000000000000580 | Cohort included ascites but refractory ascites subgroup not identifiable |
| 164 | Weg, R., Shen, N., Maw, A., Jesudian, A., Crawford, C. V., & Pain, K. (2016). The use of indwelling peritoneal drainage devices in patients with refractory ascites due to end-stage liver disease: A systematic review. *American Journal of Gastroenterology*, *111*, S1281. https://doi.org/10.1038/ajg.2016.387 | Meta-analysis |
| 165 | Weil-Verhoeven, D., Di Martino, V., Stirnimann, G., Cervoni, J., Nguyen-Khac, E., & Thévenot, T. (2022). Alfapump® implantable device in management of refractory ascites: An update. *World Journal of Hepatology*, *14*(7), 1344–1356. https://doi.org/10.4254/wjh.v14.i7.1344 | Review |
| 166 | Williams, F. R., Milliken, D., Lai, J. C., & Armstrong M.J. AO - Williams, F. R. O. H. O. O. A. (2021). Assessment of the Frail Patient With End-Stage Liver Disease: A Practical Overview of Sarcopenia, Physical Function, and Disability. *Hepatology Communications*, *5*(6), 923–937. https://doi.org/10.1002/hep4.1688 | Review |
| 167 | Wong, F. (2023). Management of refractory ascites. *Clinical and Molecular Hepatology*, *29*(1), 16–32. https://doi.org/10.3350/cmh.2022.0104 | Review |
| 168 | Wong, F. (2023). Innovative approaches to the management of ascites in cirrhosis. *JHEP Reports*, *5*(7). https://doi.org/10.1016/j.jhepr.2023.100749 | Review |
| 169 | Wong, F., Vargas, H., Reddy, K., Pagadala, M., Rockey, D., Cheung, A., Devuni, D., Wilder, J., Saeian, K., Leventhal, T., Asrani, S., Pocha, C., Sundaram, V., Bajaj, J., Capel, J., & Kamath, P. (2022). An automatic low flow ascites pump improves ascites control and quality of life in patients with cirrhosis and recurrent ascites. *Hepatology*, *76*, S1155–S1156. | Duplicate data |
| 170 | Wong, F., Rajender Reddy, K., Tandon, P., Lai, J., Garcia-Tsao, G., O'Leary, J., Biggins, S. W., Vargas, H., Thacker, L., & Bajaj, J. S. (2023). Gender differences in the patient-reported outcomes and perception of ascites burden amongst outpatients with decompensated cirrhosis and ascites. *Journal of Hepatology*, *78*, 256. https://doi.org/10.1016/S0168-8278%2823%2900435-X | Refractory ascites subgroup data unavailable from authors |
| 171 | Wong, F., Bendel, E., Imaging, M., Shaw, C., Frederick, R. T., Haskal, Z., Sanyal, A. J., Asrani, S. K., Capel, J., & Kamath, P. S. (2018). Long-term follow-up of patients with cirrhosis and recurrent ascites treated with an automatic low flow ascites pump (alfapump) in North America. *Hepatology*, *68*, 561A. https://doi.org/10.1002/hep.30257 | Duplicate data |
| 172 | Wong, F., Bendel, E. C., Shaw, C. J., Frederick, R. T., Haskal, Z. J., Sanyal, A. J., Asrani, S. K., Capel, J., & Kamath, P. S. (2017). Improvement in qol and reduction in lvp requirement from the mosaic study: A multicenter, open-label, prospective 3-month study of the alfapump system in refractory ascites. *Hepatology*, *66*, 954A–955A. | Duplicate data |
| 173 | Woodland, H., Hudson, B., Forbes, K., McCune, A., Wright, M., & Liver BASL End Life Special, I. (2020). Palliative care in liver disease: what does good look like? *Frontline Gastroenterology*, *11*(3), 218–227. https://doi.org/10.1136/flgastro-2019-101180 | Review |
| 174 | Wu, X., Rabei, R., Keller, E., King, B., Kothary, N., Kohi, M., Taylor, A., & Heller, M. (2022). Tunneled Peritoneal Catheter vs Repeated Paracenteses for Recurrent Ascites: A Cost-Effectiveness Analysis. *Cardiovascular and Interventional Radiology*, *45*(7), 972–982. https://doi.org/10.1007/s00270-022-03103-4 | Cost-effectiveness analysis using utility values derived from external cohorts |
| 175 | Yan, L., Xie, F., Lu, J., Ni, Q., Shi, C., Tang, C., & Yang, J. (2015). The treatment of vasopressin V2-receptor antagonists in cirrhosis patients with ascites: a meta-analysis of randomized controlled trials. *BMC Gastroenterology*, *15*(1), 65–65. https://doi.org/10.1186/s12876-015-0297-z | No patient-reported outcome measure used |
| 176 | Yokoyama, K., Fukuda, H., Yamauchi, R., Higashi, M., Miyayama, T., Higashi, T., Uchida, Y., Shibata, K., Tsuchiya, N., Fukunaga, A., Umeda, K., Takata, K., Tanaka, T., Shakado, S., Sakisaka, S., & Hirai, F. (2022). Long-Term Effects of Rifaximin on Patients with Hepatic Encephalopathy: Its Possible Effects on the Improvement in the Blood Ammonia Concentration Levels, Hepatic Spare Ability and Refractory Ascites. *Medicina-lithuania*, *58*(9). https://doi.org/10.3390/medicina58091276 | No patient-reported outcome measure used |
| 177 | Younossi, Z. M., Stepanova, M., Nader, F., Hunt, S., Bureau, C., Inhaber, N., & Jalan, R. (2016). Patients with refractory ascites treated with alfapump system (AP) have better health-related quality of life (HRQL) as compared to those treated with large volume paracentesis (LVP): Results of a multicenter randomized controlled study. *Hepatology*, *64*(1), 1033A. | Duplicate data |
| 178 | Zama, I. N., & Edgar, M. (2012). Management of Symptomatic Ascites in Hospice Patients With Paracentesis: A Case Series Report. *American Journal of Hospice & Palliative Medicine*, *29*(5), 405–408. https://doi.org/10.1177/1049909111420130 | No patient-reported outcome measure used |
| 179 | Zhao, R., Lu, J., Shi, Y., Zhao, H., Xu, K., & Sheng, J. (2018). Current management of refractory ascites in patients with cirrhosis. *Journal of International Medical Research*, *46*(3), 1138–1145. https://doi.org/10.1177/0300060517735231 | Review |
| 180 | Zhuang, Z., Teng, G., Jeffery, R., Gemery, J., d'Othee, B., & Bettmann, M. (2002). Long-term results and quality of life in patients treated with transjugular intrahepatic portosystemic shunts. *American Journal of Roentgenology*, *179*(6), 1597–1603. https://doi.org/10.2214/ajr.179.6.1791597 | Published over 15 years prior; authors not contactable for subgroup data |

### **Appendix S4:** Details on the PRO tools used in eligible studies

**Table S4.1. PRO measures used in eligible studies**

| **Name and recall period** | **Purpose and structure** | **Scoring interpretation** | **Strengths** | **Limitations** | **MCID** |
| --- | --- | --- | --- | --- | --- |
| Chronic Liver Disease Questionnaire  – past 2 weeks | Disease-specific HRQL  29 items/six domains:  abdominal symptoms, fatigue, systemic symptoms, activity, emotional function, worry | Higher scores represent better HRQL  Items scored on a 1–7 Likert scale  Domain is the average score of the relevant items (rang 1–7)  Total score is the average of all domains | Widely used, validated in cirrhosis and is disease specific  Translated to 99 languages  Simple scoring system and interpretation | Low proportion of patients with advanced disease in original validation study  Aetiology-specific versions (CLDQ-HCV, CLDQ-MASH, CLDQ-PBC) limit cross‑disease comparison | Individual and total domains  ≈ 1 point  (range 0.3–0.8)^1^ |
| Minnesota Living with Heart Failure Questionnaire  – past 4 weeks | Disease-specific HRQL  Heart failure impact on physical and emotional aspects of life  21 items/three domains | Higher scores indicate worse HRQL  Each item is scored (0–5) and the sum total ranges from 0–105 | Available in over 30 languages  New version incorporates socioeconomic domain | Specific for patients with heart failure  Not validated for cirrhosis | Reported as 5 points for total score (in heart failure population)^2^ |
| Short-Form 36  – past 4 weeks | Generic HRQL  36 items/eight domains –  physical functioning, role limitations (physical and emotional), social functioning, mental health, vitality, bodily pain and general health | Higher scores indicating better HRQL  Domains scored 0–100  PCS and MCS calculated using norm-based scoring (population mean anchored at 50 with standard deviation being 10) | Generic tool allowing cross disease comparison  Widely used and available in multiple languages | Latest version requires license to use and interpret  PCS/MCS norm-based scoring is population  MCID can vary across conditions | 7–11 for most domains  except role limitations (range 15–18)  Component scores ≈ 3 points (range 3.0–3.2)^1^ |
| Short Form Liver Disease Quality of Life  – past 4 weeks | HRQL using 72-items  Incorporates the SF-36 (see above) alongside 36 liver-specific items  9 scales: effect of liver disease, memory/concentration, sexual functioning, quality of social interaction, health distress, sleep, loneliness, hopelessness and stigma | Higher scores indicate a better quality of life  Each domain calculated separately | Combines generic and disease-specific elements  Scores have been shown to predict mortality  White space section allows patients to specify concerns not covered by the tool | Available in limited languages and translation is a lengthy process  Length of tool can lead to cognitive fatigue | Estimated as 5.1 in validation paper however sample was too small for it to be accurate^3^ |
| Euro-Qol  – on the day | HRQL  5 domains: mobility, self-care, usual activity, pain/discomfort and anxiety/depression alongside a 20-cm VAS for self-rated health status | Generates a 5-digit health state profile  This is converted to a HUI using different value sets | Widely available in multiple languages with 45 different value sets  The VAS embedded in the tool provides a ‘sense check’ for the validity of the calculated HUI  HUI used in QALY calculations | Misses out on key health-related quality of life dimensions  Focuses on negative aspects of health (with potential ceiling effects for those experiencing improved well-being) | NA for ACLD |
| Short-Form 6-Dimension  – past 4 weeks | 6 HRQL dimensions from the SF-36 (see above) | Responses to generate a single health utility index | HUI used in QALY calculations  Multiple value sets available for HUI calculation | Requires license for its use  Preference weights used to calculate health utility index are population specific | NA for ACLD |
| Ascites-Questionnaire  – past 7 days | Symptom load of ascites  11 symptoms, each assessed on both frequency and severity | Higher scores represent worse symptom burden (range 11–100)  Total calculated by adding the Likert scale of frequency (0–5) and severity (1–5) for each symptom | Validated in RA  Simple scoring calculation | Available in a limited number of languages | 21 in total score^4^ |
| Ascites Symptom Inventory  – on the day | Symptom load of ascites using a 5-point question for each  Covers seven domains | Higher scores represent worse symptom load  Each question scores of 0–4 on a Likert scale  A total calculated by adding all seven scores with a range of 0-28 | Ascites specific score  Brief  Simple scoring calculation | Available in limited languages  Focus on physical impact of ascites and neglects symptoms such as fatigue, nausea, sleep disturbance  Potential for floor effect due to short recall period | 32 in total score^4^ |
| Cirrhosis Ascites Symptoms scale  – past 4 weeks | Symptom load of ascites using 14-items (rated 0–3) | Total score is the sum of 14 items  giving a range of 0–42 | Brief  Simple scoring calculation | Limited language availability (Danish)  Small validation cohort | NA for ACLD |
| Functional Assessment of Chronic Illness Therapy - Ascites Index  – past 7 days | Symptoms load ascites covering 13 items | Lower scores indicate worse symptom load  Each question given a score of 0–4 on a Likert scale  Total range 0–52 | Brief questionnaire with straightforward scoring system  Considers non-physical aspects of symptoms including emotional toll, fatigue and effect of diuretics  Total can be calculated despite missing items | Available in limited languages  Validated in ascites second to ovarian cancer therefore cohort was exclusively female | 14 in total score^4^ |
| Integrated Palliative care Outcome Scale  – past 3 days or 1 week | Assesses unmet palliative care needs  17 items/three sub-scales:  physical symptoms, emotional symptoms, communication/practical issues | Total score is calculated from the sum of the three subscales and ranges from 0–68  Higher scores indicate greater unmet needs and symptom burden | Comprehensive tool and available in many languages  Covers wide range of aspects of care including family anxieties, practical concerns, and information needs  White space section allows patients to specify concerns not covered by the tool | Limited validation studies  Difficult to integrate free space text answers into the score | NA for ACLD |
| Zarit Burden Interview  (12-items)  – no specified recall period | Caregiver burden  Shortened version of ZBI‑22 (22 items) that assesses subjective caregiver burden  Each scored on 5‑point Likert scale | Higher scores reflect higher caregiver burden  Total score calculated using the sum of the 12 questions (each scoring 0–4) to give a range of 0–48 | Quicker to complete than the full version  Can be used a screening tool for high caregiver burden | Overlooks the multidimensional construct of caregiver burden with one total score  No clearly defined MCID  Might be less responsive to the longer ZBI-22 questionnaire | NA for ACLD |
| Mishel Uncertainty in Illness Scale -Family Member/Adult  – on the day | Perceived illness-related uncertainty amongst caregivers/patients  Covers dimensions relating to disease information provided and unpredictability of disease trajectory  Items range 31–33 each on a 5‑point scale | Total score is the sum of all items (some with reverse scoring)  Total range (31–165)  Higher scores indicating a higher degree of uncertainty | Unique tool offering a comprehensive assessment of disease uncertainty  Validated in multiple cohorts and disease conditions | Available in limited number of languages  Length of tool can lead to cognitive fatigue (however shortened versions available) | NA for ACLD |
| Mutuality Scale | Assess relationship quality between patients and caregivers  15-item questionnaire/4 dimensions:  Love and affection, shared pleasurable activities, shared values and reciprocity | Items assessed on a 1–5 Likert scale  Total score ranges from 0 to a 100 with a higher score indicating better mutuality | Allows for dyadic analysis, i.e. the strength of the relationship from both the patient and caregiver’s perspective  Validated in multiple disease areas and some translations available | Some aspects dimensions (e.g. shared pleasurable activities) might not be applicable in terminal disease | NA for ACLD |

**Notes:** While there is no standard methodology for determining the Minimal Clinically Important Difference (MCID) of a tool’s score, earlier work has suggested that MCID may be considered as a change in score of 5% or greater.^5,6^ The MCIDs listed in the table are those values reported by the authors of the respective studies.
**Abbreviations:** CLDQ-HCV, Chronic Liver Disease Questionnaire-Hepatitis C Virus; CLDQ-MASH, Chronic Liver Disease Questionnaire-Metabolic Associated Steatohepatitis; CLDQ-PBC, Chronic Liver Disease Questionnaire-Primary Biliary Cirrhosis; HRQL, Health Related Quality of Life; MCID, Minimal Clinically Important Difference; MCS, Mental Component Score; NA, Not Available; PCS, Physical Component Score; QALY, Quality Adjusted Life Year; SF-36, Short Form-36; VAS, Visual Analogue Scale; ZBI-22, Zarit Burden Interview-22

### **Appendix S5**: Detailed mapping process for PRO instruments

**Table S5.1. Breakdown of each patient-reported outcome measure into its constituent items, mapped across domains**

| **Tool** | **Physical function** | **Pain** | **Fatigue** | **Sleep** | **Sexual function** | **Dyspnea** | **GI symptoms/itch/edema** | **Anxiety/Depression** | **Cognitive function** | **Uncertainity** | **Social Roles/Activities** | **Social isolation/Companionship** | **Support/Caregiving** | **Global Health rating** | **Calculated health utility** |
| --- | --- | --- | --- | --- | --- | --- | --- | --- | --- | --- | --- | --- | --- | --- | --- |
| **SF-36** | Physical function/Role Physical | Bodily pain | Vitality |  |  |  |  | Mental Health |  |  | Social functioning/Role Physical/Role Emotional |  |  | General Health/Vitality | SF-6D* |
| **EQ-5D-5L** | Mobility/Self-care/Usual activities | Pain/discomfort |  |  |  |  |  | Anxiety/Depression |  |  | Usual activities |  |  | EQ VAS | HU index |
| **CLDQ** | Activity | Abdominal/Systemic symptoms | Fatigue |  |  | Systemic symptoms | Abdominal/Systemic symptoms | Worry/Emotional Function |  | Worry | Activity |  |  |  |  |
| **SFLDQOoL (+ SF-36)** | Physical function/Role Physical/Effects of liver disease | Symptoms of liver disease/Bodily Pain | Vitality/Effects of liver disease | Sleep | Sexual problems | Symptoms of liver disease | Symptoms of liver disease | Mental Health/Hopelessness/Distress | Memory/concentration | Effects of liver disease/Stigma of liver disease | Effects of liver disease/Stigma of liver disease Social functioning/Role Physical/Role Emotional | Loneliness/Stigma of liver disease |  | General Health | SF-6D* |
| **SFLDQoL** | Effects of liver disease | Symptoms of liver disease | Effects of liver disease | Sleep | Sexual problems | Symptoms of liver disease | Symptoms of liver disease | Hopelessness/Distress | Memory/concentration | Effects of liver disease/Stigma of liver disease | Effects of liver disease/Stigma of liver disease | Loneliness/Stigma of liver disease |  |  |  |
| **MLHFQ** | Stairs/walking |  | Resting during day/Fatigue | Sleeping difficulty | Sexual activities | Shortness of breath | Eating less food/ankle swelling | Being depressed/Loss of self-control/Being worried | Difficulty concentrating or remembering |  | Away from home/Doing things with friends/Earning a living/Recreational activities/ | Feeling burden to family or friends /Doing things with friends |  |  |  |
| **Ascites-Q** | Limited mobility | Abdominal pain/Back pain | Tired | Problems with sleep |  | Shortness of breath | Fullness/Early satiety/Lack of appetite/Nausa |  |  |  |  |  |  |  |  |
| **ASI-7** | Restriction of activity/eating |  | Fatigue |  |  | Difficulty in breathing/breathing while walking | Abdominal bloating/Restriction on eating |  |  |  |  |  |  |  |  |
| **CAS-scale** | Difficult in mobility/disabled when dressing/trouble walking | Stomach pain/sore/uncomfortable/leg pain | Tired |  |  | Shortness of breath/breathing deeply | Nausea/appetite/change in stools |  |  |  | Disabled when dressing |  |  |  |  |
| **FACIT-AI** | To get around by myself | Pain | Lack of energy | Sleeping well |  | Shortness of breath | Appetite/Nausea/Vomiting/Early Satiety/Urinary frequency/ constipation | Emotional distressed |  |  | Ability to get around by myself |  |  |  |  |
| **IPOS** | Poor mobility | Pain | Weakness or lack of energy/Drowsiness |  |  | Shortness of breath | Nausea/Vomiting/Poor appetite/Constipation/Sore or dry mouth | Anxiety about illness/treatment/Depressed/Peace |  | Information sufficiency/Practical problems | Practical | Ability to share feelings | Family/friends worry |  |  |
| **ZBI-12** |  |  |  |  |  |  |  | Stressed/Angry/strained/uncertainty |  | Loss of control/Suffered | Social life/time for yourself | Negative impact on relationship with others | All items |  |  |
| **MUIS-A** |  |  |  |  |  |  |  |  |  | All items |  |  |  |  |  |
| **MUIS-FM** |  |  |  |  |  |  |  |  |  | All items |  |  |  |  |  |
| **Mutuality scale** |  |  |  |  |  |  |  |  |  |  | Enjoyment of social activities | All items | Emotional support/appreciation |  |  |
| **VAS Score** (QOL/Fatigue/ Physical Performance) | Physical performance |  | Fatigue |  |  |  |  |  |  |  |  |  |  | Quality of Life |  |
| **VAS** (QoL) |  |  |  |  |  |  |  |  |  |  |  |  |  | Quality of Life |  |

**Notes:** This process was completed by two reviewers independently and disagreements resolved through discussion. There was broad agreement, and only minor differences. The concept of social roles was broadened to include items that impacts activities of daily living. Sleep domain focused on disturbance rather than sleep related impairment (since the latter was already covered by fatigue and cognitive function)

### **Appendix S6:** Domain scores in the meta-analysis and extraction methodology

Domain-level scores extracted from each included study are presented below.
Standard errors were derived as SE = SD / SQRT(n).
Where studies reported median and interquartile range (IQR) instead of mean and SD, values were converted using the method of Luo et al.^7^ (2018), or that of Wan et al.^8^ (2014) where the former was not possible (see details below).

**Table S6.1. SF-36 domain scores by study, mean (SD)**

| **SF-36 domain** | **Stepanova 2018 (n=58)** | **Hansen 2022 (n=38)** | **Higuera-de la Tijera 2019 (n=100)** | **Macdonald 2018 (n=405)** |
| --- | --- | --- | --- | --- |
| **Physical functioning** | 40.5 (25.7) | 28.8 (9.6) | 29.6 (21.8) | 49.2 (24.0) |
| **Role physical** | 30.5 (25.9) | 27.1 (8.1) | 27.3 (25.7) | 11.7 (26.0) |
| **Bodily pain** | 53.3 (28.8) | 37.7 (9.2) | 16.9 (34.0) | 53.4 (24.5) |
| **General health** | 37.1 (18.6) | 32.6 (9.4) | 42.4 (24.1) | 40.2 (18.3) |
| **Vitality** | 40.2 (22.8) | 37.1 (12.0) | 47.6 (23.0) | 41.8 (19.0) |
| **Social functioning** | 53.9 (27.7) | 33.3 (12.8) | 41.5 (29.2) | 55.9 (22.4) |
| **Role emotional** | 50.1 (32.0) | 28.4 (13.3) | 39.0 (34.0) | 44.3 (74.4) |
| **Mental health** | 65.2 (21.4) | 45.9 (8.9) | 31.2 (15.4) | 60.3 (19.4) |

**Note:** Values shown are means (SD). SF-36 domain scale 0–100.
Stepanova (2018) values were obtained from published report
Hansen (2022) data were provided by authors after being contacted
Higuera-de la Tijera (2019) extracted from published graph using Plotdigitiser.
Macdonald (2018) values represent the combined population (n=405), pooled from two subgroups (diuretic-resistant n=164; diuretic-intractable n=241) by weighted averaging prior to conversion; detailed source data and methods in Table S5.3.

**Table S6.2. CLDQ domain scores by study, mean (SD)**

| **CLDQ domain** | **Stepanova 2018 (n=58)** | **Hanai 2022 (n=16)** | **Terbah 2024 (n=30)** |
| --- | --- | --- | --- |
| **Abdominal symptoms** | 3.93 (1.54) | 2.67 (1.48) | 3.67 (2.10) |
| **Activity** | 3.99 (1.54) | 3.53 (1.54) | 3.47 (1.92) |
| **Emotional function** | 4.59 (1.28) | 3.81 (0.97) | 3.72 (1.55) |
| **Fatigue** | 3.83 (1.37) | 3.07 (1.04) | 2.57 (0.96) |
| **Systemic symptoms** | 4.36 (1.26) | 3.93 (1.33) | 3.58 (1.59) |
| **Worry** | 4.22 (1.79) | 3.17 (1.56) | 2.88 (1.59) |

**Note:** Values shown are means (SD). CLDQ domain scale 1–7.
Stepanova (2018) values were obtained from published report
Hanai (2022) and Terbah (2024) values were converted from published median/IQR using the Wan et al. method as ranges were not available.

**Table S6.3. Macdonald 2018 source data and conversion to mean (SD)**

| **SF-36 domain** | **Diuretic-resistant (n=164) median (IQR)** | **Diuretic-intractable (n=241) median (IQR)** | **Combined (n=405) median (IQR)** | **Min** | **Max** | **Method** |
| --- | --- | --- | --- | --- | --- | --- |
| **Physical functioning** | 45 (30–67) | 50 (30–70) | 48 (30–69) | 0 | 100 | Luo |
| **Role physical** | 0 (0–50) | 0 (0–25) | 0 (0–35) | 0 | 63 | Wan |
| **Bodily pain** | 52 (31–80) | 61 (31–74) | 57 (31–76) | 0 | 100 | Luo |
| **General health** | 40 (30–55) | 39 (25–52) | 39 (27–53) | 0 | 90 | Luo |
| **Vitality** | 40 (30–55) | 40 (25–55) | 40 (27–55) | 0 | 92 | Luo |
| **Social functioning** | 63 (38–75) | 50 (38–75) | 55 (38–75) | 0 | 100 | Luo |
| **Role emotional** | 33 (0–100) | 33 (0–100) | 33 (0–100) | 0 | 100 | Wan |
| **Mental health** | 60 (48–76) | 62 (44–76) | 61 (46–76) | 8 | 100 | Luo |

**Note:** Subgroup median (IQR) values were combined by weighted averages (n=405).
Minimum and maximum values were extracted from published violin plots using PlotDigitizer.
The ‘Method’ column indicates the formula used to obtain a mean and standard deviation/standard error: Luo method was used for most domains except for Role physical (where minimum value = Q1 = median = 0) and Role emotional (where minimum = Q1 = 0 and maximum = Q3 = 100).^7^ In both those instances the Luo method was not applicable, and the Wan method was used instead.^8^

### **Appendix S7:** Forest plots of individual domains from the meta-analysis

CLDQ:

### SF-36:

## **Supplementary References**

(1) Desai AP, Orman ES, Aridi TG, et al. Meaningful differences in patient-reported outcome measurement scores in liver disease. Hepatol Commun 2025;9:e0727.
(2) Gonzalez-Saenz de Tejada M, Bilbao A, Ansola L, Bermúdez-Ampudia C, Escobar A, Esteban C, Garcia-Gutierrez S, Lafuente I, Quintana JM. Responsiveness and minimal clinically important difference of the Minnesota living with heart failure questionnaire. Health Qual Life Outcomes. 2019 Feb 7;17(1):36.
(3) Kanwal F, Spiegel BMR, Hays RD, et al. Prospective validation of the short form liver disease quality of life instrument. Aliment Pharmacol Ther 2008;28:1088-1101.
(4) Neijenhuis M, Gevers TJG, Atwell TD, et al. Development and validation of a patient-reported outcome measurement for symptom assessment in cirrhotic ascites. Am J Gastroenterol 2018;113:567-575.
(5) Younossi ZM, Stepanova M, Schattenberg JM, et al. Measuring health-related quality of life and patient-reported outcomes in chronic liver disease. J Hepatol. 2026;84; 457-472
(6) Younossi ZM, Kremer AE, Swain MG,  et al.  Assessment of fatigue and its impact in chronic liver disease. J Hepatol. 2024;81:726-742
(7) Luo D, Wan X, Liu J, Tong T. Optimally estimating the sample mean from the sample size, median, mid-range, and/or mid-quartile range. Stat Methods Med Res. 2018;27(6):1785–1805.
(8) Wan X, Wang W, Liu J, Tong T. Estimating the sample mean and standard deviation from the sample size, median, range and/or interquartile range. BMC Med Res Methodol. 2014;14:135.
